# Modeling Mitochondrial Bioenergetics with Integrated Volume Dynamics

**DOI:** 10.1371/journal.pcbi.1000632

**Published:** 2010-01-01

**Authors:** Jason N. Bazil, Gregery T. Buzzard, Ann E. Rundell

**Affiliations:** 1 Weldon School of Biomedical Engineering, Purdue University, West Lafayette, Indiana, United States of America; 2 Department of Mathematics, Purdue University, West Lafayette, Indiana, United States of America; Medical College of Wisconsin, United States of America

## Abstract

Mathematical models of mitochondrial bioenergetics provide powerful analytical tools to help interpret experimental data and facilitate experimental design for elucidating the supporting biochemical and physical processes. As a next step towards constructing a complete physiologically faithful mitochondrial bioenergetics model, a mathematical model was developed targeting the cardiac mitochondrial bioenergetic based upon previous efforts, and corroborated using both transient and steady state data. The model consists of several modified rate functions of mitochondrial bioenergetics, integrated calcium dynamics and a detailed description of the K^+^-cycle and its effect on mitochondrial bioenergetics and matrix volume regulation. Model simulations were used to fit 42 adjustable parameters to four independent experimental data sets consisting of 32 data curves. During the model development, a certain network topology had to be in place and some assumptions about uncertain or unobserved experimental factors and conditions were explicitly constrained in order to faithfully reproduce all the data sets. These realizations are discussed, and their necessity helps contribute to the collective understanding of the mitochondrial bioenergetics.

## Author Summary

Mathematically modeling biological systems challenges our current understanding of the physical and biochemical events contributing to the observed dynamics. It requires careful consideration of hypothesized mechanisms, model development assumptions and details regarding the experimental conditions. We have adopted a modeling approach to translate these factors that explicitly considers the thermodynamic constraints, biochemical states and reaction mechanisms during model development. Such models have numerous constant parameters that must be determined. Integrating thermodynamics and detailed mechanistic representation of the principal phenomena help constrain these parameter values; therefore, only a handful of the total number of model parameters (∼10%) must be adjusted during parameter estimation through model simulations. Additionally, all models must undergo some form of corroboration prior to application. In practice, this corroboration should challenge all possible dynamics of the model, but it is recognized that in this data rich world, we are surprisingly data poor. Eventually such developed and corroborated models are capable of supporting current hypotheses, guiding experimental designs and contributing to the overall knowledge base of biological processes.

## Introduction

### Motivation for Model Study

The simulation of mathematical models of mitochondrial bioenergetics provides a powerful analytical alternative to performing numerous exhaustive experiments. Such models aid in the interpretation of experimental data and facilitate experimental design for elucidating the supporting biochemical and physical processes. Current experimental techniques limit the ability to resolve details of the mitochondrial bioenergetic processes *in vivo*. Specifically, many different chemical species and events must be simultaneously monitored to track the profusion of enzymatic reactions involved in the tricarboxylic acid (TCA) cycle, β-oxidation, the electron transport system (ETS), ATP synthesis and electrolyte dynamics. Currently, it is impossible to accurately and simultaneously measure all of these enzymatic processes *in vivo* with any degree of precision. Nevertheless, a plethora of experimental data is available on mitochondrial bioenergetics that was collected using a variety of techniques, experimental conditions, and tissue sources. No existing experimental data set is complete that consists of measurements of all of the supporting chemical species and events; therefore, the correct interpretation of the available experimental data in isolation or as a collective unit is difficult. This requires careful consideration of all potential data-consistent dynamics of the unobserved species and events. To aid in the interpretation, a quantitative framework established via mathematical model development and parameter identification through experiment simulation is commonly employed in a collective manner so that a data-compatible, semi-mechanistic description of the underlying bioenergetic processes emerges. With the addition of each new experimental data set, the model matures either through corroboration via evidence that supports the hypothesized mechanisms encoded within the mathematical representation or through modification of the model structure and parameters to refine and/or reveal more insights and alter the supporting hypothesized mechanisms underlying bioenergetic processes.

Several mathematical models have been developed [1]–[6] to describe aspects of mitochondrial bioenergetics, but none currently capture the complete dynamics of all metabolically relevant ion and substrate regulatory functions observed during physiological and pathophysiological conditions. As the next step towards constructing a complete physiologically faithful mitochondrial bioenergetics model, this manuscript describes the development and corroboration of a mathematical model based on cardiac mitochondria that builds upon these previously published models. The Yugi and Tomita model [6] simulates mitochondrial bioenergetics with the most breadth. They have compiled a large mitochondrial bioenergetics model that qualitatively captures the basic mitochondrial bioenergetic phenomena and included much of the mitochondrial biochemistry from a variety of species and organs. Although the Yugi and Tomita model has been successfully modified to predict the dynamic response of β-oxidation in the context of human disease [7], it does not incorporate some details required to reproduce a few specific bioenergetic regulatory features of interest herein. The Wu et al. model [1] was chosen as the base model for this work due to its meticulous attention to thermodynamics and inclusion of the various biochemical species present in the mitochondrial milieu. Our model structure and the parameter values were selected so that simulations of the experimental conditions on porcine or rat heart mitochondria simultaneously fit the inorganic phosphate (Pi) control over mitochondrial bioenergetics [8] and TCA intermediate dynamics [9] data sets upon which the Wu et al. model was developed as well as additional experimental data on the extra-mitochondrial calcium-dependent steady state matrix calcium concentrations [10] and mitochondrial matrix volume dynamics [11]. Numerous other experimental studies were used to fit constants for the employed rate expressions as described in the Supplemental Material (Text S1). The resulting extended model is corroborated with additional experimental data on the steady state behavior of the TCA cycle [9], volume dynamics under various bioenergetic/pharmacological interventions [11] and the bioenergetic/volume responses of mitochondria to variations in buffer osmolarity [12]; a local sensitivity analysis was also used to explore the robustness of the model structure relative to these simulated experiments. This manuscript describes all phases of this process including the model development, parameter estimation and corroboration. It concludes with a discussion of the insights on the bioenergetic processes obtained during the model extension.

## Results

### Model Development

**Figure 1:**
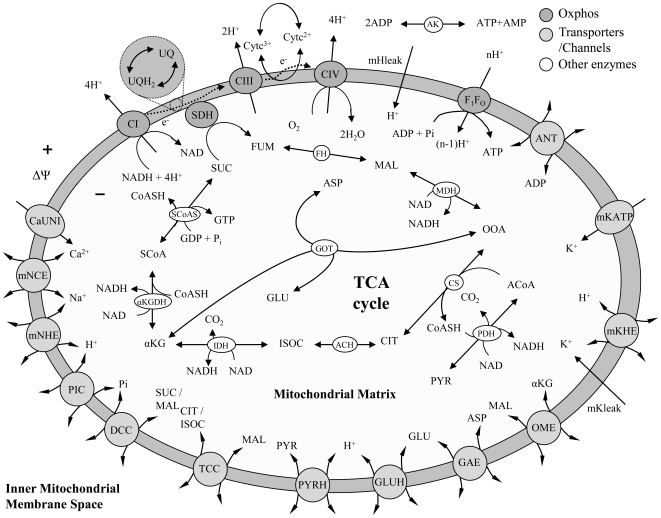
A graphical representation of the bioenergetic elements and processes described by the model. Abbreviations: Oxphos, oxidative phosphorylation elements; PYR, pyruvate; CoASH, coenzyme A; AcCoA, acetyl-coenzyme A; CIT, citrate; ISOC, isocitrate; αKG, α-ketoglutarate; SCoA, succinyl CoA; SUC, succinate; FUM, fumarate; MAL, malate; OAA, oxaloacetate; GLU, glutamate; ASP, aspartate; NADH, reduced nicotinamide adenine dinucleotide; NAD, GTP, guanidine triphosphate; GDP, guanidine diphosphate; oxidized nicotinamide adenine dinucleotide; Pi, inorganic phosphate; UQ, ubiquinone; UQH_2_, ubiquinol; Cytc^3+^, oxidized cytochrome c; Cytc^2+^, reduced cytochrome c; PDH, pyruvate dehydrogenase; CS, citrate synthase; ACH, acontinase; IDH, isocitrate dehydrogenase; αKGDH, α-ketoglutarate dehydroganse; SCoAS; succinyl CoA synthetase; SDH, succinate dyhdrogenase; FH, fumarate hydratase; MDH, malate dehydrogenase; GOT, glutamate oxaloacetate transaminase; CI, Complex I; CIII, Complex III; CIV, Complex IV; mHleak, proton leak; F_1_F_O_, F_1_F_O_ ATP synthase; ANT, adenine nucleotide transporter; PIC, inorganic phosphate carrier; GAE, glutamate/aspartate exchanger; OME, α-ketoglutarate/malate exchanger; DCC, dicarboxylate carrier; TCC, tricarboxylate carrier; PYRH, pyruvate-proton cotransporter; GLUH, glutamate-proton cotransporter; mKATP, ATP-dependent K^+^ channel; mKHE, K^+^/H^+^ exchanger; mKleak, K^+^ leak; mNHE, Na^+^/H^+^ exchanger; mNCE, Na^+^/Ca^2+^ exchanger; CaUNI, Ca^2+^ uniporter; AK, adenylate kinase.

The model integrates mitochondrial bioenergetic processes as shown in Figure 1 including oxidative phosphorylation, the ETS, the TCA cycle and related reactions, the Na^+^/Ca^2+^ cycle and the K^+^-cycle. To maintain thermodynamic consistency, all reactions in the model, as shown in Table 1, are represented as thermodynamically balanced and reversible. (It should be noted that some of the reactions could have been treated as irreversible reactions since the conditions necessary to reverse them lie very far from physiological conditions.) The model is primarily based on the mitochondrial bioenergetics model proposed by Wu et al. [1] and extended to address: 1) updated formalisms for the calcium-sensitive dehydrogenases, as well as, several modified TCA cycle related rate expressions; 2) a two-site model for the adenine nucleotide transporter [13]; 3) the Na^+^/Ca^2+^ cycle including a non-linear dependence on inner mitochondrial membrane potential difference, ψ_inside_ - ψ_outside_, (Δψ) for the calcium uniporter (CaUNI) [14], magnesium inhibition kinetics for the CaUNI, and the proton-regulation of the mitochondrial Na^+^/H^+^ exchanger (mNHE); and 4) the K^+^-cycle including the mitochondrial ATP-dependent K^+^ channel (mKATP), electrophoretic K^+^-leak and the mitochondrial K^+^/H^+^ exchanger (mKHE) with the mitochondrial matrix volume regulation dynamics hypothesized by Garlid [15]. Each addition is described in the following paragraphs.

**Table 1:** Modeled Mitochondrial Bioenergetic Reactions.

Reaction	Enzyme	Biochemical Reaction
	*Mitochondrial Reactions*	
J_PDH_	Pyruvate dehydrogenase	PYR + CoASH + NAD + H_2_O ↔ CO_2_ + SCOA + NADH
J_CS_	Citrate synthase	OAA + AcCoA + H_2_O ↔ CoASH + CIT
J_ACH_	Aconitase	CIT ↔ ISOC
J_IDH_	Isocitrate dehydrogenase	NAD + ISOC + H_2_O ↔ αKG + NADH + CO_2_
J_aKGDH_	α-Ketoglutarate dehydrogenase	αKG + CoASH + NAD + H_2_O ↔ CO_2_ + SCoA + NADH
J_SCoAS_	Succinyl CoA synthase	SCoA + GDP + Pi ↔ SUC + GTP + CoASH
J_SDH_	Succinate dehydrogenase	SUC + UQ ↔ UQH_2_ + FUM
J_FH_	Fumarate hydratase	FUM + H_2_O ↔ MAL
J_MDH_	Malate dehydrogenase	NAD + MAL ↔ OAA + NADH
J_NDK_	Nucleoside diphosphokinase	GTP + ADP ↔ GDP + ATP
J_GOT_	Glutamate oxaloacetate transaminase	ASP + αKG ↔ OAA + GLU
J_CI_	Complex I	NADH + UQ ↔ NAD + UQH_2_
J_CIII_	Complex III	UQH_2_ + 2Cytc^3+^ ↔ UQ + 2Cytc^2+^
J_CIV_	Complex IV	2Cytc_red_ + ½O_2_ ↔ 2Cytc_ox_ + H_2_O
J_F1FO_	F_1_F_O_ ATP synthase	ADP + Pi ↔ ATP + H_2_O
J_AK_	Adenylate Kinase	2ADP ↔ ATP + AMP
	*Exchangers and Ion Channels*	
J_GAE_	Glutamate-aspartate exchanger	GLU_ims_ + H^+^_ims_ + ASP_mtx_ ↔ GLU_mtx_ + H^+^_mtx_ + ASP_ims_
J_OME_	α-Ketoglutarate-malate exchanger	αKG_ims_ + MAL_mtx_ ↔ αKG_mtx_ + MAL_ims_
J_PYRH_	Pyruvate-proton cotransporter	PYR_ims_ + H^+^_ims_ ↔ PYR_mtx_ + H^+^_mtx_
J_GLUH_	Glutamate-proton cotransporter	GLU_ims_ + H^+^_ims_ ↔ GLU_mtx_ + H^+^_mtx_
J_CITMAL_	Tricarboxylate carrier	CIT_ims_ + MAL_mtx_ ↔ CIT_mtx_ + MAL_ims_
J_ISCOMAL_	Tricarboxylate carrier	ISOC_ims_ + MAL_mtx_ ↔ ISOC_mtx_ + MAL_ims_
J_SUCPi_	Dicarboxylate carrier	SUC_mtx_ + Pi_ims_ ↔ SU C_ims_ + Pi_mtx_
J_MALPi_	Dicarboxylate carrier	MAL_mtx_ + Pi_ims_ ↔ MAL_ims_ + Pi_mtx_
J_ANT_	Adenine nucleotide transporter	ATP_mtx_ + ADP_ims_ ↔ ATP_ims_ + ADP_mtx_
J_PIC_	Inorganic phosphate carrier	Pi_ims_ + H^+^_ims_ ↔ Pi_mtx_ + H^+^_mtx_
J_mHleak_	Proton leak	H^+^_ims_ ↔ H^+^_mtx_
J_mKHE_	Potassium-hydrogen exchanger	H^+^_ims_ + K^+^_mtx_ ↔ K^+^_ims_ + H^+^_mtx_
J_mKATP_	ATP-dependent potassium channel	K^+^_ims_ ↔ K^+^_mtx_
J_mKleak_	Potassium leak	K^+^_ims_ ↔ K^+^_mtx_
J_CaUNI_	Calcium uniporter	Ca^2+^_ims_ ↔ Ca^2+^_mtx_
J_mNCE_	Sodium-calcium exchanger	Ca^2+^_mtx_ + 3Na^+^_ims_ ↔ Ca^2+^_ims_ + 3Na^+^_mtx_
J_mNHE_	Sodium-hydrogen exchanger	Na^+^_mtx_ + H^+^_ims_ ↔ Na^+^_ims_ + H^+^_mtx_
	*Other Reactions Used in the Model*	
J_HK_	Hexokinase^a^	GLC + ATP ↔ G6P + ADP

*abbrev:* mtx, matrix; ims, intermembrane space.

a Reaction is not presented in Figure 1.

The model proposed in the manuscript is a 73 state system of differential-algebraic equations (DAEs) that consists of 65 non-linear ordinary differential equations (ODEs); five algebraic conservation expressions to compute matrix ATP, guanidine triphosphate (GTP), reduced nicotinamide adenine dinucleotide (NADH), ubiquinol (UQH_2_) and reduced cytochrome c (c^2+^); one algebraic expression to compute matrix water volume; one algebraic expression to compute inner membrane space (IMS) water volume and one algebraic expression to compute matrix chloride content (Cl^−^). The majority of the experimental data used to parameterize the model proposed in this manuscript were derived from heart tissue of either bovine, porcine or rat with some data obtained from liver tissue. Part S1 of the Supplemental Material (Text S1) lists the state variables and general parameters, Part S2 contains the system of DAEs comprising the model and Part S3 provides a detailed description of the rate expressions and their parameters used to construct the system of DAEs. Part S3 also includes the fitness for some rate expressions calibrated with additional experimental data not explicitly indicated in this manuscript.

The TCA and related enzyme rate expressions are structurally identical to Wu et al. except for a few alterations. To include the calcium-dependence of matrix dehydrogenases, the rate expressions for pyruvate dehydrogenase (PDH), isocitrate dehydrogenase (IDH) and α-ketoglutarate dehydrogenase (αKGDH) were modified. PDH, an important regulatory enzyme involved with mitochondrial bioenergetics, is responsible for the oxidative decarboxylation of pyruvate, transacylation of an acetyl group to CoA and production of reducing equivalents for the ETS. A similar rate expression found in Wu et al. was used with a few notable modifications. The proton, divalent cation and adenine nucleotide regulatory mechanisms were inserted into the expression to reproduce the available data [16]. IDH is responsible for the oxidative decarboxylation of isocitrate to produce α-ketoglutarate and reducing equivalents for the ETS. The rate expression used in the model is from Qi et al. [17]. The key TCA regulatory enzyme, αKGDH, is responsible for the oxidative decarboxylation of α-ketoglutarate transferring a succinyl group to CoA and producing reducing equivalents for the ETS. The consensus hexa-uni-ping-pong mechanism with the appropriate activation and inhibition modifications was used to reproduce a wide variety of data from four independent data sets [18]–[21].

Two additional rate expressions that deviated from Wu et al. are the glutamate-aspartate exchanger (GAE) and dicarboxylate carrier (DCC). The GAE is a key enzyme in the malate-aspartate shuttle and is particularly important maintaining state 3 NADH levels when mitochondria respire on glutamate and malate. This electrogenic exchanger is activated by calcium and swaps glutamate and a proton with aspartate taking advantage of the energized state of mitochondria established by the ETS. The enzyme reaction was modeled based on a rapid equilibrium bi-bi mechanism with a third substrate, protons, added to the rate expression; it was fit to data from bovine heart mitochondria [22]–[23]. The DCC exchanges TCA cycle intermediates malate, succinate and hydrogen phosphate. This exchanger was also modeled as forming a ternary complex with its substrates, and the kinetic parameters were fit to rat liver mitochondrial experimental data [24]–[25].

The ANT is the enzyme responsible for exchanging unchealated ATP and ADP across the mitochondrial inner membrane. Previous models used a ping-pong mechanism that employed a single adenine nucleotide binding site whereby Δψ affected only ATP binding [26]. This type of mechanism has been shown to inadequately describe the enzyme kinetics, and studies have identified at least two distinct adenine nucleotide binding sites [27]–[28]. The model presented by Metelkin et al. [13] addresses these issues and was therefore chosen to describe the ANT kinetics. The parameters were refit to the original data to include the effect of Na^+^ and K^+^ chelation of adenine nucleotides.

The model proposed in this manuscript incorporates mitochondrial calcium dynamics similar to Nguyen et al. [2], Cortassa et al. [3] and Dash and Beard [14]. The CaUNI is similar to the expression found in Dash and Beard including the nonlinear dependence on Δψ with the addition of explicit magnesium inhibition based on experimental data. In Part S3 of the Supplemental Material (Text S1), the magnesium inhibition was used to show that a single calcium dissociation constant with magnesium acting as a competitive inhibitor against calcium binding for the channel is capable of reproducing the experimental data (consisting of both rat heart and liver mitochondria). The mNCE is similar to [2]–[3],[14] with the noted addition of a hypothetical matrix calcium activation mechanism. This calcium activation mechanism resulted in comparable dynamics under the calcium loading experiments used to fit Dash and Beard's matrix calcium inhibition mechanism for the CaUNI but was extended to be analogous with the current experimental evidence regarding the sarcolemmal isoform [29]. The mNHE was slightly modified from Nguyen et al. to include a hill coefficient of 2 for the proton regulation mechanism.

The ‘futile’ K^+^-cycle plays a major role in mitochondrial volume homeostasis [30]–[34]. Potassium influx via the mKATP and electrophoretically driven potassium uptake via leak pathways must be balanced with potassium efflux from the mKHE. Originally, the mKHE was thought to be regulated by the carrier brake hypothesis [35]. This essentially involves some endogenous element in the matrix, such as magnesium, that serves as the “carrier brake” that is reversibly released by matrix swelling. Brierley and Jung call into question this hypothesis noting that under physiological conditions, the known inhibitors of the exchanger are present at concentrations much greater than their respective inhibitory constants [36]. Garlid then proposed that the mKHE is additionally regulated by matrix volume with membrane stretching activating the exchanger [15]. Further evidence for this mechanism is provided by the results of the analysis of the mathematical model proposed in this manuscript discussed below.

### Parameter Estimation

To parameterize the model, four independent data sets consisting of 32 data curves were used from Bose et al. [8], LaNoue et al. [9], Wan et al. [10] and Kowaltowski et al. [11]. In the manuscript these studies will be henceforth referenced as the Bose data set, LaNoue data set, Wan data set and Kowaltowski data set, respectively. For each data set, the model was initialized from a condensed, fully oxidized and de-energized state via initialization simulations that replicated the experimental incubation conditions (i.e. Pi- and Ca^2+^-depletion) prior to parameter estimation. The parameter identification was subsequently conducted using simulation conditions closely mimicking those of the experimental methods. The resulting values of the 42 adjustable parameters, their definitions, their best fit values and their associated normalized local sensitivity coefficients (LSC) are provided in Table 2. The following paragraphs describe the ability of the model with these fitted parameter values to reproduce the experimental results from these four independent experimental data sets; pertinent details of the experimental conditions and their replication through model simulations are described in the Methods section.

**Table 2:** Description of Adjustable Parameters (T = 25°C).

Parameter	Definition	Value (LSCx100)	Units
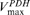	Pyruvate dehydrogenase max rate	127 (4.88)	nmol/min/mg
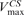	Citrate synthase max rate	584 (1.64)	nmol/min/mg
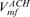	Acontinase max forward rate	1.16×10^5^ (0.011)	nmol/min/mg
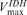	Isocitrate dehydrogenase max rate	6.84×10^4^ (0.341)	nmol/min/mg
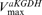	α-Ketoglutarate dehydrogenase max rate	779 (2.18)	nmol/min/mg
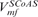	Succinyl CoA synthase max forward rate	3.93×10^4^ (0.216)	nmol/min/mg
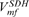	Succinate dehydrogenase max forward rate	7.00×10^3^ (2.26)	nmol/min/mg
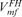	Fumurate hydratase max forward rate	7.67×10^5^ (0.010)	nmol/min/mg
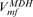	Malate dehydrogenase max forward rate	965 (0.432)	nmol/min/mg
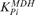	Malate dehydrogenase Pi binding constant	5.00×10^−3^ (0.156)	M
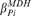	Malate dehydrogenase Pi activation constant	57.7 (0.193)	unitless
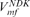	Nucleoside diphosphokinase max forward rate	6.95×10^3^ (0.016)	nmol/min/mg
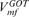	Glutamate oxaloacetate transaminase max forward rate	1.09×10^6^ (0.251)	nmol/min/mg
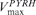	Pyruvate-hydrogen co-transporter activity	3.12×10^13^ (0.651)	nmol/M^2^/min/mg
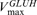	Glutamate-hydrogen co-transporter activity	9.44×10^10^ (0.763)	nmol/M^2^/min/mg
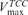	Tricarboxylate activity	7.13×10^8^ (0.471)	nmol/M^2^/min/mg
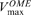	α-Ketoglutarate-malate exchanger max forward rate	8.83×10^3^ (0.275)	nmol/min/mg
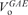	Glutamate-aspartate exchanger un-stimulated max rate	674 (0.865)	nmol/min/mg
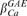	Glutamate-aspartate calcium activation constant	27.2 (0.299)	unitless
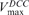	Dicarboxylate carrier max rate	8.03×10^3^ (0.901)	nmol/min/mg
*V_CI_*	Complex I activity	5.63×10^6^ (0.962)	nmol/M^2^/min/mg
*V_CIII_*	Complex III activity	5.84×10^5^ (2.19)	nmol/M^3/2^/ming/mg
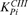	Complex III Pi binding constant	4.40×10^−3^ (0.976)	M
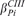	Complex III Pi activation constant	148 (1.54)	unitless
*V_CIV_*	Complex IV activity	44.0 (2.19)	nmol/M/min/mg
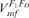	F_1_F_O_ ATP synthase activity	1.91×10^7^ (0.027)	nmol/M/min/mg
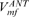	Adenine nucleotide translocase max rate	365 (7.52)	nmol/min/mg
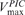	Inorganic phosphate carrier max rate	7.59×10^7^ (0.012)	nmol/min/mg
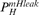	Proton permeability	8.06×10^7^ (5.01)	nmol/M/min/mg
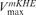	Potassium-hydrogen exchanger max rate	6.25 (3.58)	nmol/nl/min/mg
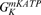	ATP-dependent potassium channel conductance	6.75 (0.181)	nmol/min/mg
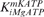	ATP-dependent potassium channel MgATP inhibition constant	4.10×10^−9^ (0.061)	M
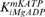	ATP-dependent potassium channel MgADP inhibition constant	10.0×10^−6^ (0.063)	M
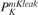	Potassium permeability	13.8 (4.87)	nmol/M/min/mg
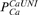	Calcium uniporter calcium permeability	157 (0.403)	nmol/min/mg
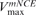	Sodium-calcium exchanger max rate	0.731 (0.360)	nmol/min/mg
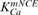	Sodium-calcium exchanger calcium binding constant	4.17×10^−6^ (0.326)	M
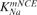	Sodium-calcium exchanger sodium binding constant	1.72×10^−3^ (0.018)	M
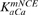	Sodium-calcium exchanger calcium activation binding constant	1.66×10^−6^ (0.238)	M
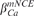	Sodium-calcium exchanger calcium activation constant	67.6 (0.296)	unitless
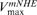	Sodium-hydrogen exchanger max rate	9.45×10^5^ (0.061)	nmol/min/mg
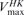	Hexokinase max rate	1.20×10^5^ (5.14)	nmol/min/mg

*abbrev:* LSC, local sensitivity coefficient (Note, the LSC is an average of the absolute value 1^st^ order sensitivity of a parameter with respect to all state variables, time points and experimental conditions used in this study. It is explicitly defined in Equation 2. In brief, a δ change in a given parameter translates to a δLSC change in the model output defined in the Sensitivity Analysis section of the Methods.).

**Figure 2:**
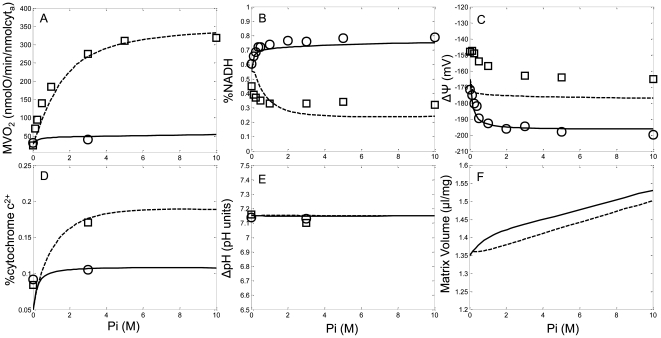
Model simulations (lines) of the Pi control exerted over mitochondrial bioenergetics shown in comparison with isolated mitochondria experimental data (symbols) for the conditions outlined in Bose et al. [8]. Mitochondria were incubated in the assay buffer identified in the Methods section under the Bose data set description. State 2 simulation results and experimental data are shown as solid lines and circles, respectively. State 3 simulations results and experimental data are shown as dotted lines and squares, respectively. The simulated Pi-titration response is shown with the experimentally measured data for the A) MVO_2_, B) redox state (percent NADH), C) Δψ, D) reduced cytochrome c (percent c^2+^), E) matrix pH and F) the simulated matrix volume.

The NADH-linked respiration components of the model were fitted against the Pi-titration experiments performed by Bose et al. [8]. In their manuscript, the authors reported a rich bioenergetic data set using glutamate/malate energized, Pi-depleted mitochondria under both state 2 and state 3 respiration conditions. State 3 was initiated and maintained with a sufficient bolus addition of 1.3 mM ADP. At this concentration, maximum respiration was maintained for at least half a minute before the ANT exerted its control due to limited substrate availability. Mitochondrial Δψ, NAD/NADH redox state, myocardial oxygen consumption (MVO_2_), cytochrome c^3+^/c^2+^ redox state and matrix pH were reported as the extra-mitochondrial Pi was progressively increased from 0 to 10 mM. Figure 2 shows that the model was capable of fitting this data set. Similar to the Wu et al. model, the model presented in this manuscript produced state 3 Δψ that reproduced the Pi-titration trend but at 10–15 mV lower than the experimentally measured state 3 Δψ. To achieve the low matrix pH levels observed experimentally, the mKHE was temporarily replaced with an expression with sufficient activity to rapidly equilibrate [K^+^]_mtx_[H^+^]_ims_/[K^+^]_ims_/[H^+^]_mtx_, and the initial matrix [K^+^] was adjusted to approximately 125 mM. Without these modifications for this data set, the model produced matrix pH of up to 7.2–7.5 pH units under the experimental conditions simulated. This issue is further explored in the Discussion. Overall, the experimentally reported Δψ, NAD/NADH redox state and MVO_2_ Pi-titrations as well as the expected increase in volume with increasing Pi are captured by the model.

**Figure 3:**
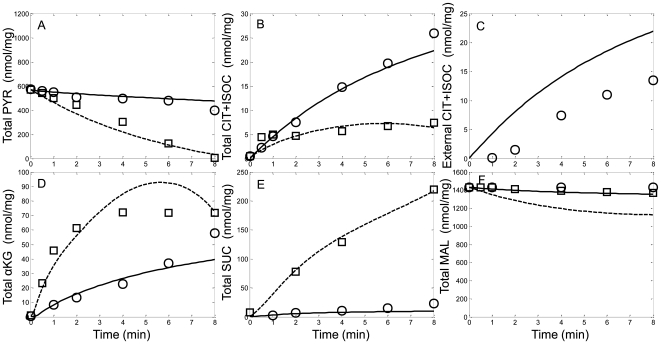
Model simulations (lines) of pyruvate/malate supported respiration on the levels of various TCA cycle intermediates compared with isolated mitochondria experimental data (symbols) for the conditions outlined in LaNoue et al. [9]. Mitochondria were incubated in the assay buffer identified in the Methods section under the LaNoue data set description. State 2 simulation results and experimental data are shown as solid lines and circles, respectively. State 3 simulations results and experimental data are shown as dotted lines and squares, respectively. The simulated TCA intermediate dynamics is shown with the experimentally measured data for A) the pyruvate utilization, B) the accumulation of lumped citrate and isocitrate, C) the accumulation of extra-mitochondrial lumped citrate and isocitrate, D) the accumulation of α-ketoglutarate, E) the accumulation of succinate and F) the residual malate content.

**Figure 4:**
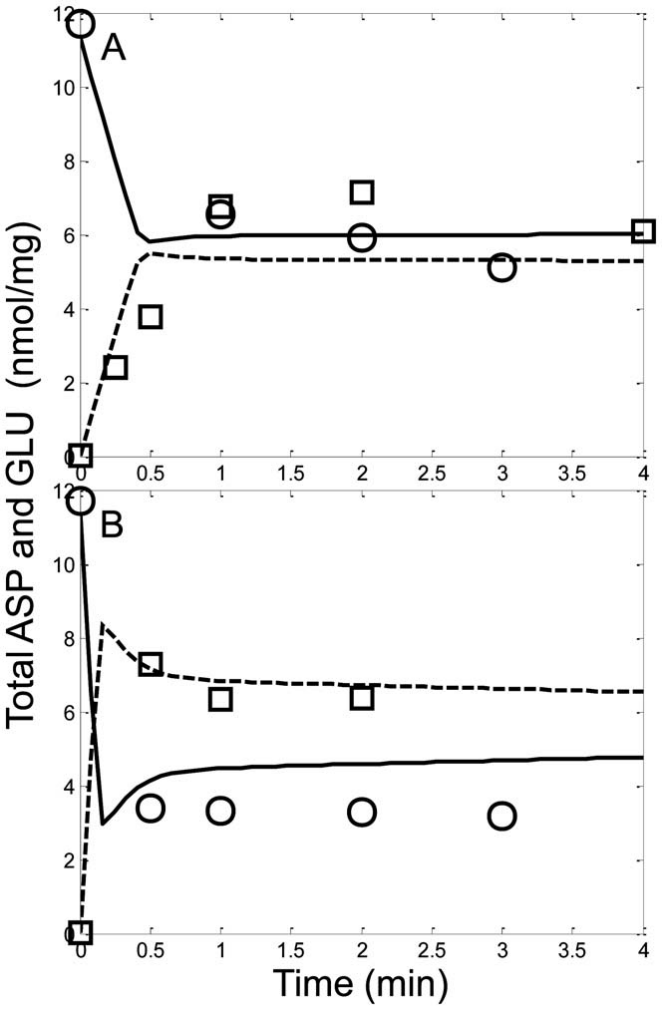
Model simulations (lines) of pyruvate supported respiration on the levels of the amino acids aspartate and glutamate in comparison with isolated mitochondria experimental data (symbols) for the conditions outlined in LaNoue et al. [9]. Mitochondria were incubated in the assay buffer identified in the Methods section under the LaNoue data set description. The simulated accumulation of aspartate (solid line) and glutamate (dotted line) are shown with experimentally measured aspartate (circles) and glutamate (squares). A) State 2, B) State 3.

The TCA cycle intermediate dynamics of the model were fitted to the data set presented by LaNoue et al. [9]. In their experiments, they used pyruvate/malate and pyruvate energized mitochondria in both state 2 and state 3 respiration. State 3 was initiated by the addition of 0.5 mM ADP and maintained using a hexokinase trap. They reported detailed time series data of most of the TCA cycle intermediates for each experimental condition. The model was able to capture the salient features of the pyruvate/malate energized mitochondrial TCA cycle intermediate dynamics as shown in Figure 3. The model simulated pyruvate, citrate, isocitrate, α-ketoglutarate, succinate and malate transients similar to the experimental data. Figure 4 shows that the simulated aspartate and glutamate dynamics using pyruvate energized mitochondria under both state 2 and state 3 respiratory conditions were also consistent with experimental data. In these simulations, the endogenous matrix aspartate content provided sufficient amino acid substrates for glutamate oxaloacetate transaminase (GOT) while glutamate efflux via glutamate-H^+^ cotransporter reduced the total available matrix asparate/glutamate pool.

**Figure 5:**
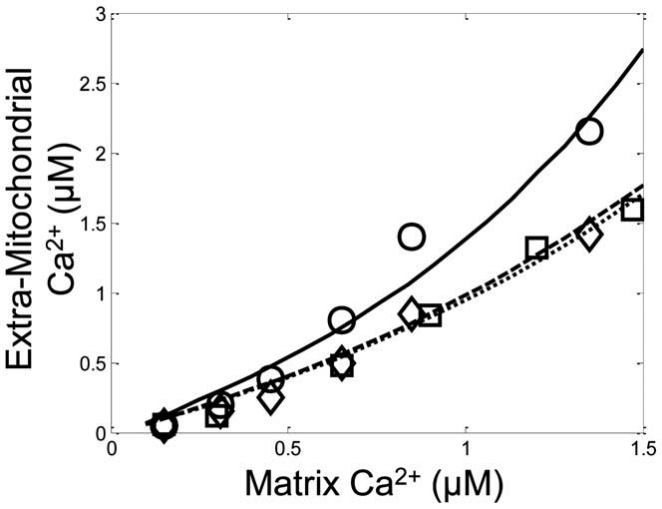
Model simulations (lines) of the steady state matrix free calcium relationship with respect to varying extra-mitochondrial calcium levels in comparison with isolated mitochondria experimental data (symbols) for the conditions outlined in Wan et al. [10]. Mitochondria were incubated in the assay buffer identified in the Methods section under the Wan data set description. The steady state matrix free calcium concentration is shown versus varying extra-mitochondrial calcium in the presence of 2 (solid line, circles), 5 (dashed, squares) and 20 (dotted line, diamonds) mM NaCl with 5 mM MgCl_2_.

The Na^+^/Ca^2+^ cycle was fitted to the steady state data from Wan et al. [10]. In their experiments, they used ATP-energized mitochondria and monitored steady state Ca^2+^ levels at varying extra-mitochondrial Ca^2+^, Na^+^ and Mg^2+^ concentrations. The ATP initialized the mitochondrial Δψ to approximately −120 mV in the model simulations corroborating the data reported by Territo et al. [37]. The model's capability of fitting the extra-mitochondrial Ca^2+^- and Na^+^-dependence on matrix steady state Ca^2+^ concentrations is illustrated in Figure 5. The steady sate matrix free Ca^2+^ data in the absence of extra-mitochondrial Na^+^ is not shown nor was used in the parameter estimation, because the Na^+^-independent calcium efflux was not included in the model structure. (Note, with an electroneutral Ca^2+^/2H^+^ exchange mechanism, the Na^+^-independent steady state matrix Ca^2+^ data could be reproduced by the model; however, this mechanism was not included in the current model formulation. The rationale for this choice is described in more detail in the Discussion.)

**Figure 6:**
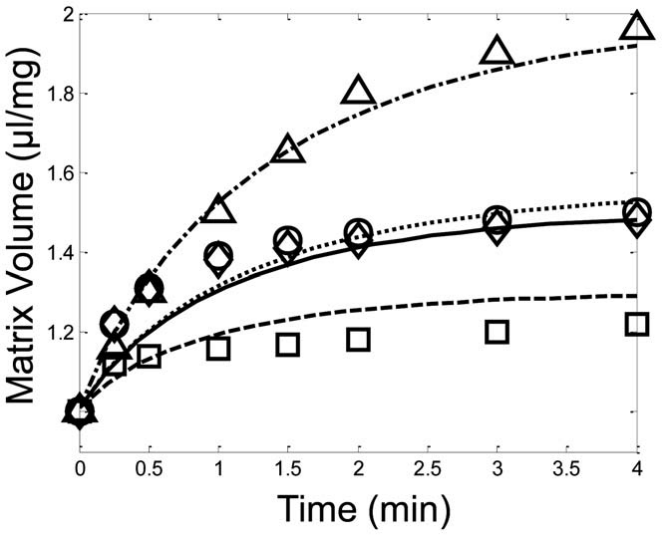
Model simulations (lines) of the matrix volume dynamics under various altered states of the mitochondrial bioenergetics shown in comparison with isolated mitochondria experimental data (symbols) for the conditions outlined in Kowaltowski et al. [11]. Mitochondria were incubated in the assay buffer identified in the Methods section under the Kowaltowski data set description. A small amount of ATP, 200 µM, was included in the assay buffer with either 0.5 µg/mg oligomycin (solid line, circles), 1 mM ADP (dashed line, squares) or 0.5 µg/mg oligomycin+1 mM ADP (dotted line, diamonds). In a separate experiment, no ATP was included in the assay buffer with 0.5 µg/ml oligomycin (dash-dot line, triangles).

The volume dynamics were fitted to the transient mitochondrial matrix swelling data published by Kowalowski et al. [11]. They measured the effect of varying matrix ATP levels on the swelling dynamics in K-salt media with succinate-energized mitochondria. Figure 6 shows that the volume dynamics were captured well by the model. When a small amount of ATP was included in the buffer in the presence of oligomycin, a F_1_F_O_ ATP synthase inhibitor, the mitochondria swelled to approximately 1.5 µL/mg in about four minutes. When the ATP was supplemented with ADP, oxidative phosphorylation was activated which lowered the Δψ and generated matrix ATP. This reduced the electrophoretic potassium uptake and inhibited the mKATP channel, respectively, so that the steady state volume reached a lower value of approximately 1.2 µL/mg. When ATP was not present, all residual matrix ATP was converted to ADP via reverse activation of the F_1_F_O_ ATP synthase. This fully activated the mKATP channel, so that the final steady state volume reached a much higher value of about 2.0 µL/mg.

### Corroborating the Model through Simulation

Model corroboration was necessary to establish confidence in the model resulting from these efforts. Herein, the corroboration considered the robustness of the model to local parameter perturbations, the qualitative agreement of predicted trends with experimental observations, and the ability of the model to reproduce experimental data that was not used in fitting its parameters.

A local sensitivity analysis on the model was performed to determine how robust the model simulations were to local perturbations in the parameter values of all the experiments used in the parameter identification. The absolute-value normalized local sensitivity coefficients (*LSC*) were computed using Equation 2 as defined in the Methods section which considered variations for every state variable dynamics uniformly throughout the simulated experiment duration. The average *LSC* of all 359 parameters was only 7.38×10^−3^ with a variance of 1.18×10^−3^. This implies on average that a perturbation of 1% for a given parameter results in less than a 0.738 +/− 0.118% change in the state dynamics of the model for the experiments considered. Of the 42 adjustable parameters, the average of the absolute-value normalized *LSC* reported in Table 2 was 1.26×10^−3^ with a variance of 3.23×10^−4^. These low sensitivities for the model parameter values did not reveal any inherent problems with the model structure.

The model was also able to reproduce the well known mitochondrial shrinkage/swelling dynamics in the presence of Pi and ADP. Figure 2F shows that as the extra-mitochondrial Pi-titration was increased, mitochondrial matrix water volume increased with the state 3 volume being lower than the state 2 volume. Unfortunately, no volume data for the Bose data set was given; however, the model results do corroborate the qualitative observations presented therein with volume increasing for higher amounts of Pi in the medium.

**Figure 7:**
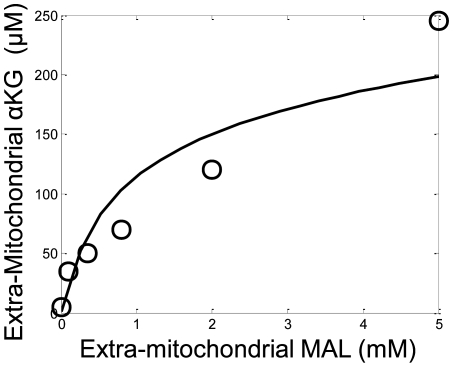
Model predictions (line) of the steady state extra-mitochondrial α-ketoglutarate concentrations during state 2 respiration with various extra-mitochondrial malate concentrations (symbols) is compared to the experimentally reported values outlined in LaNoue et al. [9]. Mitochondria were incubated in the assay buffer identified in the Methods section under the LaNoue data set description. Extra-mitochondrial malate was incrementally increased from 0 to 5 mM in the presence of 2 mM pyruvate. The model was simulated at sufficient times to achieve α-ketoglutarate steady state concentrations.

The model was directly corroborated by predicting the steady state accumulation of extra-mitochondrial α-ketoglutarate during state 2 respiration at various extra-mitochondrial malate concentrations as shown in Figure 7. As the extra-mitochondrial malate concentration was increased, the total amount of α-ketoglutarate generated from the oxidation of pyruvate was also increased and exchanged with the malate in the media via the oxoglutarate-malate exchanger. This exchanger is a critical component of the malate-aspartate shuttle and is particularly important in heart tissue.

**Figure 8:**
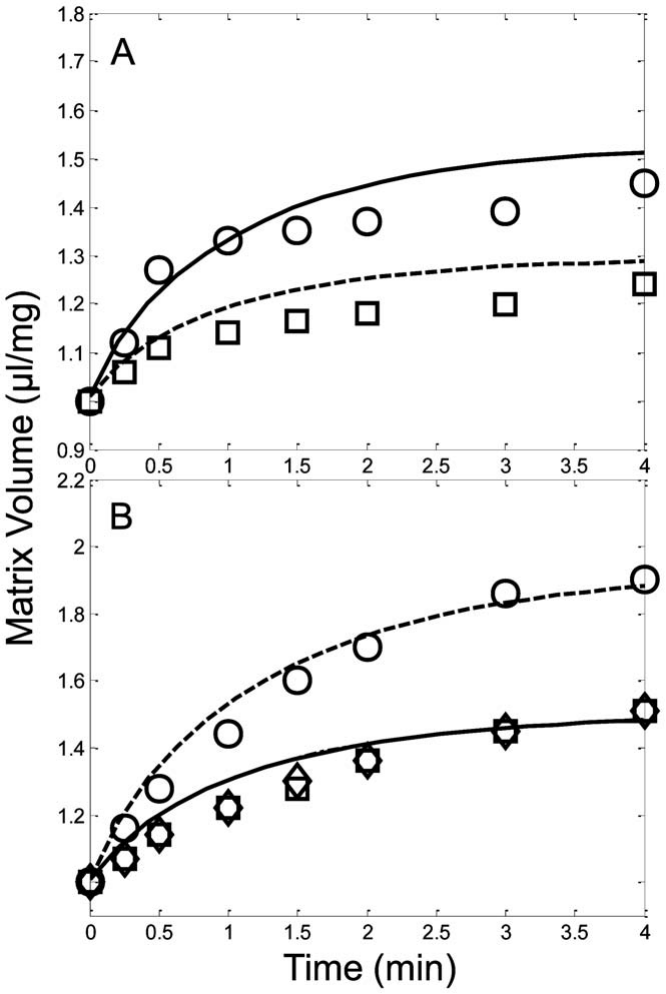
Model predictions (lines) of mitochondrial volume in comparison with isolated mitochondria experimental data (symbols) for the conditions outlined in Kowaltowski et al. [11]. Mitochondria were incubated in the assay buffer identified in the Methods section under the Kowaltowski data set description. A) In each case, 200 µM ATP was included in the assay buffer. Volume dynamics were then measured with either 1 mM ADP and 30 µM diazoxide (solid line, circles) or 1 mM ADP, 30 µM diazoxide and 300 µM 5-hydroxydecanoate (dashed line, sqaures) added to the assay buffer. B) In each case, 0.5 ug/ml oligomycin was included in the assay buffer. Volume dynamics were then measured with either 200 µM ATP (dashed line, circles); 200 µM ATP and 30 µM diazoxide (solid line, square) or 200 µM ATP, 30 µM diazoxide and 300 µM 5-hydroxydecanoate (dash-dot line, diamond) added to the assay buffer (note, the dash-dot line is hidden by the solid line).

The model was again directly corroborated by predicting the experimentally observed mitochondrial volume dynamics after various bioenergetic and/or mKATP interventions. Figure 8 shows that when the mKATP channel was manipulated via normal or pharmacological pathways, the model was capable of predicting the matrix volume changes observed. This highlights the interactions between K^+^-influx via mKATP and mKleak and K^+^-efflux via mKHE and identifies their role in mitochondrial volume regulation. Upon energization, electrophoretic uptake of K-salts increased matrix volume in an osmotic fashion. The first principles representation of mitochondrial volume dynamics was captured well by the model.

**Figure 9:**
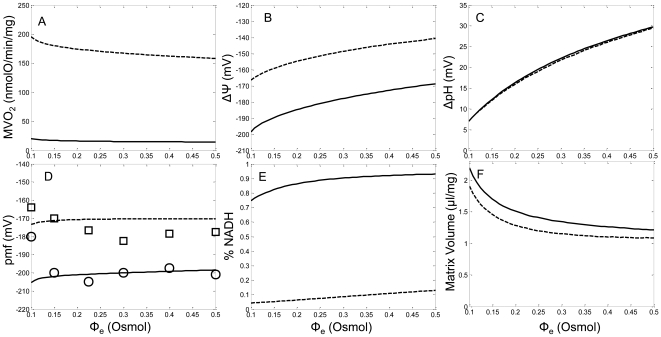
Model predictions (lines) of mitochondrial bioenergetic parameters under differing osmotic KCl medium with isolated mitochondria experimental data (symbols) for the conditions outlined in Devin et al. [12]. Mitochondria were incubated in the assay buffer identified in the Methods section under the Devin data set description. As the osmotic pressure of the KCl medium was adjusted from hypoosmotic to hyperosmotic conditions, A) MVO_2_, B) Δψ, C) ΔpH (defined as pH_mtx_-pH_cyt_), D) proton motive force (Δψ+2.303RT/FΔpH), E) %NADH level and F) matrix volume predicted by the model is presented.

Finally, the model's ability to reproduce the expected trends in bioenergetic variables under varying KCl buffer osmolarity conditions was explored. Devin et al. [12] monitored changes in state 2 and state 3 Δψ, matrix pH, proton motive force, MVO_2_, NADH level and matrix volume as the buffer osmolarity was changed from a hypoosmolar to a hyperosmolar KCl medium using rat liver mitochondria. Although the model presented in this manuscript was optimized to reproduce experimental data primarily from heart tissue, the effect of varying medium osmolarities on key bioenergetic variables was qualitatively reproduced. Only a partial quantitative comparison to this data was possible due to tissue source differences, experimental limitations, and model structure (as further described in the Discussion). Figure 9A shows that the simulated state 2 MVO_2_ matched the experimental trend; however, the model simulated the incorrect trend for state 3 MVO_2_. This is attributed to the tissue source and model structure detailed below in the Discussion. The simulated Δψ trends matched the reported experimental trends (state 2: −140 to −160 mV and state 3: −125 to −135 mV) as shown in Figure 9B. Also, the simulated ΔpH (pH_mtx_-pH_ims_) trends matched the reported experimental trends (state 2: −30 to −45 mV and state 3: −35 to −50 mV) as shown in Figure 9C. Although the simulated Δψs and the ΔpHs are over- and underestimated, respectively, the proton motive force (Δψ + 2.303RT/F ΔpH) matched the experimental data very well as shown in Figure 9D. In other words, as the medium osmolarity varied the total thermodynamic driving force established by the ETS was very similar to that observed experimentally. Devin et al. observed that from hypoosmotic conditions, the NADH levels increased and leveled off as the buffer osmolarity was increased towards hyperosmotic conditions. As shown in Figure 9E, the model was able to reproduce this trend very well. They also reported that in hyperosmotic KCl medium, mitochondrial matrix volume is efficiently regulated, such that the steady state volume is essentially retained, but in hyposomotic KCl medium, the matrix volume dramatically increases relative to isoosomotic conditions [12]. Figure 9F shows that the model volume mechanics adequately reproduced this phenomenon. Overall, the model corroborated the trends of the changes in bioenergetic variables as buffer osmolarity varies.

## Discussion

The model presented in this manuscript is based on previous models [1]–[4] and includes integrated calcium dynamics and a detailed description of the K^+^-cycle and its effect on mitochondrial bioenergetics and matrix volume regulation. Simulations were used to fit 42 adjustable parameters to four independent experimental data sets consisting of 32 data curves of both transient and steady state data. A sensitivity analysis was performed on the model to reveal the most sensitive components of mitochondrial bioenergetics relative to the experimental conditions modeled herein and revealed no inherent model structural problems. Finally, several simulations were performed to corroborate the model.

The mitochondrial volume dynamics and the associated K^+^-cycle appear to play an important role in cellular and mitochondrial bioenergetics [15],[38]. Energy transduction, namely adenine nucleotide outer membrane permeability, is regulated under both physiological and pathophysiological conditions [39]. The IMS volume is partly responsible for this regulation by having a direct effect on the cellular bioenergetics *in vivo* [38]. For example, the adenine nucleotide outer membrane permeability is typically high in freshly isolated mitochondria due to matrix contraction following potassium depletion. During mitochondrial swelling, the increase in matrix volume causes a reciprocal decrease in IMS volume that enables creatine kinase to bind to the voltage-dependent anion channel thus reducing the adenine nucleotide outer membrane permeability. Matrix contraction also occurs *in vivo* during ischemia. This contraction interferes with the regulation of the adenine nucleotide outer membrane permeability, thereby enabling mitochondria to burn up all the cells available ATP and primes the cell for apoptosis before reperfusion. As a natural defense, potassium influx via mKATP increases matrix volume and helps mitigate the detrimental effects of increased adenine nucleotide outer membrane permeability [38]. As a first step to consider these important volume regulatory events, the model incorporates simple volume dynamics based on osmotic pressures generated by the K^+^-cycle and other associated processes. Future work studying this intricate energy transduction mechanism is in the beginning stages of development.

The hypothesized volume-dependent mKHE by Garid [15] was incorporated into the model. This volume dependence is necessary to maintain sufficient potassium efflux at high Δψ during mKATP opening. Without this dependence, the mKHE would be an ineffective volume regulatory mechanism, and the outer membrane would rupture. Specifically, potassium influx induced upon mKATP opening is maintained and essentially constant at a given Δψ because the current cannot sufficiently depolarize the Δψ and because of thermodynamic considerations [33]. Also, model simulations revealed that matrix free Mg^2+^ only decreased from 0.4 mM to approximately 0.15 mM (depending on total amount of Mg-ligands, such as ATP and Pi, present in the matrix and the matrix volume) under the experimental conditions. This decrease is insufficient to serve as the primary volume controller required by the carrier-brake hypothesis. These observations require that mKHE posses some sort of volume dependence enabling an effective volume controlling mechanism. This hypothesis was supported by simulation results presented in Figures 6, 8 and 9F whereby the exquisite control of matrix volume exhibited by mKHE was revealed.

To faithfully reproduce all the data sets, a certain network topology had to be in place and some assumptions about uncertain or unobserved experimental factors and conditions were explicitly constrained. It was found that these network features and experimental assumptions described below were necessary to successfully recreate all the experimentally observed data and trends. The necessity of these realizations contributes to our collective understanding of the mitochondrial bioenergetics.

The intrinsic thermodynamic dissipation of a system can override or mitigate enzymatic regulation [40]. For example, in glutamate/malate energized mitochondria, αKGDH serves as a key regulatory enzyme responsible for maintaining sufficient NADH levels sustaining MVO_2_ rates in state 2 and state 3. This reaction is far from equilibrium making it sensitive to its regulatory mechanisms. In contrast, malate dehydrogenase (MDH) is much closer to equilibrium so its regulatory mechanism less effectively controls the dehydrogenase rate. In state 2, αKGDH is a major enzyme in the pathway responsible for regenerating the matrix ATP that is consumed by the F_1_F_O_ ATP synthase to help the ETS establish a high Δψ. In state 3, this enzyme's activity helps dictate which path the carbon substrates flow through the TCA cycle. The regulation for this enzyme helps enable mitochondria to achieve the steady state NADH levels observed with the Bose Pi-titration data seen in Figure 2B. The relative rates identified from the model simulations (not shown) predicted that the regulation of MDH played less of a role in the steady state NADH Pi-dependent levels than the regulation of αKGDH.

Animal model species specific parameterization may be required for detailed mathematical models of the mitochondrial bioenergetics; however, at this time there is not sufficient data from a single species to fully characterize the dynamics. For example, all the kinetic parameters for the αKGDH expression were found using independent data sets obtained from porcine heart mitochondria as described in Part S3 of the Supplemental Material (Text S1) [18]–[21]. Only 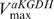, the maximum rate constant, was refit with the fully integrated model simulations that used data from both porcine and rat heart mitochondria. Future compensation for the species specific enzyme kinetic differences between these two isozymes may further improve the fit of the simulated α-ketoglutarate dynamics to the experimental data in Figure 3D.

The tissue type used for the supporting experiments is also important for parameterization of a semi-mechanistic mathematical model. For example, in the model, several exchangers and cotransporters are reported to possess low activity in heart tissue compared with other tissues [41]. Although the definition for low activity was ambiguous; herein to reproduce the experimental data, it was necessary that some of these processes possess unexpectedly elevated activities. For example without sufficiently active glutamate-H cotransport, the model was unable to reproduce the observed aspartate/glutamate dynamics reported by LaNoue et al. [9] in Figure 4. The glutamate-H cotransporter is responsible for the electroneutral transport of the amino acid glutamate and a proton through the inner-mitochondrial membrane down a concentration gradient. This provides a glutamate leak pathway that reduces the matrix aspartate/glutamate pool. Reducing aspartate availability in the matrix prevents the thermodynamically favorable reaction catalyzed by GOT from consuming all of the endogenous aspartate. Alternatively to the proposed elevation in the activity of the cotransporter, it has been argued that there may be two separate aspartate pools [42] in mitochondria. Compartmentalizing the total aspartate pool with slow or volume-dependent transport rates would also enable the model to reproduce the aspartate/glutamate dynamics in the LaNoue data set. As further indirect support for the proposed elevated glutamate-H cotransporter activity, increased glutamate influx helps enable state 2 and state 3 respiration on glutamate and malate for the Bose data set simulations as shown in Figure 2. At this point, neither mechanism has been proven experimentally.

Another exchanger reported to possess low activity in heart tissue is the dicarboxylate carrier [43]. This carrier is responsible for the exchange of primarily Pi, malate and succinate. However, without sufficiently high activity, the succinate-energized mitochondrial matrix volume data reported by Kowaltowski et al. [11] could not be reproduced by the model. Elevated DCC rates were necessary to provide sufficient succinate influx allowing electrophoretic potassium uptake. If the DCC activity was limited to the reported maximum rate [44], the mitochondrial Δψ would not polarize substantially and mitigate the reported potassium-dependent volume increase.

The chosen substrates for the DCC also affected the model simulation capabilities. In the model formulation, only Pi, malate and succinate were assigned as the DCC substrates. Fumarate is also reported to be a substrate for the DCC [45] but was not included in the model due to insufficient data to characterize the kinetics. Including fumarate in the list of the DCC substrates would enable to the model to reproduce the accumulated fumarate data from LaNoue data set (not shown). Also, the omission of fumarate as a substrate for the DCC in addition to the DCC elevated activity contributed to the simulated state 3 malate net oxidation present in Figure 3F.

An additional tissue source related phenomena uncovered during model development was the choice of the calcium dissociation constant for the CaUNI. During model development, a single calcium dissociation constant was chosen to model the CaUNI kinetics from rat heart and liver tissue. This was achieved by considering the competitive nature of magnesium inhibition with respect to calcium binding. It is plausible that the major difference between liver and heart CaUNI kinetics is due to different expression levels versus different calcium binding affinities (as may be plausible between different species); however, more research concerning this matter needs to be done.

Mitochondria from specific tissue types are phenotypically different and contain various amounts of electron transport proteins, matrix proteins and lipid types optimized to support their designated function. Specifically, heart mitochondria possess much higher electron transport activity relative to liver mitochondria [46]. Therefore, we partially attribute the discrepancies between the simulated and reported values for the MVO_2_, Δψ and ΔpH, and under varying KCl buffer osmolarity conditions to differences in the tissue source. The experiments outlined in Devin et al. [12] were done using mitochondria isolated from rat liver while the model was primarily developed to fit data obtained from heart tissue. This is exemplified in Figure 9A–C. The model simulated much higher state 3 MVO_2_ rates (200 versus 100 nmolO/min/mg); however, the state 2 MVO_2_ trends (quantitative data not given) matched those reported. The measured liver mitochondria Δψs were several 10 s of mVs lower relative to simulated Δψs and is also partially attributed to the model's overestimation of state 3 Δψ seen in Figure 2C. Although the Δψ and ΔpH energetic variables were quantitatively different, it is interesting to note that the thermodynamic variable (proton motive force) was consistent across the tissue types as would be expected. Moreover, the authors suggested that the measured hypoosmolar Δψ may have been in error due to volume dependent rhodamine 123 accumulation. This accumulation may partially account for the discrepancy between the measured hypoosmotic state 2 proton motive and that simulated. Thus the model predicts that the cardiac mitochondrial bioenergetic response to varying buffer osmolarity under the conditions presented in Devin et al. would produce Δψs in the range of −200 to −140 mV, ΔpH in the range of 7 to 30 mV (0.2 to 0.5 pH units) and MVO_2_ rates in the range of 200 to 15 nmolO/mg/min.

The translation of the biochemical processes to appropriate mathematically descriptive expressions plays a large role in the simulated dynamics. For example, the citrate and isocitrate overestimation by the model as shown in Figure 3C is attributed to the simple passive exchange process used to model the tricarboxylate carrier (TCC). Replacing this exchange process with a more kinetically descriptive mechanism [47] would help prevent the simulated early accumulation of citrate and isocitrate; however, there is insufficient data to parameterize such a descriptive mechanism. Partially as a function of the simplified description, the activity of the TCC is elevated even though it is reported to possess low activity in heart tissue compared to that in liver tissue [41]. Additionally, the passive TCC exchange process required higher PDH rates in order to sustain sufficient citrate, isocitrate, α-ketoglutarate and succinate levels during both state 2 and especially state 3 respiration seen in Figure 3. Together, the TCC and DCC modeling approximations contribute to the simulated higher malate net oxidation rates than those observed in the LaNoue data set (Figure 3F) since the TCC also exchanges malate across the inner-mitochondrial membrane [47].

During the model development, it is important to consider any artifacts in the experimental data that may have been inadvertently generated during the mitochondrial isolation. For example, the extraction medium must contain Pi to achieve stable, well-coupled mitochondria [41]. To enable the Bose study with Pi-depleted mitochondria, a special isolation procedure was necessary. This Pi-depletion method may have dramatically changed some of the mitochondrial protein phosphorylation states thus having an unknown regulatory effect [48]. Within the short time scales of the Bose experiments, the slower phosphorylation and dephosphorylation events may not have sufficiently occurred upon the Pi-titration. Specifically, the Pi-depletion method may have altered the proton permeability via cation/proton exchange and/or anion/proton cotransport activity. In these experiments, the buffer pH was fixed at 7.1. With the mKHE rate expression identical to that used with the other data sets, the simulated Δψ is underestimated while the ΔpH is overestimated, but the total energetic contribution from both Δψ and ΔpH was nearly identical to that reported. This compensation results from their thermodynamic equivalence with some important kinetic differences. To achieve the low ΔpH values reported experimentally, the volume-dependent mKHE expression had to be replaced with a high activity K^+^/H^+^ exchanger. It is postulated that the Pi-depletion dramatically altered the proton permeability. This is manifested in the model by essentially creating a high activity rapid equilibrium exchanger mechanism that is not supported by the volume-dependent mKHE expression. However, this high activity K^+^/H^+^ exchanger is not compatible with the volume dynamics presented in the Kowaltowski data set, so it is only used for the Bose data set simulations since their isolation procedure was done without Pi. Although we chose to address this discrepancy using a high K^+^/H^+^ exchanger, there are several alternative mechanisms that could also potentially describe or contribute to the ΔpH discrepancy. For example, the mNHE could be responsible for converting more of the ΔpH into the Δψ than the model predicted. It is possible the mNHE activity is underestimated; however, the models ability to match the reported steady state matrix free calcium concentration at varied buffer sodium concentrations shown in Figure 5 indicate this is not the case. Alternatively, the differences between porcine and rat heart mitochondria may be responsible for the dramatic change in proton permeability. The Bose data set was obtained using porcine heart mitochondria, while rat heart mitochondria were used for all the other datasets. It is not likely that the proton permeability associated with these two species is significantly different. Conversely, the volume-dependent mKHE expression may not sufficiently capture the phenomena. The model fits and corroboration simulations presented in Figure 6 and Figure 8, respectively, refute this conclusion. From this discussion, it is evident that more experimental data measuring ΔpH and Δψ under various conditions is needed to reduce this uncertainty.

To simulate the precise experimental conditions during model development, a few explicit assumptions were necessary. For example, the LaNoue data set reported using 3–4 mg of mitochondrial protein; however, as little as a 25% change in mitochondrial load can dramatically alter the total substrate consumption and product accumulation during high MVO_2_ rates. Hence for these simulations, the conditions needed to be known with more certainty. The reported malate concentration was used to estimate the mitochondrial load. The initial malate content in state 2 and state 3 experiments with pyruvate was reported to be 1430 nmol/mg. This required that the mitochondrial load be 3.5 mg/mL using the stated 5 mM malate concentration. To compute this estimate, it was necessary to also consider the pyruvate concentration. The pyruvate concentration was 2 mM; however after 8 minutes of state 3 respiration, the pyruvate utilization was 848 nmol/mg. Considering the 1 mL chamber volume, this implied that the total initial pyruvate concentration was 2.5–3.4 mM and not 2 mM. In an attempt to address these potential data inconsistencies, the model simulations were fit to the data using a mitochondrial load of 3.5 mg/mL and the reported state 3 pyruvate utilizations were subsequently adjusted to be consistent with an initial pyruvate concentration of 2 mM.

All mathematical models are abstractions of the underlying process; the level of detail included in the model is dependent upon the application. This is particularly true for the calcium dynamics associated with mitochondrial bioenergetics. There are known omissions in this and previous models of these calcium dynamics. The mitochondrial Na^+^/Ca^2+^ dynamics were simulated using a simplified Na^+^/Ca^2+^ cycling mechanisms with only the CaUNI, mNCE and mNHE processes represented. This simplification prohibited a mechanistic representation of the actual physiological event. For example, the omission of the rapid mode of calcium uptake (RAM) [49] process necessitated a high CaUNI influx to reach the steady state calcium measurements (from the Wan data set) within a few minutes of extra-mitochondrial calcium addition. (Note, with this higher calcium influx rates, the model still predicts the Na^+^/Ca^2+^ cycle consumes less than 1% of the proton electrochemical gradient established by the ETS.) Also, the Na^+^-independent calcium efflux mechanism is not included in the model formulation since the underlying process is uncertain (electroneutral or electrogenic [50]–[52]) even though it is estimated to contribute up to 33% of total calcium efflux in heart tissue [53]. This omitted calcium efflux mechanism is insensitive to magnesium and prevented adequate fits to the Mg^2+^-titration data presented in Wan et al.; however, Mg^2+^-dependence of the CaUNI and mNCE did enable the simulations to reproduce the reported steady state matrix calcium levels within a few 100 nM (not shown). An explicit, detailed study of the Na^+^/Ca^2+^ dynamics should be modeled under various experimental conditions to fully characterize and understand this process at a more mechanistic level.

In summary, the model presented in this manuscript proposes an extended mitochondrial bioenergetics model targeted at the cardiac myocyte with the parameters estimated using four independent data sets consisting of 32 data curves. It was capable of fitting the data with good fidelity, had relatively little parameter sensitivity relative to the experimental conditions modeled herein and was capable of adequately modeling metabolic trends during the various conditions simulated. The resulting model simulations reproduce observed mitochondrial volume dynamics lending additional support to the current prevailing theory of mitochondrial volume regulation through the mKHE volume-sensitive exchange rate. The model builds upon previous successes and helps refine and establish a global model framework relating to mitochondrial bioenergetics. During the model development, a certain network topology had to be in place and some assumptions about uncertain or unobserved experimental factors and conditions were explicitly constrained to reproduce all the data sets. Specifically, the effect of intrinsic thermodynamic dissipation of the system on enzymatic regulation, importance of animal species and tissue sources differences, mechanistic detail of the model and potential impact of the experimental environment all help constrain the model formulation contributing to the construction of a successful and physiologically faithful model.

The model can serve as a foundation for further extension and refinement efforts. Future work may consider more detailed and mechanistic mathematical abstractions for the ETS and TCC, Ca^2+^ dynamics including the RAM and Na^+^-independent Ca^2+^ efflux pathways, catabolic (i.e., glutamate dehydrogenase) and anabolic (i.e., pyruvate carboxyalse) reactions and β-oxidation pathways enabling integration into whole cell models of cardiac myocytes. Each of these additions will require additional experimental data taken under well controlled and documented conditions in order to be properly reproduced by the model proposed in this manuscript. For example, changing the passive exchange mechanism of the TCC to a more mechanistic, saturable exchange process should enable better fits to the LaNoue data set. This change would keep matrix citrate and isocitrate at sufficient levels to maintain α-ketoglutarate and succinate concentrations experimentally observed allowing fumarate to be included in the list of DCC substrates. With fumarate being removed from the matrix by the DCC, SDH inhibition would be mitigated and the lower branch of the TCA cycle would accelerate and prevent net oxidation of malate observed in the LaNoue experimental data set. Additionally, reproducing the respiratory control ratios as done *in silico* by Korzeneiwski and Mazat [54] using the experimentally measured respiratory control ratios measure by Rossignol et al. [55] would help constrain, define and corroborate the mathematical abstractions for the ETS, F_1_F_O_, ANT and PYRH mechanisms. Experiment design with this model could further reduce parameter uncertainties and help test alternative hypotheses including some postulates made in the Discussion. Although much work is ahead, we feel that this model takes a step towards a more complete physiologically faithful mitochondrial bioenergetics model.

## Methods

### Numerical Solutions

The DAEs describing the model were numerically integrated using MATLAB® (2008b) and the stiff ode solver ode15s (10^−3^ relative tolerance and 10^−9^ absolute tolerance for matrix and IMS state variables and 10^−6^ for all others). To increase computational efficiency, vectorized functions were used during model development in the MATLAB® environment. Parameter optimizations and sensitivity analyses were done on a cluster of four 8-core Intel Xeon 3.4 GHz CPUs each with 16 GB of memory and running the Windows 2003 server platform using the Parallel Computing Toolbox. The results obtained were displayed using MATLAB®.

### Objective Function

The objective function used for parameterization of the model is defined as
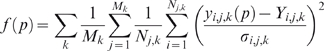
(1)where *f* is the objective function evaluated at a given parameter point *p*, *y_i,j,k_* is the model output, either a state variable or computed rate, corresponding to the *i*th experimental data point in the *j*th experimental data curve for the *k*th data set evaluated at the parameter point *p*, Y*_i,j,k_* is the *i*th experimental data point in the *j*th experimental data curve for the *k*th data set, *σ_i,j,k_* is the standard deviation for the *i*th experimental data point in the *j*th experimental data curve for the *k*th data set, N*_j,k_* is the number of data points in the *j*th experimental data curve for the *k*th data set and *M_k_* is the number of data curves for the *k*th data set. When no statistical data were given with the experimental data, a 5–10% relative error was assumed.

### Parameter Identification

Fitting such large, non-linear models to data with many unknown parameters and initial conditions requires a robust model structure and many independent data sets to appropriately constrain the parameters. The model presented in this manuscript consists of a total of 359 parameters. These parameters were identified using three methods: i) 262 parameters were fixed according to previously published values in the literature (see Part S3 of the Supplemental Material (Text S1)), ii) 55 parameters were found by minimizing the sum of the squares of the difference between simulated rate expressions and the published data using Equation 1 (see Part S3 of the Supplemental Material (Text S1)), and iii) a custom parallelizable Monte Carlo optimization algorithm based on simulated annealing was used to fit the remaining 42 parameters by minimizing Equation 1 with replicated experiments [8]–[11]. Global candidate points were first identified using the simulated annealing approach and refined with a local search using a gradient-based algorithm (MATLAB®'s *fmincon* function).

### Sensitivity Analysis

The local sensitivity analysis was done using the absolute normalized local sensitivity coefficient (*LSC*) for quantifying how much the model output trajectories for the simulated experimental conditions changed in response to small perturbations about the identified parameter set. This LSC is defined as
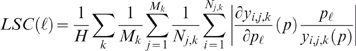
(2)where LSC is the normalized local sensitivity coefficient for the *ℓ*th parameter, *y_i,j,k_* is the model output of the *i*th state at the *j*th time point for the *k*th experimental condition evaluated at the parameter point *p*, *p_ℓ_* is the parameter whose sensitivity is being approximated, N*_j,k_* is the number of states considered for the analysis (note, all 73 states were included in the sensitivity analysis) at the *j*th time point for the *k*th experimental condition, *M_k_* is the time points used in the analysis (note, *M_k_* was fixed and defined as 5 equally spaced time points) for the *k*th experimental condition and *H* is the total number of experimental conditions replicated from [8]–[11]. (Note that although the indices used for the LSC computation are similarly defined, they have different meaning.) Equation 2 was approximated using a centered finite difference using the numerical methods outlined in Conn et al. [56]. To minimize numerical artifacts when computing the LSCs, the model was integrated with stricter tolerances (10^−9^ relative tolerance and 10^−12^ absolute tolerance tolerance for matrix and IMS state variables and 10^−9^ for all others). This unit of measure is used in Table 2 to discern the 1^st^ order, one-at-a-time, effects of small parameter perturbations. A parameter that possesses a large LSC is interpreted as having a substantial influence on the model state trajectories and steady state values.

### Simulating the Experimental Conditions

To simulate the various data sets used to parameterize and corroborate the model, the appropriate experimental conditions were taken into consideration. The temperature at which the experimental data were obtained, the mitochondrial loads applied in each experiment, the initial state of the mitochondria in the experimental system and the precise nature of the experimental environment, specifically the buffer composition and osmolarity were considered. These points are discussed below.

#### Temperature dependence

The rates of biochemical reactions can be extremely sensitive to temperature. A temperature induced change in activity can easily result in doubling or even tripling of some enzymatic reactions for a temperature difference of only 10°C. Since the experiments used to parameterize the model were done at different temperatures, the rates were adjusted according to a standard Q_10_ value of 2.25 or based specifically on the enzyme's activation energy and the Arrhenius rate law. The Arrhenius rate law temperature correction is only truly valid for reactions whose substrates are in rapid equilibrium with their respective substrate-enzyme complexes, and the catalysis step is rate limiting [57]. This becomes significant when dealing with convex Arrhenius plots. The source of convexity is controversial and usually involves different active substrate-enzyme isoforms at different temperatures. We assumed that even though some of the biochemical reactions in the model were not reactions whose substrate-enzyme species were in rapid equilibrium, we could still obtain a reasonable approximation of the temperature correction. In the case of an enzymatic reaction displaying a convex Arrhenius plot, only the linear region in the temperature range of 4–37°C was used to adjust the rates. Table S1.3 in the Supplemental Material (Text S1) shows the compiled list of activation energies used in this model study.

#### Model initialization

It is especially important to use the appropriate initial conditions by either including them in the fitting procedure as variables or conditioning the model by simulation to set common initial operating conditions. We chose the latter and standardized the initial conditions by simulating the model for sufficiently long times with the experimental conditions outlined in each paper. For example, the Bose data set was derived from de-energized, equilibrated mitochondria, the LaNoue data set was derived from de-energized, non-equilibrated mitochondria, the Wan data set was derived from energized, equilibrated mitochondria, the Kowaltowski data set was derived from de-energized, non-equilibrated mitochondria and the Devin data set was derived from energized, equilibrated mitochondria. Once the model was simulated to its fully oxidized state, appropriate modifications to state variables were performed to mimic the experimental conditions as described in each paper used for parameter estimation.

#### Mitochondrial loads

Each experiment used different mitochondrial loads; therefore, for each data set, the buffer water volume relative to the mitochondrial protein content was varied. This is important because the higher the mitochondrial load, the higher the absolute rate of consumption for carbon substrates and oxygen making time series data essential for parameterization purposes. The Bose data set experiments were performed at a mitochondrial load of 1 nmolCyta/mL, or approximately 1 mg mitochondria/mL [58]–[59]. The LaNoue data set experiments used a much larger mitochondrial load of 3–4 mg mitochondria/mL. The reported 3–4 mg mitochondria/mL for the simulations was averaged (as mentioned in the Discussion). The Wan data set experiments were performed at 2 mg mitochondria/mL. The Kowaltowski data set experiments used a mitochondrial load of 0.1 mg mitochondria/mL. The Devin data set experiments reported using 1 mg/mL; however, for the experimental conditions imposed, the liver mitochondria maintained relatively low MVO_2_ rates (approximately 100 nmol O/min/mg) for the duration of the 10 minute experiment. For identical conditions, the model simulates MVO_2_ rates greater than three times this value; therefore, in order to maintain pseudo-steady state conditions for 10 minutes, the mitochondrial load had to be adjusted to 0.1 mg/mL. This prevented the model from simulating considerable ADP consumption rates in order to maintain steady MVO_2_ rates for 10 minutes.

#### Bose experimental conditions

Before simulating the experimental conditions (T = 37°C) in the Bose data set, the model had to be initialized to recreate the Pi-depleted state of the mitochondria. This was done by simulating the model to its fully oxidative state in the presence of ATP followed by a quick Pi-depletion step. Unfortunately, the exact details concerning the Pi-depletion protocol could not be found. For parameter estimation, the basic buffer composition consisted of 125 mM KCl, 15 mM NaCl, 20 mM K-Hepes, 1 mM KEGTA, 1 mM K_2_EDTA, 5 mM MgCl, 4 µM TPP^+^ at pH 7.1. The authors stated that the free [Ca^2+^] was generally held between 500 and 600 nM using CaCl_2_. For simulation purposes, the free [Ca^2+^] was fixed at 550 nM. To initiate respiration, 5 mM glutamate/malate was added to the buffer preceding the Pi-titrations for the simulations. For state 3 respiration, 1.3 mM ADP was added to the medium after the glutamate/malate and Pi additions. For each Pi-titration, the free [K^+^], [Na^+^] and [Mg^2+^] were calculated based on the dissociation constants defined in the Supplemental Material (Text S1). After glutamate/malate was added to the buffer, the model simulated the 60 second experiment reaching pseudo-steady state. Next, Pi was added to the buffer and the model was simulated for the next 60 second experiment reaching another pseudo-steady state. To conclude the experiment, ADP was added to the buffer and the model was simulated for the 30 second experiment reaching its final pseudo-steady state.

#### LaNoue experimental conditions

For the LaNoue data set simulations (T = 28°C), the model was first initialized to achieve the fully oxidized state. The basic buffer composition used for respiratory state initiation consisted of 150 mM KCl, 20 mM Tris-Cl, 20 mM KPi, 5 mM MgCl_2_ and 30 mM glucose at pH 7.2. The mitochondria were preincubated in the basic buffer composition for 30 seconds before addition of substrates. For state 2, either 2 mM pyruvate and 5 mM malate or 1 mM pyruvate was added to the basic buffer composition, and the model was simulated for the specified experimental time. (Note that in the original reference, LaNoue et al. define state 4 as state 2 is defined in this manuscript. They used the previous state nomenclature defined by Chance and Williams [60]. The definitions used here are consistent with those defined by Nicholls and Ferguson [61]). State 3 was simulated similarly, except for the addition of 0.5 mM ADP. With the addition of ADP, the free [K^+^], [Na^+^] and [Mg^2+^] were recalculated based on the dissociation constants defined in the Supplemental Material (Text S1). A hexokinase trap was used to regenerate ADP in the buffer. The simulated time was identical to that reported in the experimental procedure.

#### Wan experimental conditions

The Wan Data set simulations (T = 28°C) also required model initializations from a fully oxidized state. The buffer composition consisted of 130 mM KCl, 20 mM HEPES, 5 mM MgCl_2_, 5 mM ATP, 5 mM KH_2_PO_4_, 5 mM NaCl and 1 mM EGTA at pH 7.0. For the Na^+^- and Mg^2+^-titrations, the free [K^+^], [Na^+^] and [Mg^2+^] were recalculated at each data point based on the dissociation constants defined in the Supplemental Material (Text S1). ATP was included to energize the mitochondria providing an electrophoretic driving force for calcium uptake. The model simulated a 2 minute experiment to ensure steady state and closely matches the time observed in the original experimental procedure.

#### Kowaltowski experimental conditions

The Kowaltowski data set simulations (T = 28°C) were simulated from a fully oxidative and pre-osmotic equilibration state. The basic buffer composition used for respiratory state initiation consisted of 135 mM KCl, 5 mM succinate, 2.5 mM Pi, 100 µM EGTA, 0.5 mM MgCl_2_ at pH 7.2. They applied various bioenergetic pharmaceutical interventions and measured the matrix swelling dynamics. To block the F_1_F_O_ ATP synthase, 0.5 µg/ml oligomycin was used. To simulate this condition, the parameter defining the enzyme activity, 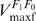, was set to zero. When 30 µM diazoxide, a mKATP channel opener, was used, the endogenous inhibition of the mKATP channel was set to zero. When 300 µM 5-hydroxydecanoate, a mKATP channel closer, was used, the parameter defining the channel conductance, 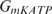, was set to zero. The simulated times are identical to the times reported in the original experimental procedure.

#### Devin experimental conditions

For the Devin data set simulations (T = 26°C), the model was first initialized to achieve the fully oxidized state. The basic buffer composition used for respiratory state initiation consisted of 5 µM TPMP^+^, 5 µM DMO, 5 µM manitol, 20 mM Tris-HCl, 1 mM EGTA, 6 mM Tris-glutamate, 6 mM Tris-malate, 5 mM Tris-Pi at pH 7.2 with varying amounts of KCl used to adjust the osmolarity. The buffer [K^+^] was approximated by setting it equal to half the reported osmolarity. Since no divalent cation was present in the medium, the adenylate kinase reaction was turned off. The model was simulated under state 2 conditions until steady state was reached (a simulated time of 5 minutes) under the varying osmotic conditions. Then, at each osmotic condition, 1 mM of ADP was added to the buffer and the model was simulated to a pseudo-steady state reproducing the reported 10 minute experiments.

## Supporting Information

Text S1 The supplemental material consists of three parts. Part S1 lists the state variables comprising the model, updated Gibbs free energy of formation values, additional and revised dissociation constants, temperature correction method, and general model parameters. Part S2 introduces of the set of 60 non-linear ODEs, five algebraic conservation expressions (for ATP, GTP, NADH, UQH_2_ and c^2+^), five matrix cation ODEs (for H^+^, K^+^, Na^+^, Mg^2+^ and Ca^2+^) and the algebraic expressions for computing matrix and intermembrane space (IMS) water volumes and matrix Cl^−^ is presented. Part S3 discusses the model rate equation derivations and provides all the associated parameter definitions and values.
(2.73 MB DOC)
