# Supplementary material for: Modeling Mitochondrial Bioenergetics with Integrated Volume Dynamics
Source: PLoS Comput Biol. 2010 Jan 1;6(1):e1000632. doi: 10.1371/journal.pcbi.1000632 (PMC2793388; doi:10.1371/journal.pcbi.1000632)
Supplement: Text S1 — The supplemental material consists of three parts. Part S1 lists the state variables comprising the model, updated Gibbs free energy of formation values, additional and revised dissociation constants, temperature correction method, and general model parameters. Part S2 introduces of the set of 60 non-linear ODEs, five algebraic conservation expressions (for ATP, GTP, NADH, UQH2 and c2+), five matrix cation ODEs (for H+, K+, Na+, Mg2+ and Ca2+) and the algebraic expressions for computing matrix and intermembrane space (IMS) water volumes and matrix Cl− is presented. Part S3 discusses the model rate equation derivations and provides all the associated parameter definitions and values. (2.73 MB DOC) [file pcbi.1000632.s001.doc]

**Supplemental Material**

Introduction

Our model builds upon previous published models [1-4] and is more of an extension of the Wu et al. model. We clearly outline where our model is identical and where it diverges from the published models. The supplemental material consists of three parts.

Part S1 lists the state variables comprising the model, updated Gibbs free energy of formation values, additional and revised dissociation constants, temperature correction method, and general model parameters. Note that the units of the solute state variables are in nmol/mg of mitochondrial protein while they are converted to M units when used in the rate expressions. To convert to appropriate concentration units to use in the rate expressions, simply divide the state variable concentration (nmol/mg mitochondrial protein) with respective region’s water volume space (nl/mg mitochondrial protein). We will use nmol/mg and nmol/mg mitochondrial protein synonymously herein.

Part S2 introduces of the set of 60 non-linear ODEs, five algebraic conservation expressions (for ATP, GTP, NADH, UQH_2_ and c^2+^), five matrix cation ODEs (for H^+^, K^+^, Na^+^, Mg^2+^ and Ca^2+^) and the algebraic expressions for computing matrix and intermembrane space (IMS) water volumes and matrix Cl^-^ is presented.

Part S3 discusses the model rate equation derivations and provides all the associated parameter definitions and values. When the rate expressions are identical to a previously published expression, we indicate the source and provide the rate equations and parameters values for completeness. Note that some of the model parameters have changed from their originally published values. In those cases, we give justification for our changes.

**Part S1 - Model State Variables and General Parameters**

S1A - Model State Variables

The model is a 73 state system of DAEs that consists of 65 non-linear ODEs to simulate mitochondrial bioenergetics; five algebraic conservation expressions to compute matrix ATP, GTP, NADH, UQH_2_ and c^2+^; one algebraic expression to compute matrix water volume; one algebraic expression to compute IMS water volume and one algebraic expression to compute matrix Cl^-^. The state variables, their definitions and units are provided in Table S1.1.

**Table S1.1. Model State Variables**

| State Variable | Definition | Units |
| --- | --- | --- |
| *ΔΨ* | Mitochondrial membrane potential | mV |
| *Mitochondrial Volume State Variables* | | |
| *V_mtx_* | Matrix water volume | nL/mg |
| *V_ims_* | Innermembrane space water volume | nL/mg |
| *Mitochondrial Matrix Solute State Variables* | | |
| *[H^+^]_mtx_* | Matrix free proton content | nmol/mg |
| *[K^+^]_mtx_* | Matrix free potassium content | nmol/mg |
| *[Na^+^]_mtx_* | Matrix free sodium content | nmol/mg |
| *[Mg^2+^]_mtx_* | Matrix free magnesium content | nmol/mg |
| *[Ca^2+^]_mtx_* | Matrix free calcium content | nmol/mg |
| *[Cl^-^]_mtx_* | Matrix free chloride content | nmol/mg |
| *[ATP]_mtx_* | Total matrix ATP content | nmol/mg |
| *[ADP]_mtx_* | Total matrix ADP content | nmol/mg |
| *[GTP]_mtx_* | Total matrix GTP content | nmol/mg |
| *[GDP]_mtx_* | Total matrix GDP content | nmol/mg |
| *[Pi]_mtx_* | Total matrix Pi content | nmol/mg |
| *[NADH]_mtx_* | Total matrix NADH content | nmol/mg |
| *[NAD]_mtx_* | Total matrix NAD content | nmol/mg |
| *[UQH_2_]_mtx_* | Total mitochondrial ubiquinol content | nmol/mg |
| *[UQ]_mtx_* | Total mitochondrial ubiquinone content | nmol/mg |
| *[PYR]_mtx_* | Total matrix pyruvate content | nmol/mg |
| *[AcCoA]_mtx_* | Total matrix acetyl-CoA content | nmol/mg |
| *[CIT]_mtx_* | Total matrix citrate content | nmol/mg |
| *[ISOC]_mtx_* | Total matrix isocitrate content | nmol/mg |
| *[αKG]_mtx_* | Total matrix α-ketoglutarate content | nmol/mg |
| *[SCoA]_mtx_* | Total matrix succinyl-CoA content | nmol/mg |
| *[CoASH]_mtx_* | Total matrix CoA content | nmol/mg |
| *[SUC]_mtx_* | Total matrix succinate content | nmol/mg |
| *[FUM]_mtx_* | Total matrix fumarate content | nmol/mg |
| *[MAL]_mtx_* | Total matrix malate content | nmol/mg |
| *[OAA]_mtx_* | Total matrix oxaloacetate content | nmol/mg |
| *[GLU]_mtx_* | Total matrix glutamate content | nmol/mg |
| *[ASP]_mtx_* | Total matrix aspartate content | nmol/mg |
| *[CO_2_,_tot_]_mtx_* | Total CO_2_ matrix content | nmol/mg |
| *[O_2_]_mtx_* | Total O_2_ matrix content | nmol/mg |
| *Intermembrane Space (IMS) Solute State Variables* | | |
| *[H^+^]_ims_* | IMS free proton content | nmol/mg |
| *[K^+^]_ims_* | IMS free potassium content | nmol/mg |
| *[Na^+^]_ims_* | IMS free sodium content | nmol/mg |
| *[Mg^2+^]_ims_* | IMS free magnesium content | nmol/mg |
| *[Ca^2+^]_ims_* | IMS free calcium content | nmol/mg |
| *[ATP]_ims_* | Total IMS ATP content | nmol/mg |
| *[ADP]_ims_* | Total IMS ADP content | nmol/mg |
| *[AMP]_ims_* | Total IMS AMP content | nmol/mg |
| *[Pi]_ims_* | Total IMS Pi content | nmol/mg |
| *[c^2+^]_ims_* | Total IMS cytochrome c^2+^ content | nmol/mg |
| *[c^3+^]_ims_* | Total IMS cytochrome c^3+^ content | nmol/mg |
| *[PYR]_ims_* | Total IMS pyruvate content | nmol/mg |
| *[CIT]_ims_* | Total IMS citrate content | nmol/mg |
| *[ISOC]_ims_* | Total IMS isocitrate content | nmol/mg |
| *[αKG]_ims_* | Total IMS α-ketoglutarate content | nmol/mg |
| *[SUC]_ims_* | Total IMS succinate content | nmol/mg |
| *[FUM]_ims_* | Total IMS fumarate content | nmol/mg |
| *[MAL]_ims_* | Total IMS malate content | nmol/mg |
| *[GLU]_ims_* | Total IMS glutamate content | nmol/mg |
| *[ASP]_ims_* | Total IMS aspartate content | nmol/mg |
| *Mitochondrial Buffer / Cytosolic Solute State Variables* | | |
| *[H^+^]_cyt_* | Extra-mitochondrial free proton content | nmol/mg |
| *[K^+^]_cyt_* | Extra-mitochondrial free potassium content | nmol/mg |
| *[Na^+^]_cyt_* | Extra-mitochondrial free sodium content | nmol/mg |
| *[Mg^2+^]_cyt_* | Extra-mitochondrial free magnesium content | nmol/mg |
| *[Ca^2+^]_cyt_* | Extra-mitochondrial free calcium content | nmol/mg |
| *[ATP]_cyt_* | Total extra-mitochondrial ATP content | nmol/mg |
| *[ADP]_cyt_* | Total extra-mitochondrial ADP content | nmol/mg |
| *[AMP]_cyt_* | Total extra-mitochondrial AMP content | nmol/mg |
| *[Pi]_cyt_* | Total extra-mitochondrial Pi content | nmol/mg |
| *[PYR]_cyt_* | Total extra-mitochondrial pyruvate content | nmol/mg |
| *[CIT]_cyt_* | Total extra-mitochondrial citrate content | nmol/mg |
| *[ISOC]_cyt_* | Total extra-mitochondrial isocitrate content | nmol/mg |
| *[αKG]_cyt_* | Total extra-mitochondrial α-ketoglutarate content | nmol/mg |
| *[SUC]_cyt_* | Total extra-mitochondrial succinate content | nmol/mg |
| *[FUM]_cyt_* | Total extra-mitochondrial fumarate content | nmol/mg |
| *[MAL]_cyt_* | Total extra-mitochondrial malate content | nmol/mg |
| *[GLU]_cyt_* | Total extra-mitochondrial glutamate content | nmol/mg |
| *[ASP]_cyt_* | Total extra-mitochondrial aspartate content | nmol/mg |
| *[GLC]_cyt_* | Total extra-mitochondrial glucose content | nmol/mg |
| *[G6P]_cyt_* | Total extra-mitochondrial glucose-6-phosphate content | nmol/mg |

Note that concentrations are normalized to the mitochondrial protein mass. Oxygen and carbon dioxide concentrations were fixed at 65 μM and 21.4 mM, respectively, as in the Wu et al. model.

To convert the concentrations of the solute state variables to M units, divide the matrix, IMS or extra-mitochondrial solute state variable by the respective region’s water volume per mg value. For example, with the appropriate osmotic balance, the isolated mitochondrial matrix volume is approximately 1 µL/mg mitochondrial protein; therefore, a matrix content of 3.98x10^-5^ nmol/mg H^+^ is 3.98x10^-8^ nmol/nL H^+^ (or equivalently, pH 7.4). By normalizing all the concentrations to the mitochondrial protein content, we were able to integrate volume dynamics with as little ambiguity as possible.

S1B - Gibbs Free Energy of Formation

The Gibbs free energy of formation values are from Wu et al.’s original model (Table A3 in the supplemental material of [1]) with the exception of the ubiquinol formation energy. This was adjusted from its original value of -23.30 to -32.05 kJ/mol at T = 298.15 K and I = 0.17 M to account for the reported ubiquinol/ubiquinone midpoint potential of approximately 90 mV [5-6]. Although the Gibbs free energy of formation is temperature dependent, there was insufficient enthalpy data for all the model reactants to properly compensate for temperature changes. This is addressed in Part S1D.

S1C - Dissociation Constants

There is still some uncertainty in several of the measured dissociation constants used in models like the one proposed in this manuscript pertaining to protonation and metal-ligand dissociation constants. In order to minimize this uncertainty, special care was taken when choosing the dissociation constants used in this model. The background electrolyte, temperature and ionic strength are all important factors necessary to take into account when choosing appropriate values of dissociation constants. Most were taken from Wu et al. (Table A3 in the supplemental material of [1]) who used the NIST Standard Reference Database 46. This list from Wu et al. was revised using Smith et al.’s critical review for the adenine nucleotide series [7] along with other revisions detailed in Table S1.2.

**Table S1.2.** Additional and Revised Dissociation Constants at 25 ºC

| Parameter | Definition | Value | Reference |
| --- | --- | --- | --- |
| $K_{ATP}^{H}$ | Proton ATP binding constant | 10^-6.61^ M | [7] |
| $K_{ATP}^{Na}$ | Sodium ATP binding constant | 10^-1.13^ M | [7] |
| $K_{ATP}^{K}$ | Potassium ATP binding constant | 10^-0.99^ M | [7] |
| $K_{ATP}^{Mg}$ | Magnesium ATP binding constant | 10^-4.47^ M | [7] |
| $K_{ATP}^{Ca}$ | Calcium ATP binding constant | 10^-4.16^ M | [7] |
| $K_{ADP}^{H}$ | Proton ADP binding constant | 10^-6.47^ M | [7] |
| $K_{ADP}^{Na}$ | Sodium ADP binding constant | 10^-1.04^ M | [7] |
| $K_{ADP}^{K}$ | Potassium ADP binding constant | 10^-0.92^ M | [7] |
| $K_{ADP}^{Mg}$ | Magnesium ADP binding constant | 10^-3.41^ M | [7] |
| $K_{ADP}^{Ca}$ | Calcium ADP binding constant | 10^-3.06^ M | [7] |
| $K_{AMP}^{H}$ | Proton AMP binding constant | 10^-6.27^ M | [7] |
| $K_{AMP}^{Na}$ | Sodium AMP binding constant | 10^-0.86^ M | [7] |
| $K_{AMP}^{K}$ | Potassium AMP binding constant | 10^-0.66^ M | [7] |
| $K_{AMP}^{Mg}$ | Magnesium AMP binding constant | 10^-2.02^ M | [7] |
| $K_{AMP}^{Ca}$ | Calcium AMP binding constant | 10^-1.92^ M | [7] |
| $K_{Pi}^{Na}$ | Sodium Pi binding constant | 10^-0.49^ M | [8] |
| $K_{Pi}^{K}$ | Calcium Pi binding constant | 10^-0.61^ M | [8] |
| $K_{Pi}^{Mg}$ | Sodium Pi binding constant | 10^-3.01^ M | [9] |
| $K_{Pi}^{Ca}$ | Calcium Pi binding constant | 10^-2.63^ M | [9] |
| $K_{CIT}^{H}$ | Proton citrate binding constant | 10^-5.63^ M | [10] |
| $K_{CIT}^{K}$ | Potassium citrate binding constant | 10^-0.34^ M | [10] |
| $K_{CIT}^{Mg}$ | Magnesium citrate binding constant | 10^-3.82^ M | [10] |
| $K_{ISOC}^{H}$ | Proton isocitrate binding constant | 10^-5.62^ M | [11] |
| $K_{ISOC}^{Mg}$ | Magnesium isocitrate binding constant | 10^-2.72^ M | [11] |
| $K_{ISOC}^{Ca}$ | Calcium isocitrate binding constant | 10^-2.47^ M | [11] |
| $K_{EGTA}^{H}$ | 1^st^ proton EGTA binding constant | 10^-9.42^ M | [11] |
| $K_{HEGTA}^{H}$ | 2^nd^ proton EGTA binding constant | 10^-8.83^ M | [11] |
| $K_{EGTA}^{K}$ | Potassium EGTA binding constant | 10^-1.18^ M | [11] |
| $K_{EGTA}^{Mg}$ | Magnesium EGTA binding constant | 10^-5.19^ M | [11] |
| $K_{EGTA}^{Ca}$ | Calcium EGTA binding constant | 10^-10.98^ M | [11] |

Dissociations constants were adjusted to an ionic strength of 0.17 M using the methods outlined in Vinnakota et al. [12]. Temperature corrections were not done because of insufficient enthalpy data. GTP and GDP dissociation constants were set equal to the ATP and ADP dissociation constants, respectively.

S1D - Temperature Correction

The thermodynamics and kinetics of biochemical reactions are dependent on temperature. For the range of temperatures given in the experimental data, the temperature effects for the reaction kinetics are not negligible. Additionally, the Gibb’s free energy of formation and dissociation constants can appreciably change; however, this requires a complete thermodynamic data set to properly adjust these thermodynamic parameters. Unfortunately, due to insufficient enthalpy data, temperature corrections for the Gibb’s free energy of formation and dissociation constants could not be done. As more thermodynamic data becomes available, temperature corrections for these parameters should be performed.

In order to apply the temperature correction for the kinetic parameters, the Arrhenius rate law was used. The Arrhenius equation,

$k_{j}=e^{-E_{act}^{j}/R\left(1/T-1/T_{0}\right)}$, (1.1)

where *k_j_* and $E_{act}^{j}$ is the rate modification value and activation energy for the *j*^th^ reaction rate, respectively, and *T_0_* is the reference temperature (298.15 K) was used to adjust rate parameters. When an appropriate activation energy value for a reaction could not be found, we approximated it by assuming an activation energy value of 61.9 kJ/mol. This is equivalent to a Q_10_ value of 2.25. The Q_10_, or temperature correction coefficient, is another method biochemists use to adjust reaction rates at different temperatures. For most biological reactions, the Q_10_ value varies from 2-3 [13]. The list of activation energies used for each reaction is listed in Table S1.3.

**Table S1.3.** Activation Energies for Model Reactions

| Reaction | Activation Energies (kJ/mol) | Reference |
| --- | --- | --- |
| *Mitochondrial Reactions* |  |  |
| Pyruvate dehydrogenase | 61.9 | a |
| Citrate synthase | 61.5 | [14] |
| Aconitase | 61.9 | a |
| Isocitrate dehydrogenase | 61.9 | a |
| α-Ketoglutarate dehydrogenase | 61.9 | a |
| Succinyl CoA synthase | 61.9 | a |
| Succinate dehydrogenase | 134.0 | [15] |
| Fumarate hydratase | 61.9 | a |
| Malate dehydrogenase | 61.9 | a |
| Nucleoside diphosphokinase | 61.9 | a |
| Glutamate oxaloacetate transaminase | 61.9 | a |
| Complex I | 63.0 | [16] |
| Complex III | 63.0 | [16] |
| Complex IV | 61.9 | a |
| F_1_F_O_ ATP synthase | 61.9 | a |
| Adenylate Kinase | 61.9 | a |
| *Exchangers and Ion Channels* |  |  |
| Glutamate-aspartate exchanger | 63.2 | [17] |
| α-Ketoglutarate-malate exchanger | 61.9 | a |
| Pyruvate-proton cotransporter | 61.9 | a |
| Glutamate-proton cotransporter | 61.9 | a |
| Tricarboxylate Carrier | 61.9 | a |
| Dicarboxylate Carrier | 61.9 | a |
| Adenine nucleotide transporter | 31.8 for T=9-40 ºC | [18] |
| Inorganic phosphate carrier | 53.6 | [19] |
| Proton leak | 61.9 | a |
| Potassium-hydrogen exchanger | 61.9 | a |
| ATP-dependent potassium channel | 61.9 | a |
| Potassium leak | 61.9 | a |
| Calcium uniporter | 61.9 | a |
| Sodium-calcium exchanger | 61.9 | a |
| Sodium-hydrogen exchanger | 61.9 | a |

^a^ Based on a Q_10_ value of 2.25.

S1E – General Model Parameters

The general model parameters are presented in Table S1.4.

**Table S1.4.** General Model Parameters

| Parameter | Definition | Value | Reference |
| --- | --- | --- | --- |
| *R* | Ideal gas constant | 8.314x10^-3^ kJ/K/mol | Physical constant |
| *T* | Temperature | 310.15 K | Physical constant |
| *F* | Faraday’s constant | 96.487x10^-3^ kJ/mV/mol | Physical constant |
| *z_Ca_* | Calcium valence | +2 | Physical constant |
| *z_T_* | ATP valence | -4 | Physical constant |
| *z_D_* | ADP valence | -3 | Physical constant |
| *C_mito_* | Mitochondrial membrane capacitance | 1.45×10^-3^ nmol ion/mg/mV | [20] |
| *N_tot_* | Total NAD content | 3 nmol/mg | [4]^a^ |
| *Q_tot_* | Total ubiquinone content | 5.83 nmol/mg | [21] |
| *C_tot_* | Total cytochrome c content | 0.45 nmol/mg | [22] |
| *A_tot_* | Total adenine content | 7.5 nmol/mg | [23] |
| *G_tot_* | Total guanidine content | 5.0 nmol/mg | [1]^a^ |

^a^ Approximated using the reported concentration in M and a matrix water volume of 1 μl/mg.

**Part S2 - Model Differential-Algebraic Equations**

The nonlinear ODEs comprising the model is shown below in Equations 2.01-2.60. The membrane potential differential equation is presented, followed by the matrix, the IMS and the external buffer/cytosolic solute differential equations. The conservation algebraic equations follow and are shown in Equations 2.61-2.65. Since we account for mitochondrial volume dynamics, the units for the matrix, IMS and cytosolic solute state variables are nmol/mg. The free ion matrix content differential equations are presented in Equation 2.66. The mitochondrial matrix and IMS water volumes and the matrix Cl^-^ content are computed using the algebraic expressions presented at the end of this section shown in Equations 2.114, 2.116 and 2.117.

S2A - Model Differential Equations

Mitochondrial Membrane Potential:

(2.01)

Mitochondrial Matrix:

$\frac{d[ADP]_{mtx}}{dt}=J_{ANT}-J_{F_{1}F_{0}}-J_{NDK}$ (2.02)

$\frac{d[GDP]_{mtx}}{dt}=J_{NDK}-J_{ScoAS}$ (2.03)

$\frac{d[Pi]_{mtx}}{dt}=J_{PIC}+J_{MALPi}+J_{SUCPi}-J_{SCoAS}-J_{F_{1}F_{0}}$ (2.04)

$\frac{d[NAD]_{mtx}}{dt}=J_{C1}-J_{PDH}-J_{IDH}-J_{\alpha KGDH}-J_{MDH}$ (2.05)

$\frac{d[UQ]_{mtx}}{dt}=(2-1)J_{C3}-J_{C1}-J_{SDH}$ (2.06)

$\frac{d[PYR]_{mtx}}{dt}=J_{PYRH}-J_{PDH}$ (2.07)

(2.08)

$\frac{d[CIT]_{mtx}}{dt}=J_{CS}+J_{CITMAL}-J_{ACH}$ (2.09)

$\frac{d[ISOC]_{mtx}}{dt}=J_{ACH}-J_{IDH}$ (2.10)

$\frac{d[\alpha KG]_{mtx}}{dt}=J_{IDH}+J_{OME}-J_{\alpha KGDH}-J_{GOT}$ (2.11)

$\frac{d[SCoA]_{mtx}}{dt}=J_{\alpha KGDH}-J_{SCoAS}$ (2.12)

$\frac{d[CoASH]_{mtx}}{dt}=J_{CS}+J_{SCoAS}-J_{\alpha KGDH}-J_{PDH}$ (2.13)

$\frac{d[SUC]_{mtx}}{dt}=J_{SCoAS}-J_{SDH}-J_{SUCPi}$ (2.14)

$\frac{d[FUM]_{mtx}}{dt}=J_{SDH}-J_{FH}$ (2.15)

$\frac{d[MAL]_{mtx}}{dt}=J_{FH}-J_{MDH}-J_{OME}-J_{MALPi}-J_{CITMAL}$ (2.16)

$\frac{d[OAA]_{mtx}}{dt}=J_{MDH}+J_{GOT}-J_{CS}$ (2.17)

$\frac{d[GLU]_{mtx}}{dt}=J_{GOT}+J_{GLUH}+J_{GAE}$ (2.18)

$\frac{d[ASP]_{mtx}}{dt}=-J_{GAE}-J_{GOT}$ (2.19)

$\frac{d[O_{2}]_{mtx}}{dt}=0$ (2.20)

$\frac{d[CO_{2,tot}]_{mtx}}{dt}=0$ (2.21)

Inner-mitochondrial Membrane Space (IMS):

$\frac{d[H^{+}]_{ims}}{dt}=0$ (2.22)

$\frac{d[K^{+}]_{ims}}{dt}=0$ (2.23)

$\frac{d[Na^{+}]_{ims}}{dt}=0$ (2.24)

$\frac{d[Mg^{2+}]_{ims}}{dt}=0$ (2.25)

$\frac{d[Ca^{2+}]_{ims}}{dt}=0$ (2.26)

$\frac{d[c^{3+}]_{ims}}{dt}=2J_{C4}-2J_{C3}$ (2.27)

$\frac{d[ATP]_{ims}}{dt}=J_{ATP}+J_{imAK}+J_{ANT}$ (2.28)

$\frac{d[ADP]_{ims}}{dt}=J_{ADP}-2J_{imAK}-J_{ANT}$ (2.29)

$\frac{d[AMP]_{ims}}{dt}=J_{AMP}+J_{imAK}$ (2.30)

$\frac{d[Pi]_{ims}}{dt}=J_{Pi}-J_{PIC}-J_{MALPi}-J_{SUCPi}$ (2.31)

$\frac{d[PYR]_{ims}}{dt}=J_{PYR}-J_{PYRH}$ (2.32)

$\frac{d[CIT]_{ims}}{dt}=J_{CIT}-J_{CITMAL}$ (2.33)

$\frac{d[ISOC]_{ims}}{dt}=J_{ISOC}-J_{ISOCMAL}$ (2.34)

$\frac{d[\alpha KG]_{ims}}{dt}=J_{\alpha KG}-J_{OME}$ (2.35)

$\frac{d[SUC]_{ims}}{dt}=J_{SUC}+J_{SUCPi}$ (2.36)

$\frac{d[FUM]_{ims}}{dt}=J_{FUM}$ (2.37)

$\frac{d[MAL]_{ims}}{dt}=J_{MAL}+J_{OME}+J_{MALPi}+J_{CITMAL}$ (2.38)

$\frac{d[GLU]_{ims}}{dt}=J_{GLU}-J_{GLUH}-J_{GAE}$ (2.39)

$\frac{d[ASP]_{ims}}{dt}=J_{ASP}+J_{GAE}$ (2.40)

Mitochondrial Buffer / Cytosolic Space:

$\frac{d[H^{+}]_{cyt}}{dt}=0$ (2.41)

$\frac{d[K^{+}]_{cyt}}{dt}=0$ (2.42)

$\frac{d[Na^{+}]_{cyt}}{dt}=0$ (2.43)

$\frac{d[Mg^{2+}]_{cyt}}{dt}=0$ (2.44)

$\frac{d[Ca^{2+}]_{cyt}}{dt}=0$ (2.45)

$\frac{d[ATP]_{cyt}}{dt}=-J_{ATP}-J_{HK}$ (2.46)

$\frac{d[ADP]_{cyt}}{dt}=-J_{ADP}+J_{HK}$ (2.47)

$\frac{d[AMP]_{cyt}}{dt}=-J_{AMP}$ (2.48)

$\frac{d[Pi]_{cyt}}{dt}=-J_{Pi}$ (2.49)

$\frac{d[PYR]_{cyt}}{dt}=-J_{PYR}$ (2.50)

$\frac{d[CIT]_{cyt}}{dt}=-J_{CIT}$ (2.51)

$\frac{d[ISOC]_{cyt}}{dt}=-J_{ISOC}$ (2.52)

$\frac{d[\alpha KG]_{cyt}}{dt}=-J_{\alpha KG}$ (2.53)

$\frac{d[SUC]_{cyt}}{dt}=-J_{SUC}$ (2.54)

$\frac{d[FUM]_{cyt}}{dt}=-J_{FUM}$ (2.55)

$\frac{d[MAL]_{cyt}}{dt}=-J_{MAL}$ (2.56)

$\frac{d[GLU]_{cyt}}{dt}=-J_{GLU}$ (2.57)

$\frac{d[ASP]_{cyt}}{dt}=-J_{ASP}$ (2.58)

$\frac{d[GLC]_{cyt}}{dt}=-J_{HK}$ (2.59)

$\frac{d[G6P]_{cyt}}{dt}=J_{HK}$ (2.60)

Since the outer-mitochondrial membrane (OMM) is highly permeable to ions, the IMS cations were set equal to the buffer/cytosolic cations. For the experimental conditions employed during model development, fitting and corroboration, the cytosolic cation concentrations were held fixed for the duration of the simulated experiments.

S2B - Matrix Conservation Algebraic Equations

The mitochondrial species for adenine nucleotides, guanidine nucleotides, nitcotiname adenine dinucleotide, ubiquinone and cytochrome c are conserved in the model. The conservation is implemented by using an algebraic expression to govern the conservation. This is done using a mass matrix with the integration algorithm and setting the appropriate rows equal to a zero row vector.

$[ATP]_{mtx}=A_{tot}-[ADP]_{mtx}$ (2.61)

$[GTP]_{mtx}=G_{tot}-[GDP]_{mtx}$ (2.62)

$[NADH]_{mtx}=N_{tot}-[NAD]_{mtx}$ (2.63)

$[UQH_{2}]_{mtx}=Q_{tot}-[UQ]_{mtx}$ (2.64)

$[c^{2+}]_{ims}=C_{tot}-[c^{3+}]_{ims}$ (2.65)

S2C - Matrix Cation Differential Equation Derivations

The cation differential equations are derived using the method outlined by Vinnakota et al. [12]. Due to the large expressions resulting from the derivation, the method used to obtain them is presented versus explicitly showing all the terms that enter the differential equations (each differential equation would take several pages). The equations we used in our model can be obtained by solving the linear system of equations given in Equation 2.66. Note that the units of solute state variables (cations and ligands) are in nmol/mg and need to be converted to M concentration units before evaluating the partial derivative expressions and buffering functions. The resulting matrix cation differential equations are in nmol/mg/min.

The system of equations relating the cation differential equations is

(2.66)

where the diagonal terms are the buffering expressions and defined as

$D_{H}=1+\frac{\partial[H_{bound}]}{\partial[H^{+}]}+\frac{B_{H}}{K_{BH}\left(1+[H^{+}]/K_{BH}\right)^{2}}$, (2.67)

$D_{K}=1+\frac{\partial[K_{bound}]}{\partial[K^{+}]}$, (2.68)

$D_{Na}=1+\frac{\partial[Na_{bound}]}{\partial[Na^{+}]}$, (2.69)

$D_{Mg}=1+\frac{\partial[Mg_{bound}]}{\partial[Mg^{2+}]}+\frac{B_{Mg}}{K_{BMg}\left(1+[Mg^{2+}]/K_{BMg}\right)^{2}}$, (2.70)

and

$D_{Ca}=1+\frac{\partial[Ca_{bound}]}{\partial[Ca^{2+}]}+\frac{B_{Ca}}{K_{BCa}\left(1+[Ca^{2+}]/K_{BCa}\right)^{2}}$. (2.71)

The mitochondrial non-diffusible proton binding sites (*B_H_*) and proton dissociation constant (*K_BH_*) were identical to Wu et al. [1]. Since mitochondrial matrix water volume dynamically changes, the original concentration of non-diffusible proton binding sites had to be converted from 0.02 M to 20 nmol/mg using a matrix water volume of 1 µl/mg. The proton dissociation constant was set 10^-7^ M as in Wu et al [1].

The total matrix magnesium in the model was constant. The concentration of mitochondrial non-diffusible magnesium binding sites (*B_Mg_*) was set to 32 nmol/mg with a magnesium dissociation constant (*K_BMg_*) of 340x10^-6^ M [24].

The mitochondrial non-diffusible calcium binding sites (*B_Ca_*) and calcium dissociation constant (*K_BCa_*) were defined according to Corkey et al. [25]. They found that mitochondria contain approximately 11.9 nmol/mg calcium non-diffusible binding sites with a calcium dissociation constant of 1.66x10^-6^ M.

Assuming higher order cation binding is negligible, the cation partial derivative expressions are defined below where *N_r_* is the number of reactants, *L_i_* is the *i*^th^ ligand, $K_{i}^{j}$is the dissociation constant for the *i*^th^ ligand and *j*^th^ cation couple and *P_i_* is the binding polynomial for the *i*th ligand as originally defined by Alberty [26].

$\frac{\partial[H_{bound}]}{\partial[H^{+}]}=\sum_{i=1}^{Nr} \frac{[L_{i}]\left(1+[K^{+}]/K_{i}^{K}+[Na^{+}]/K_{i}^{Na}+[Mg^{2+}]/K_{i}^{Mg}+[Ca^{2+}]/K_{i}^{Ca}\right)}{K_{i}^{H}P_{i}^{2}}$, (2.72)

$H_{K}=\frac{\partial[H_{bound}]}{\partial[K^{+}]}=-[H^{+}]\sum_{i=1}^{Nr} \frac{[L_{i}]/K_{i}^{H}}{K_{i}^{K}P_{i}^{2}}$, (2.73)

$H_{Na}=\frac{\partial[H_{bound}]}{\partial[Na^{+}]}=-[H^{+}]\sum_{i=1}^{Nr} \frac{[L_{i}]/K_{i}^{H}}{K_{i}^{Na}P_{i}^{2}}$, (2.74)

$H_{Mg}=\frac{\partial[H_{bound}]}{\partial[Mg^{2+}]}=-[H^{+}]\sum_{i=1}^{Nr} \frac{[L_{i}]/K_{i}^{H}}{K_{i}^{Mg}P_{i}^{2}}$, (2.75)

$H_{Ca}=\frac{\partial[H_{bound}]}{\partial[Ca^{2+}]}=-[H^{+}]\sum_{i=1}^{Nr} \frac{[L_{i}]/K_{i}^{H}}{K_{i}^{Ca}P_{i}^{2}}$, (2.76)

$\frac{\partial[K_{bound}]}{\partial[K^{+}]}=\sum_{i=1}^{Nr} \frac{[L_{i}]\left(1+[H^{+}]/K_{i}^{H}+[Na^{+}]/K_{i}^{Na}+[Mg^{2+}]/K_{i}^{Mg}+[Ca^{2+}]/K_{i}^{Ca}\right)}{K_{i}^{K}P_{i}^{2}}$, (2.77)

$K_{H}=\frac{\partial[K_{bound}]}{\partial[H^{+}]}=-[K^{+}]\sum_{i=1}^{Nr} \frac{[L_{i}]/K_{i}^{K}}{K_{i}^{H}P_{i}^{2}}$, (2.78)

$K_{Na}=\frac{\partial[K_{bound}]}{\partial[Na^{+}]}=-[K^{+}]\sum_{i=1}^{Nr} \frac{[L_{i}]/K_{i}^{K}}{K_{i}^{Na}P_{i}^{2}}$, (2.79)

$K_{Mg}=\frac{\partial[K_{bound}]}{\partial[Mg^{2+}]}=-[K^{+}]\sum_{i=1}^{Nr} \frac{[L_{i}]/K_{i}^{K}}{K_{i}^{Mg}P_{i}^{2}}$, (2.80)

$K_{Ca}=\frac{\partial[K_{bound}]}{\partial[Ca^{2+}]}=-[K^{+}]\sum_{i=1}^{Nr} \frac{[L_{i}]/K_{i}^{K}}{K_{i}^{Ca}P_{i}^{2}}$, (2.81)

$\frac{\partial[Na_{bound}]}{\partial[Na^{+}]}=\sum_{i=1}^{Nr} \frac{[L_{i}]\left(1+[H^{+}]/K_{i}^{H}+[K^{+}]/K_{i}^{K}+[Mg^{2+}]/K_{i}^{Mg}+[Ca^{2+}]/K_{i}^{Ca}\right)}{K_{i}^{Na}P_{i}^{2}}$, (2.82)

$Na_{H}=\frac{\partial[Na_{bound}]}{\partial[H^{+}]}=-[Na^{+}]\sum_{i=1}^{Nr} \frac{[L_{i}]/K_{i}^{Na}}{K_{i}^{H}P_{i}^{2}}$, (2.83)

$Na_{K}=\frac{\partial[Na_{bound}]}{\partial[K^{+}]}=-[Na^{+}]\sum_{i=1}^{Nr} \frac{[L_{i}]/K_{i}^{Na}}{K_{i}^{K}P_{i}^{2}}$, (2.84)

, (2.85)

$Na_{Ca}=\frac{\partial[Na_{bound}]}{\partial[Ca^{2+}]}=-[Na^{+}]\sum_{i=1}^{Nr} \frac{[L_{i}]/K_{i}^{Na}}{K_{i}^{Ca}P_{i}^{2}}$, (2.86)

$\frac{\partial[Mg_{bound}]}{\partial[Mg^{2+}]}=\sum_{i=1}^{Nr} \frac{[L_{i}]\left(1+[H^{+}]/K_{i}^{H}+[K^{+}]/K_{i}^{K}+[Na^{+}]/K_{i}^{Na}+[Ca^{2+}]/K_{i}^{Ca}\right)}{K_{i}^{Mg}P_{i}^{2}}$, (2.87)

$Mg_{H}=\frac{\partial[Mg_{bound}]}{\partial[H^{+}]}=-[Mg^{2+}]\sum_{i=1}^{Nr} \frac{[L_{i}]/K_{i}^{Mg}}{K_{i}^{H}P_{i}^{2}}$, (2.88)

$Mg_{K}=\frac{\partial[Mg_{bound}]}{\partial[K^{+}]}=-[Mg^{2+}]\sum_{i=1}^{Nr} \frac{[L_{i}]/K_{i}^{Mg}}{K_{i}^{K}P_{i}^{2}}$, (2.89)

$Mg_{Na}=\frac{\partial[Mg_{bound}]}{\partial[Na^{+}]}=-[Mg^{2+}]\sum_{i=1}^{Nr} \frac{[L_{i}]/K_{i}^{Mg}}{K_{i}^{Na}P_{i}^{2}}$, (2.90)

$Mg_{Ca}=\frac{\partial[Mg_{bound}]}{\partial[Ca^{2+}]}=-[Mg^{2+}]\sum_{i=1}^{Nr} \frac{[L_{i}]/K_{i}^{Mg}}{K_{i}^{Ca}P_{i}^{2}}$, (2.91)

$\frac{\partial[Ca_{bound}]}{\partial[Ca^{2+}]}=\sum_{i=1}^{Nr} \frac{[L_{i}]\left(1+[H^{+}]/K_{i}^{H}+[K^{+}]/K_{i}^{K}+[Na^{+}]/K_{i}^{Na}+[Mg^{2+}]/K_{i}^{Mg}\right)}{K_{i}^{Ca}P_{i}^{2}}$, (2.92)

$Ca_{H}=\frac{\partial[Ca_{bound}]}{\partial[H^{+}]}=-[Ca^{2+}]\sum_{i=1}^{Nr} \frac{[L_{i}]/K_{i}^{Ca}}{K_{i}^{H}P_{i}^{2}}$, (2.93)

$Ca_{K}=\frac{\partial[Ca_{bound}]}{\partial[K^{+}]}=-[Ca^{2+}]\sum_{i=1}^{Nr} \frac{[L_{i}]/K_{i}^{Ca}}{K_{i}^{K}P_{i}^{2}}$, (2.94)

$Ca_{Na}=\frac{\partial[Ca_{bound}]}{\partial[Na^{+}]}=-[Ca^{2+}]\sum_{i=1}^{Nr} \frac{[L_{i}]/K_{i}^{Ca}}{K_{i}^{Na}P_{i}^{2}}$, (2.95)

$Ca_{Mg}=\frac{\partial[Ca_{bound}]}{\partial[Mg^{2+}]}=-[Ca^{2+}]\sum_{i=1}^{Nr} \frac{[L_{i}]/K_{i}^{Ca}}{K_{i}^{Mg}P_{i}^{2}}$, (2.96)

and

$P_{i}=1+[H^{+}]/K_{i}^{H}+[K^{+}]/K_{i}^{K}+[Na^{+}]/K_{i}^{Na}+[Mg^{2+}]/K_{i}^{Mg}+[Ca^{2+}]/K_{i}^{Ca}$. (2.97)

The flux terms (nmol/mg/min) into the system are defined as

$\Phi_{H}=-\sum_{i=1}^{Nr} \frac{\partial[H_{bound}]}{\partial[L_{i}]}\frac{d[L_{i}]}{dt}+\sum_{k=1}^{Nk} n_{k}J_{k}+J_{t}^{H}$, (2.98)

$\Phi_{Na}=-\sum_{i=1}^{Nr} \frac{\partial[Na_{bound}]}{\partial[L_{i}]}\frac{d[L_{i}]}{dt}+J_{t}^{Na}$, (2.99)

$\Phi_{K}=-\sum_{i=1}^{Nr} \frac{\partial[K_{bound}]}{\partial[L_{i}]}\frac{d[L_{i}]}{dt}+J_{t}^{K}$, (2.100)

$\Phi_{Mg}=-\sum_{i=1}^{Nr} \frac{\partial[Mg_{bound}]}{\partial[L_{i}]}\frac{d[L_{i}]}{dt}+J_{t}^{Mg}$ , (2.101)

and

$\Phi_{Ca}=-\sum_{i=1}^{Nr} \frac{\partial[Ca_{bound}]}{\partial[L_{i}]}\frac{d[L_{i}]}{dt}+J_{t}^{Ca}$, (2.102)

where *N_k_* is the number of reactions, *n_k_* is the stoichiometric coefficient of *k*^th^ reaction, *J_k_* is the *k*^th^ reaction rate and $J_{t}^{i}$ is the *i*^th^ cation transport rate into the system (the mitochondrial matrix).

The ligand dependent partial derivative expressions are defined as

$\frac{\partial[H_{bound}]}{\partial[L_{i}]}=[H^{+}]\sum_{i=1}^{Nr} \frac{1}{K_{i}^{H}P_{i}}$, (2.103)

$\frac{\partial[Na_{bound}]}{\partial[L_{i}]}=[Na^{+}]\sum_{i=1}^{Nr} \frac{1}{K_{i}^{Na}P_{i}}$, (2.104)

$\frac{\partial[K_{bound}]}{\partial[L_{i}]}=[K^{+}]\sum_{i=1}^{Nr} \frac{1}{K_{i}^{K}P_{i}}$, (2.105)

$\frac{\partial[Mg_{bound}]}{\partial[L_{i}]}=[Mg^{2+}]\sum_{i=1}^{Nr} \frac{1}{K_{i}^{Mg}P_{i}}$, (2.106)

and

$\frac{\partial[Ca_{bound}]}{\partial[L_{i}]}=[Ca^{2+}]\sum_{i=1}^{Nr} \frac{1}{K_{i}^{Ca}P_{i}}$. (2.107)

The generation of protons (nmol/mg/min) by biochemical reactions in the matrix is defined as

$\sum_{k=1}^{Nk} n_{k}J_{k}=\left(\left(1-2\right)J_{PDH}+2J_{CS}+\left(2-2\right)J_{IDH}-\left(1-2\right)J_{aKGDH}+J_{SCoAS}+J_{MDH}\right)$. (2.108)

The transport of protons in and out of the system (nmol/mg/min) is defined as

$J_{t}^{H}=\left(\begin{matrix} J_{PYRH}+J_{GLUTH}+J_{CITMAL}+\left(nH_{F_{1}F_{0}}-1\right)J_{F_{1}F_{0}}+2J_{PIC}+J_{mKHE}+J_{mNHE}+J_{mHleak} \\ -J_{GAE}-5J_{C1}-2J_{C3}-4J_{C4} \end{matrix}\right)$, (2.109)

$J_{t}^{Na}=\left(3J_{mNCE}-J_{mNHE}\right)$, (2.110)

$J_{t}^{K}=\left(J_{mKATP}+J_{mKleak}-J_{mKHE}\right)$, (2.111)

$J_{t}^{Mg}=0$, (2.112)

and

$J_{t}^{Ca}=\left(J_{UNI}-J_{mNCE}\right)$. (2.113)

S2D - Volume Dynamics

Mitochondria are considered well-behaved osmometers in a well-defined band of extra-mitochondrial osmolalities [27]; therefore, mitochondrial matrix water volume can be approximated based on the amount of osmotically active substances present inside the mitochondrial matrix. The IMS water volume can then be estimated after the matrix water volume is found assuming a total mitochondrial volume at specified osmolalitites is conserved.

Osmotically active matrix components consist of carbon substrates, nucleotides, matrix proteins, and ions. The osmotically active matrix anion content other than TCA cycle intermediates, adenine nucleotides and matrix proteins was assumed to mostly consist of the Cl^-^ anion. The Cl^-^ dynamics were not explicitly modeled; therefore, the matrix Cl^-^ content was estimated before calculating the matrix water volume.

The amount of Cl^-^ was estimated using first principles based on the charge accumulated on a capacitor at specified voltages. The following expression was derived to approximate the matrix Cl^-^ content,

$Cl^{-}=\sum_{j\in k_{m}} v_{j}S_{j}-C_{mito}\Delta\Psi$ (2.114)

where *k_m_* represents the set of indices of matrix solutes excluding Cl^-^, *v_j_* represents the average charge for solute *S_j_* where *S_j_* are all the solute state variables, including free cations but excluding Cl^-^, and C_mito_ represents the inner-mitochondrial membrane (IMM) capacitance. The average charge was computed for solute *S_j_* by summing the total charge of each species and dividing by the total amount of solute present at a given time. Binding polynomials were used to compute the concentration of each species. For example, the average charge of the solute, ATP, was computed as *v_ATP_* = -(4[ATP^4-^] + 3[KATP^3-^] + 3[NaATP^3-^] + 2[MgATP^2-^] + 2[CaATP^2-^])/[ATP] where [ATP] is the total ATP present in the matrix at a given time. Therefore, the remaining charge was assumed to be composed of matrix Cl^-^ anions and approximated using Equation 2.114.

With the estimated matrix Cl^-^ content, the osmotically active matrix solutes were summed

$S_{m}=\sum_{j\in i_{m}} S_{j}$, (2.115)

where *i_m_* represents the set of indices of osmotically active matrix solutes including Cl^-^.

Finally, the mitochondrial matrix water volume per mg of mitochondrial protein was estimated by

$V_{mtx}=\left(v_{0}+\frac{S_{m}}{\rho_{w}\varphi_{c}}\right)$ (2.116)

where *ν_0_* is the osmotically inactive volume, ρ_w_ is the water density and Φ_c_ is the extra-mitochondrial osmolality. The osmotically inactive volume has been experimentally approximated as 281 nl/mg [27], the water density is 0.996 g/ml at 30 ºC and the extra-mitochondrial osmolality was assumed to be fixed at 290 mOsm for all simulations unless specified.

The IMS water volume was approximated assuming mitochondrial total volume is constant at a fixed buffer osmolality [27]. The total mitochondrial volume can be divided up into three types of volumes; the total osmotically active volume; the packing volume, primarily the trapped medium; and a constant volume consisting of all the phospholipids, proteins, solutes and osmotically inactive matrix water [27]. The osmotically active water volume reaches a peak volume of about 1650 nl/mg when the external osmolality is approximately 115 mOsm. At this point, the OMM begins to rupture. The derivation of this peak volume was based on a mitochondrial particle volume of 0.43 μm^3^ for liver mitochondria, but the model is primarily based on heart mitochondria whose particle volume averages 0.6 μm^3^ [28]. Therefore, the maximum matrix water volume was adjusted to 2300 nl/mg for heart mitochondria. This value is consistent with the rat heart data from Kowaltowski et al. used to estimate the K^+^-cycle parameters [29]. When the osmolality is in the isosmotic range, the outer mitochondrial membrane remains intact and the IMS volume reciprocally changes with the matrix water volume. Thus, the IMS volume can be estimated as

$V_{ims}=2300-V_{mtx}$. (2.117)

**Part S3: Model Rate Equation Derivations**

For each reaction or transport mechanism, a brief introduction is given followed by the equations describing the rate expression. Previous authors’ contributions are explicitly identified in the text before the rate equation is presented. The parameter values are located in Tables S3.1-S3.26. The processes are described in the following order: TCA cycle and carbon substrate transport equations; IMS and extra-mitochondrial reactions; oxidative phosphorylation reactions; the K^+^-cycle and the Na^+^/Ca^2+^ dynamics.

S3A - TCA Cycle (Carbon Substrate Oxidation) Reaction Rates

The first enzyme that is discussed, although not explicitly part of the TCA cycle, is pyruvate dehydrogenase. Citrate synthase, the entry point into the TCA cycle, follows. Afterwards, aconitase through malate dehydrogenase are each discussed in detail. Glutamate-oxaloacetate transaminase then precedes the carbon substrate transport equations.

**Pyruvate dehydrogenase**

Pyruvate dehydrogenase (PDH) is responsible for the oxidative decarboxylation of pyruvate, transacylation of an acetyl group to CoA and production of reducing equivalents for the ETS. The mechanism chosen to model the reaction was the hexa-uni-ping-pong mechanism. The parameter values used for the rate expression are located in Table S3.1. The rate expression used in the model is based on Wu et al. [1] with a few notable modifications.

The binding constant for pyruvate was modified according to the data in Hucho et al. [30]. Also, the phosphorylation control over the dehydrogenase enzyme was included in the rate expression. This relationship was approximated in the rate expression by assuming that the acute regulation of PDH activity is mediated by the relative activities of pyruvate dehydrogenase phosphatase (PDP) and pyruvate dehydrogenase kinase (PDK). The phosphatase activates the enzyme and was assumed to be regulated by protons and divalent cations (Ca^2+^ and Mg^2+^) [30]. The kinase inhibits the enzyme and was assumed to be regulated by protons, pyruvate, MgATP and MgADP [30]. It was also assumed that the relative maximum activity of the phosphatase is approximately equal to that of the kinase [30].

The reference reaction is for pyruvate dehydrogenase is defined as

$[PYR^{-}]_{mtx}+[CoAS^{-}]_{mtx}+[NAD^{+}]_{mtx}+[H_{2}O]=[CO_{3}^{2-}]+[H^{+}]_{mtx}+[AcCoA]_{mtx}+[NADH]_{mtx}$,

the biochemical equation is

$[PYR]_{mtx}+[CoASH]_{mtx}+[NAD]_{mtx}+[H_{2}O]=[CO_{2,tot}]+[AcCoA]_{mtx}+[NADH]_{mtx}$.

and to simplify the notation, the reactants are redefined as follows: [A] = [PYR]_mtx_, [B] = [CoASH]_mtx_, [C] = [NAD]_mtx_, [P] = [CO_2_,_tot_], [Q] = [AcCoA]_mtx_ and [R] = [NADH]_mtx_.

Since the reaction catalyzed by the enzyme pyruvate dehydrogenase involves the generation of a proton, the apparent equilibrium constant for this reaction is defined as

$K_{eq}=e^{-\Delta_{r}G_{PDH}^{0}/RT}\left(\frac{P_{CO_{2,tot}}P_{AcCoA}P_{NADH}}{P_{PYR}P_{CoASH}P_{NAD}}\right)\frac{1}{[H^{+}]_{mtx}}$.

Pyruvate dehydrogenase is activated by pyruvate dehydrogenase phosphatase and inactivated by pyruvate dehydrogenase kinase. Both equations were derived assuming rapid equilibrium between the phosphatase and kinase with their respective effectors. A brief description follows.

The phosphatase was modeled as having a single divalent cation site capable of binding divalent cations. The major divalent cations present in the mitochondrial matrix are Mg^2+^ and Ca^2+^; therefore, only these cations were considered. Also, when Ca^2+^ is bound instead of Mg^2+^, the enzyme is less efficient at dephosphorylating PDH (*β_Ca_*<1). The kinase was modeled as having a single adenine dinucleotide binding site capable of binding the Mg-chelated forms of ATP or ADP. With MgATP bound, the kinase is able to phoshporylate PDH. There is also an allosteric site capable of binding pyruvate that lowers both the apparent K_M_ of the adenine dinucleotides and the phosphorylating rate. The pyruvate and adenine dinucleotide binding events were assumed to be independent of each other. Both PDP and PDK were assumed to posses two histidine amino side groups near the active site with pK_a_s near pH 7 that are responsible for the biphasic pH dependence seen in Figure S3.1A.

The phosphatase regulation is defined as

$$a_{PDP}=\frac{\left(\frac{[Mg^{2+}]_{mtx}}{K_{Mg}}+\frac{\beta_{Ca}[Ca^{2+}]_{mtx}}{K_{Ca}}\right)}{\left(\frac{[H^{+}]_{mtx}}{K_{Hpdp}}+\frac{K_{Hpdp}}{[H^{+}]_{mtx}}\right)\left(1+\frac{[Mg^{2+}]_{mtx}}{K_{Mg}}+\frac{[Ca^{2+}]_{mtx}}{K_{Ca}}\right)}$$

and the kinase regulation is defined as

$a_{PDK}=\frac{\frac{[MgATP^{2-}]_{mtx}}{K_{MgATP}}}{\left(\frac{[H^{+}]_{mtx}}{K_{Hpdk}}+\frac{K_{Hpdk}}{[H^{+}]_{mtx}}\right)\left(1+\frac{[A]}{K_{iPYR}}\right)\left(1+\frac{[MgATP^{2-}]_{mtx}}{K_{MgATP}}+\frac{[MgADP^{-}]_{mtx}}{K_{MgADP}}\right)}$.

The reaction rate is a function of the maximum reaction rate and the ratio of activation versus inactivation defined by

$V_{PDH}=\frac{V_{\max}}{1+a_{PDK}/a_{PDP}}$.

The full rate expression for pyruvate dehydrogenase is

$J_{PDH}=\frac{V_{PDH}\left([A][B][C]-[P][Q][R]/K_{eq}\right)}{K_{C}\left(1+\left[R\right]/K_{iR}\right)[A][B]+K_{B}\left(1+\left[Q\right]/K_{iQ}\right)[A][C]+K_{A}[B][C]+[A][B][C]}$.

**Table S3.1.** Pyruvate Dehydrogenase Parameters

| Parameter | Definition | Value | Reference |
| --- | --- | --- | --- |
| *V_max_* | Maximum reaction rate (T=25°C) | 127 nmol/min/mg | a |
| $\Delta_{r}G_{PDH}^{0}$ | Gibbs free energy of reaction | 19.59 kJ/mol | [1] |
| *K_A_* | PYR binding constant | 33.2x10^-6^ M | [30]^b^ |
| *K_B_* | CoASH binding constant | 9.90x10^-6^ M | [1] |
| *K_C_* | NAD binding constant | 60.7x10^-6^ M | [1] |
| *K_iQ_* | AcCoA inhibition constant | 40.2x10^-6^ M | [1] |
| *K_iR_* | NADH inhibition constant | 40.0x10^-6^ M | [1] |
| *K_Hpdp_* | H^+^ binding constant for the phosphatase | 10^-7.13^ M | [30]^b^ |
| *K_Hpdk_* | H^+^ binding constant for the kinase | 10^-7.02^ M | [30]^b^ |
| *K_Mg_* | Mg^2+^ binding constant | 1.57x10^-3^ M | [30]^b^ |
| *K_Ca_* | Ca^2+^ binding constant | 80.1x10^-6^ M | [30]^b^ |
| *β_Ca_* | Ca^2+^ activation constant | 0.220 (unitless) | [30]^b^ |
| *K_iPYR_* | PYR inhibition constant | 88.1x10^-6^ M | [30]^b^ |
| *K_MgADP_* | MgADP binding constant | 97.6x10^-6^ M | [30]^b^ |
| *K_MgATP_* | MgATP binding constant | 17.6x10^-6^ M | [30]^b^ |

^a^ Fit to simulation using the experimental data sets described in the main paper.

^b^ Fit to data presented in the reference as briefly described below.

Figure S3.1 shows the model fits to Hucho et al.’s [30] data on the regulation of PDH by its respective phosphatase and kinase. In short, PDH purified from bovine heart was incubated in the presence of its activator, PDP, or inhibitor, PDK, before the initial rate assays using saturating substrates were carried out. In this respect, the initial rates measured correspond to the initial rate of PDH activation or inhibition, depending on the incubation conditions with PDP or PDK, respectively. A total of 17 parameters (9 kinetic and 8 maximum rate terms) were fit to 63 data points from 8 data curves. Although 8 maximum rate terms were used in the parameter estimation for each figure, their standard deviations were small relative to the mean for each reaction type: the PDP maximum rates were 1.18 +/- 0.11 ΔA_340_/min (n=5), the PDK maximum rates were 0.81 +/- 0.17 ΔA_340_/min (n=2) and the PDH maximum activity was 0.37 ΔA_340_/min (n=1).

**Figure S3.1. The fit pyruvate dehydrogenase rate (as indicated by the NADH production rate, ΔA_340_/min) is compared with the experimental data from Hucho et al. [30] for different regulatory conditions.**  Pyruvate dehydrogenase was isolated from bovine heart and initial rate assays were performed. (A) The reaction rate of the pyruvate dehydrogenase was measured as a function of pH in the presence of phosphatase activity (circles) mediated by 10 mM Mg^2+^ and kinase activity (squares) mediated by 0.5 mM Mg^2+^. (B) The rate in the presence of kinase activity was measured as a function of ATP (circles), ATP + 0.5 mM ADP (squares) and ATP + 0.5 mM PYR (diamonds) at pH 7.0. (C) The rate in the presence of phosphatase activity was measured as a function of Mg^2+^ and (D) Ca^2+^ at pH 7.0.

The phosphatase rate expression was corroborated using the Pi-titration curves. Figure S3.2 shows that the apparent Pi inhibition of PDP was primarily due to the complexation of MgPi effectively removing free Mg^2+^ from the buffer and not a specific interaction between Pi and the phosphatase. The simple rapid equilibrium reaction mechanism (*a*_PDP_) is able to capture the apparent inhibition of Pi and reproduce the experimental data rather well. When an explicit Pi binding site was included in the reaction mechanism, no significant improvement in the quality of the fits was observed.

**Figure S3.2. The PDP rate expression (as indicated by the NADH production rate, ΔA_340_/min) is compared with the apparent Pi inhibition experimental data from Hucho et al. [30].** PDP was incubated with either 5 mM Mg^2+^ (solid, circle) or 10 mM Mg^2+^ (dotted, square) as the Pi buffer concentration was varied from 0 to 50 mM. Minor discrepancies between the data and model were attributed to the dissociation constant uncertainties discussed in Part S1C.

**Citrate synthase**

Citrate synthase is responsible for the condensation reaction of acetyl coenzyme-A and oxaloacetate to form citrate. This enzyme is often referred to as the pace-making enzyme considering that it’s the first enzyme in the TCA cycle and heavily regulated. We used the reaction mechanism, binding constants and free energies from Wu et al. [1]. The parameter values are located in Table S3.2. For completeness, the rate expression from Wu et al. is presented below.

The reference reaction for citrate synthase is defined as

$[OAA^{2-}]_{mtx}+[AcCoA]_{mtx}+[H_{2}O]=[CoAS^{-}]_{mtx}+[CIT^{3-}]_{mtx}+2[H^{+}]_{mtx}$,

the biochemical reaction is

$$[OAA]_{mtx}+[AcCoA]_{mtx}+[H_{2}O]=[CoASH]_{mtx}+[CIT]_{mtx}$$

and to simplify the notation, the reactants are redefined as follows: [A] = [OAA]_mtx_, [B] = [AcCoA]_mtx_, [P] = [CoASH]_mtx_ and [Q] = [CIT]_mtx_.

Since the reaction catalyzed by the enzyme citrate synthase involves the generation of two protons, the pH-dependent equilibrium constant for this reaction is defined as

$K_{eq}=e^{-\Delta_{r}G_{CS}^{0}/RT}\frac{1}{[H^{+}]_{mtx}^{2}}\frac{P _{CoASH}P_{CIT}}{P_{OAA}P_{AcCoA}}$.

The overall rate expression for citrate synthase used in the model is

$$J_{CS}=\frac{V_{\max}\left([A][B]-[P][Q]/K_{eq}\right)}{\left[K_{iA}K_{mB}\alpha_{i1}+K_{mA}\alpha_{i1}[B]+K_{mB}\alpha_{i2}[A]+[A][B]\right]}$$

where

$$\alpha_{i1}=1+\frac{[CIT^{3-}]_{mtx}}{K_{iCIT}}$$

and

$\alpha_{i2}=1+\frac{[ATP^{4-}]_{mtx}}{K_{iATP}}+\frac{[ADP^{3-}]_{mtx}}{K_{iADP}}+\frac{[AMP^{2-}]_{mtx}}{K_{iAMP}}+\frac{[CoASH]_{mtx}}{K_{iCoASH}}+\frac{[ScoA]_{mtx}}{K_{iSCoA}}$.

**Table S3.2.** Citrate Synthase Parameters

| Parameter | Definition | Value | Reference |
| --- | --- | --- | --- |
| *V_max_* | Maximum reaction rate (T=25°C) | 584 nmol/min/mg | a |
| $\Delta_{r}G_{CS}^{0}$ | Gibbs free energy of reaction | 42.36 kJ/mol | [1] |
| *K_mA_* | OAA binding constant | 4.00x10^-6^ M | [1] |
| *K_mB_* | AcCoA binding constant | 14.0x10^-6^ M | [1] |
| *K_iA_* | OAA inhibition constant | 3.33x10^-6^ M | [1] |
| *K_iQ_* | CIT inhibition constant | 1.60x10^-3^ M | [1] |
| *K_iATP_* | ATP inhibition constant | 0.90x10^-3^ M | [1] |
| *K_iADP_* | ADP inhibition constant | 1.80x10^-3^ M | [1] |
| *K_iAMP_* | AMP inhibition constant | 0.60x10^-3^ M | [1] |
| *K_iCoASH_* | CoASH inhibition constant | 67.0x10^-6^ M | [1] |
| *K_iSCoA_* | ScoA inhibition constant | 140x10^-6^ M | [1] |

^a^ Fit to simulation using the experimental data sets described in the main paper.

**Aconitase**

Aconitase is responsible for the liberation of a water molecule from citrate to form isocitrate. We used the reaction mechanism, binding constants and free energies from Wu et al. [1]. The parameter values are located in Table S3.3. For completeness, the rate expression from Wu et al. is presented below.

The reference reaction for aconitase is

$[CIT^{3-}]_{mtx}=[ISOC^{3-}]_{mtx}+[H_{2}O]$,

the biochemical equation is

$$[CIT]_{mtx}=[ISOC]_{mtx}+[H_{2}O]$$

and to simplify the notation, the reactants are redefined as follows: [A] = [CIT]_mtx_ and [P] = [ISOC]_mtx_.

The apparent equilibrium constant for this reaction is defined as

$K_{eq}=e^{-\Delta_{r}G_{ACH}^{0}/RT}\left(\frac{P_{CIT}}{P_{ISOC}}\right)$,

and the overall rate expression for citrate synthase used in the model is

$$J_{ACH}=\frac{V_{mf}V_{mr}\left([A]-[P]/K_{eq}\right)}{\left[V_{mr}K_{mA}+V_{mr}[A]+\frac{V_{mf}[P]}{K_{eq}}\right]}$$

where the reverse rate is defined using the Haldane constraint,

$V_{mr}=\frac{V_{mf}K_{mP}K_{iQ}}{K_{eq}K_{iA}K_{mB}}$.

**Table S3.3.** Aconitase Parameters

| Parameter | Definition | Value | Reference |
| --- | --- | --- | --- |
| *V_mf_* | Maximum forward reaction rate (T=25°C) | 1.16x10^5^ nmol/min/mg | a |
| $\Delta_{r}G_{ACH}^{0}$ | Gibbs free energy of reaction | 6.65 kJ/mol | [1] |
| *K_mA_* | CIT binding constant | 1.20x10^-3^ M | [1] |
| *K_mP_* | ISOC binding constant | 434x10^-6^ M | [1] |

^a^ Fit to simulation using the experimental data sets described in the main paper.

**Isocitrate dehydrogenase**

Isocitrate dehydrogenase is responsible for the oxidative decarboxylation of isocitrate to produce α-ketoglutarate and reducing equivalents for the ETS. The rate expression used in the model is from Qi et al. [11], except that it is assumed that 1^st^ order binding polynomials are sufficient to capture the salient regulatory mechanisms of the reaction. Also, since EGTA is not present inside the mitochondrial matrix, the EGTA binding kinetics was removed from the rate expression.

Qi et al. fit the model parameters to a wide variety of data sets including divalent cations (Mg^2+^ and Ca^2+^), pH and adenine nucleotide dependencies; however, they generated several parameter sets and rate expressions that were species dependent. Each data set did not contain sufficient data to uniquely parameterize all the parameters used for the rate expression in this extended model. Thus, we generated a global set of parameters for their proposed reaction mechanism by averaging multiple values for the same parameter. The parameter values are located in Table S3.4. For completeness, the compiled rate expression from Qi et al. [11] is presented below.

The reference reaction for isocitrate dehydrogenase is

$[NAD^{+}]_{mtx}+[ISOC^{3-}]_{mtx}+[H_{2}O]=[CO_{3}^{2-}]+[NADH]_{mtx}+2[H^{+}]_{mtx}$,

the biochemical equation is

$$[NAD]_{mtx}+[ISOC]_{mtx}+[H_{2}O]=[CO_{2,tot}]+[NADH]_{mtx}$$

and to simplify the notation, the reactants are redefined as follows: [A] = [NAD^+^]_mtx_, [B] = [MgICIT^-^]_mtx_, [P] = [CO_3_^2-^] and [Q] = [NADH]_mtx_.

Since the reaction catalyzed by the enzyme isocitrate dehydrogenase involves the generation of two protons, the pH-dependent equilibrium constant for this reaction is defined as

$K_{eq}=e^{-\Delta_{r}G_{IDH}^{0}/RT}\frac{1}{[H^{+}]_{mtx}^{2}}$.

The overall rate expression for isocitrate dehydrogenase used in the model is

$$J_{IDH}=\frac{V_{\max}/\left(1+[H^{+}]_{mtx}/K_{iH}\right)\left(1-\frac{[P][Q]}{[A][B]K_{eq}}\right)}{\left[1+\frac{K_{mA}}{[A]}\left(1+\frac{[Q]}{K_{iq}}\right)\alpha_{1}+\left(\frac{K_{mB}}{[B]}\right)^{n}+\frac{K_{iA}}{[A]}\left(\frac{K_{mB}}{[B]}\right)^{n}\left(1+\frac{[Q]}{K_{iq}}\right)\right]}$$

where

$\alpha_{2}=\frac{1+\frac{[CaATP^{2-}]_{mtx}}{K_{aCaATP}}+\frac{[CaADP^{-}]_{mtx}}{K_{aCaADP}}+\frac{[ADP^{3-}]_{mtx}}{K_{aADP}}}{1+\frac{[CaATP^{2-}]_{mtx}}{a_{1}K_{aCaATP}}+\frac{[CaADP^{-}]_{mtx}}{a_{2}K_{aCaADP}}+\frac{[ADP^{3-}]_{mtx}}{a_{3}K_{aADP}}}$.

**Table S3.4.** Isocitrate Dehydrogenase Updated Parameters

| Parameter | Definition | Value | Reference |
| --- | --- | --- | --- |
| *V_max_* | Maximum reaction rate (T=25°C) | 6.84x10^4^ nmol/min/mg | a |
| $\Delta_{r}G_{IDH}^{0}$ | Gibbs free energy of reaction | 91.77 kJ/mol | [11] |
| *K_mA_* | NAD binding constant | 703.3x10^-6^ M | [11]^b^ |
| *K_mB_* | MgISOC binding constant | 158.7x10^-6^ M | [11]^b^ |
| *K_ia_* | NAD inhibition constant | 65.2x10^-6^ M | [11]^b^ |
| *K_aCaADP_* | CaADP binding constant | 33.1x10^-6^ M | [11]^b^ |
| *K_aCaATP_* | CaATP binding constant | 289x10^-6^ M | [11] |
| *K_aADP_* | ADP binding constant | 124x10^-6^ M | [11]^b^ |
| *K_iq_* | NADH inhibition constant | 4.75x10^-6^ M | [11] |
| *K_iH_* | H^+^ inhibition constant | 10^-6.96^ M | [11] |
| *a_1_* | K_aCaADP_ modulation constant | 9.00x10^-4^ (unitless) | [11]^b^ |
| *a_2_* | K_aCaATP_ modulation constant | 9.70x10^-3^ (unitless) | [11] |
| *a_3_* | K_aADP_ modulation constant | 7.00x10^-4^(unitless) | [11]^b^ |
| *n* | MgISOC cooperativity constant | 3 | [11] |

^a^ Fit to simulation using the experimental data sets described in the main paper.

^b^ The value was averaged over the parameter sets listed in the reference.

**α-Ketoglutarate dehydrogenase**

α-Ketoglutarate dehydrogenase (αKGDH) is a key TCA regulatory enzyme that is responsible for the oxidative decarboxylation of α-ketoglutarate, transfering a succinyl group to CoA and producing reducing equivalents for the ETS. We were able to fit the data with the consensus hexa-uni-ping-pong mechanism and reproduce a wide variety of data [31-34]. When necessary, the cation/anion species distribution for each experimental condition was calculated by assuming rapid equilibrium between the various cation and anion species. The model was able to reproduce the known pH, divalent cation (Ca^2+^ and Mg^2+^), adenine nucleotide and Pi dependence seen experimentally. Each data set was derived from the porcine heart isoform of α-ketoglutarate dehydrogenase. The parameter values used for the rate expression are located in Table S3.5.

The reference reaction for αKGDH is

$[\alpha KG^{2-}]_{mtx}+[CoAS^{-}]_{mtx}+[NAD^{+}]_{mtx}+[H_{2}O]=[CO_{3}^{2-}]+[SCoA^{-}]_{mtx}+[NADH]_{mtx}+[H^{+}]_{mtx}$,

the biochemical equation is

$$[\alpha KG]_{mtx}+[CoASH]_{mtx}+[NAD]_{mtx}+[H_{2}O]=[CO_{2,tot}]+[SCoA]_{mtx}+[NADH]_{mtx}$$

and to simplify the notation, the reactants are redefined as follows: [A] = [αKG]_mtx_, [B] = [CoASH]_mtx_, [C] = [NAD]_mtx_, [P] = [CO_2_,_tot_], [Q] =[SCoA]_mtx_ and [R] = [NADH]_mtx_.

Since the reaction catalyzed by αKGDH involves the generation of a proton, the apparent equilibrium constant for this reaction is defined as

$K_{eq}=e^{-\Delta_{r}G_{aKGDH}^{0}/RT}\left(\frac{P_{CO_{2,tot}}P_{SCoA}P_{NADH}}{P_{\alpha KG}P_{CoASH}P_{NAD}}\right)\frac{1}{[H^{+}]_{mtx}}$.

The αKGDH affinity for α-ketoglutarate is modulated by H^+^, Ca^2+^, adenine nucleotides, and inorganic phosphate. The steady state hexa-uni-ping-pong rate equation was hybridized using the rapid equilibrium method concerning the various effectors. Protons were treated as essential activators for the enzyme complex, while Ca^2+^, adenine nucleotides and Pi were defined as non-essential, independent activators. A single adenine nucleotide site was used with varying affinities for fADP, MgADP, fATP and MgATP respectively. When Mg^2+^ is bound to the enzyme complex, the Ca^2+^ affinity is amplified and enhances the Ca^2+^ stimulatory effect via an increase in α-ketoglutarate affinity. The α-ketoglutarate binding constant is modeled as

$$K_{A}=K_{A0}\frac{K_{H}}{[H^{+}]_{mtx}}\left[\begin{matrix} \left(\frac{1+\frac{[fADP]_{mtx}}{K_{ADP}}+\frac{[MgADP]_{mtx}}{K_{MgADP}}+\frac{[fATP]_{mtx}}{K_{ATP}}+\frac{[MgATP]_{mtx}}{K_{MgATP}}}{1+\frac{[fADP]_{mtx}}{\alpha_{ADP}K_{ADP}}+\frac{[MgADP]_{mtx}}{\alpha_{MgADP}K_{MgADP}}+\frac{[fATP]_{mtx}}{\alpha_{ATP}K_{ATP}}+\frac{[MgATP]_{mtx}}{\alpha_{MgATP}K_{MgATP}}}\right) \\ \left(\frac{1+\frac{[Ca^{2+}]_{mtx}}{K_{Ca}}+\frac{[Mg^{2+}]_{mtx}}{K_{Mg}}\frac{[Ca^{2+}]_{mtx}}{K_{Ca}}}{1+\frac{[Ca^{2+}]_{mtx}}{\alpha_{Ca}K_{Ca}}+\frac{[Mg^{2+}]_{mtx}}{\alpha_{Mg}K_{Mg}}\frac{[Ca^{2+}]_{mtx}}{K_{Ca}}}\right)\left(\frac{1+\frac{[Pi^{2-}]_{mtx}}{K_{Pi}}}{1+\frac{[Pi^{2-}]_{mtx}}{\alpha_{Pi}K_{Pi}}}\right) \end{matrix}\right]$$

where

$$[fL]_{mtx}=[L_{tot}]\left(1+[H^{+}]_{mtx}/KD_{L}^{H}+[K^{+}]_{mtx}/KD_{L}^{K}+[Na^{+}]_{mtx}/KD_{L}^{Na}\right)/P_{L}$$

for any ligand *L*. This notation will be used throughout this manuscript. For example,

$[fADP]_{mtx}=[ADP]_{mtx}\left(1+[H^{+}]_{mtx}/KD_{ADP}^{H}+[K^{+}]_{mtx}/KD_{ADP}^{K}+[Na^{+}]_{mtx}/KD_{ADP}^{Na}\right)/P_{ADP}$.

The maximum turnover rate for αKGDH is modulated by Mg^2+^, adenine nucleotides and inorganic phosphate. The apparent maximum reaction rate is modeled as

$V_{app}=V_{\max}\left[\begin{matrix} \left(\frac{1+\frac{\beta_{ADP}[fADP]_{mtx}}{K_{ADP}}+\frac{\beta_{MgADP}[MgADP]_{mtx}}{K_{MgADP}}+\frac{\beta_{ATP}[fATP]_{mtx}}{K_{ATP}}+\frac{\beta_{MgATP}[MgATP]_{mtx}}{K_{MgATP}}}{1+\frac{[fADP]_{mtx}}{\alpha_{ADP}K_{ADP}}+\frac{[MgADP]_{mtx}}{\alpha_{MgADP}K_{MgADP}}+\frac{[fATP]_{mtx}}{\alpha_{ATP}K_{ATP}}+\frac{[MgATP]_{mtx}}{\alpha_{MgATP}K_{MgATP}}}\right) \\ \left(\frac{1+\frac{\beta_{Mg}[Mg^{2+}]_{mtx}}{K_{Mg}}}{1+\frac{[Mg^{2+}]_{mtx}}{K_{Mg}}}\right)\left(\frac{1+\frac{\beta_{Pi}[Pi^{2-}]_{mtx}}{K_{Pi}}}{1+\frac{[Pi^{2-}]_{mtx}}{\alpha_{Pi}K_{Pi}}}\right) \end{matrix}\right]$.

The overall rate expression for αKGDH used in the model is

.

**Table S3.5.** α-ketoglutarate Dehydrogenase Parameters

| Parameter | Definition | Value | Reference |
| --- | --- | --- | --- |
| $V_{\max}$ | Maximum reaction rate (T=25°C) | 779 nmol/min/mg | a |
| $\Delta_{r}G_{\alpha KDH}^{0}$ | Gibbs free energy of reaction | -73.13 kJ/mol | [1] |
| *K_A0_* | αKG binding constant | 6.30 x10^-3^ M | [31-34]^b^ |
| *K_B_* | CoASH binding constant | 2.91x10^-6^ M | [31-34]^b^ |
| *K_C_* | NAD binding constant | 95.5x10^-6^ M | [31-34]^b^ |
| *K_iC_* | NAD inhibition constant | 74.3x10^-6^ M | [31-34]^b^ |
| *K_iR_* | NADH inhibition constant | 15.3x10^-6^ M | [31-34]^b^ |
| *K_iQ_* | SCoA inhibition constant | 6.90x10^-6^ M | [1] |
| *K_H_* | H^+^ binding constant | 10^-7.36^ M | [31-34]^b^ |
| *K_Ca_* | Ca^2+^ binding constant | 61.6x10^-6^ M | [31-34]^b^ |
| *K_Mg_* | Mg^2+^ binding constant | 4.54x10^-6^M | [31-34]^b^ |
| *K_ATP_* | fATP binding constant | 198.5x10^-6^ M | [31-34]^b^ |
| *K_ADP_* | fADP binding constant | 123.5x10^-6^ M | [31-34]^b^ |
| *K_MgATP_* | MgATP binding constant | 150.9x10^-6^ M | [31-34]^b^ |
| *K_MgADP_* | MgADP binding constant | 91.3x10^-6^ M | [31-34]^b^ |
| *K_Pi_* | Pi binding constant | 7.70x10^-3^ M | [31-34]^b^ |
| *α_Ca_* | Ca^2+^ kinetic constant | 0.02 unitless | [31-34]^b^ |
| *a_Mg_* | Mg^2+^ kinetic constant | 0.08 unitless | [31-34]^b^ |
| *β_Mg_* | Mg^2+^ catalytic constant | 4.55 unitless | [31-34]^b^ |
| *α_ATP_* | ATP kinetic constant | 0.32 unitless | [31-34]^b^ |
| *β_ATP_* | ATP catalytic constant | 0.38 unitless | [31-34]^b^ |
| *α_ADP_* | ADP kinetic constant | 0.11 unitless | [31-34]^b^ |
| *β_ADP_* | ADP catalytic constant | 0.80 unitless | [31-34]^b^ |
| *α_MgATP_* | ATP kinetic constant | 0.58 unitless | [31-34]^b^ |
| *β_MgATP_* | ATP catalytic constant | 0.33 unitless | [31-34]^b^ |
| *α_MgADP_* | ADP kinetic constant | 25.1 unitless | [31-34]^b^ |
| *β_MgADP_* | ADP catalytic constant | 4.08 unitless | [31-34]^b^ |
| *α_Pi_* | Pi kinetic constant | 0.08 unitless | [31-34]^b^ |
| *β_Pi_* | Pi catalytic constant | 0.62 unitless | [31-34]^b^ |

^a^ Fit to simulation using the experimental data sets described in the main paper.

^b^ Fit to data presented in the reference as briefly described below.

The consensus mechanism with appropriate activator/inhibitor modifications was sufficient to reproduce the data as shown in Figures S3.2-S3.5. Due to the shear volume of data used to parameterize the rate expression, not all of the results are shown. The rate expression for αKGDH consists of 27 parameters. We used 56 data curves from four independent data sets, Panov and Scarpa [32], McCormack and Denton [33], McMinn and Ottaway [31] and Rodriguez-Zavala et al. [34] to parameterize the rate expression. The temperature for each study was approximately 30°C. A total of 82 parameters (27 kinetic and 55 maximum rate terms) were fit to the four independent data sets. Although 55 maximum rate terms were used in the parameter estimation for each figure in the original sources, their standard deviations were small (<15%) relative to the mean for each data set: 2.06 +/- 0.21 μM NADH/min/20 μl enzyme solution (n=20) for the Panov and Scarpa data set, 2.94 +/- 0.33 μmol/min/mg (n=13) for the McCormack and Denton data set, 2.83 +/- 0.34 μmol/min/mg (n=20) for the McMinn and Ottaway data set, and 178.08 +/- 23.26 (n=12) nmol/min/mg for the Rodriguez-Zavala et al. data set.

**Figure S3.2. The fit αKGDH rate (as indicated by the NADH production rate, µmol/min/mg) is compared with the experimental data from McMinn and Ottaway [31].** α-Ketoglutarate dehydrogenase was isolated from porcine heart and initial rate assays were performed using combinations of varied substrates and end-product inhibitors at pH 7.2. (A) αKG was varied in the presence of 333 µM NAD, 50 µM CoA (circle); 133 µM NAD, 20 µM CoA (square); 66 µM NAD, 10 µM CoA (diamond) and 33 µM NAD, 5 µM CoA (triangle). (B) CoA was varied in the presence of 500 µM αKG, 400µM NAD (circle); 100 µM αKG, 80 µM NAD (square); 50 µM αKG, 40 µM NAD (diamond) and 25 µM αKG, 20 µM NAD (triangle). (C) NAD was varied in the presence of 500 µM αKG, 50 µM CoA (circle); 200 µM αKG, 20 µM CoA (square); 100 µM αKG, 10 µM CoA (diamond) and 50 µM αKG, 5 µM CoA (triangle). (D) NAD was varied with saturating αKG and CoA (500 µM αKG, 50 µM CoA) in the presence of 0 µM NADH (circle), 10 µM NADH (square), 20 µM NADH (diamond) and 50 µM NADH (triangle).

**Figure S3.3. The fit αKGDH rate (as indicated by the NADH production rate, µmol/min/mg) is compared with the experimental data from McCormack and Denton [33].** α-Ketoglutarate dehydrogenase was isolated from porcine heart and initial rate assays were performed using combinations of activators and inhibitors. (A) αKG was varied in the presence of different free calcium concentrations at pH 6.8 with 1 mM NAD, 0.25 mM CoA, 1 mM MgCl_2_, 5 mM EGTA and 0 mM CaCl_2_ (circles) or 5 mM CaCl_2_ (squares). The free calcium concentrations were calculated to be < 1 nM and 32.7 µM, respectively. (B) ADP (circles) or ATP (squares) at pH 7 was varied in the presence of 0.1 mM αKG, 1 mM NAD, 0.25 mM CoA, 1 mM MgCl2, 5 mM EGTA and 5 mM CaCl_2_. The free calcium concentration was calculated for each ADP and ATP addition. (C) ADP (circles), ATP (squares) or ADP with 1.5 mM ATP (diamonds) at pH 7 was varied in the presence of 2 mM αKG, 1 mM NAD, 0.25 mM CoA, 1 mM MgCl_2_ and 5 mM EGTA. (D) The ratio of ADP to ATP at pH 7 was varied in the presence of 1 mM NAD, 0.25 mM CoA, 1 mM MgCl_2_ and 0.1 mM αKG, 5 mM EGTA, and 5 mM CaCl_2_ (circles) or 2 mM αKG and 5 mM EGTA (squares). The total adenine nucleotide concentration was fixed at 1.5 mM. When CaCl_2_ was present, the free calcium concentration was calculated for each ADP/ATP addition.

**Figure S3.4. The fit αKGDH rate (as indicated by the NADH production rate, µM NADH/min/20 µl enzyme) is compared with the experimental data from Panov and Scarpa [32].** α-Ketoglutarate dehydrogenase was isolated from porcine heart and initial rate assays were performed using combinations of varied substrates and divalent cation activators at pH 7.1. (A) Free Mg^2+^ was varied in the presence of 1 mM NAD, 0.25 mM CoA and 0.1 mM EGTA with either 0.5 mM αKG (circles) or 1 mM αKG (squares). (B) αKG was varied in the presence of 25 mM αKG, 1 mM NAD, 0.25 mM CoA, 0.1 mM EGTA with either 0 mM Mg^2+^ (circles) or 0.38 mM Mg^2+^ (squares).

\

**Figure S3.5.**  **The fit α-ketoglutarate dehydrogenase rate (as indicated by the NADH production rate, nmol NADH/min/mg) is compared with the experimental data from Rodriguez-Zavala et al. [34].** α-Ketoglutarate dehydrogenase was isolated from porcine heart and initial rate assays were performed using combinations of varied substrates and activators at pH 7.35. (A) ADP was varied in the presence of 1 mM NAD, 0.25 mM CoA, 0.5 mM αKG and 0.1 mM EGTA with either no additions (circles), 25 μM Mg^2+^ (squares), 50 μM Mg^2+^ (diamonds) or 200 μM Mg^2+^ (triangles). (B) αKG was varied in the presence of 1 mM NAD, 0.25 mM CoA and 0.1 mM EGTA at pH 7.35 with either no additions (circles), 5 mM Pi (squares), 600 μM ADP (diamonds) or 600 μM ATP (triangles). (C) αKG was varied in the presence of 1 mM NAD, 0.25 mM CoA, 0.1 mM EGTA and 600 μM MgCl_2_ at pH 7.35 with either no additions (circles), 5 mM Pi (squares), 600 μM ADP (diamonds) or 600 μM ATP (triangles).

**Succinyl coenzyme-A synthetase**

Succinyl coenzyme-A synthetase (SCoAS) is responsible for the formation of succinate and coenzyme-A from succinyl-CoA capitalizing on the energy to phosphorylate GDP. The reaction mechanism, binding constants and free energies are from Wu et al. [1]. The parameter values are located in Table S3.6. For completeness, the rate expression from Wu et al. is presented below.

The reference reaction for succinyl coenzyme-A synthetase is

$[GDP^{3-}]_{mtx}+[SCoA^{-}]_{mtx}+[Pi^{2-}]_{mtx}=[CoAS^{-}]_{mtx}+[SUC^{2-}]_{mtx}+[GTP^{4-}]_{mtx}+[H^{+}]_{mtx}$,

the biochemical equation is

$$[GDP]_{mtx}+[SCoA]_{mtx}+[Pi]_{mtx}=[CoASH]_{mtx}+[SUC]_{mtx}+[GTP]_{mtx}$$

and to simplify the notation, the reactants are redefined as follows: [A] = [GDP]_mtx_, [B] = [SCoA]_mtx_, [C] = [Pi]_mtx_, [P] = [CoASH]_mtx_, [Q] = [SUC]_mtx_ and [R] = [GTP]_mtx_.

Since the reaction catalyzed by SCoAS involves the generation of a proton, the apparent equilibrium constant for this reaction is defined as

$K_{eq}=e^{-\Delta_{r}G_{SCoAS}^{0}/RT}\left(\frac{P_{CoASH}P_{SUC}P_{GTP}}{P_{GDP}P_{SCoA}P_{Pi}}\right)\frac{1}{[H^{+}]_{mtx}}$.

The overall rate expression for SCoAS used in the model is

$$J_{SCoAS}=\frac{V_{mf}V_{mr}\left([A][B][C]-[P][Q][R]/K_{eq}\right)}{\left[\begin{matrix} V_{mr}K_{iA}K_{iB}K_{mC}+V_{mr}K_{iB}K_{mC}[A]+V_{mr}K_{iA}K_{mB}[C]+V_{mr}K_{mC}[A][B]+V_{mr}K_{mB}[A][C] \\ +V_{mr}K_{mA}[B][C]+V_{mr}[A][B][C]+\frac{V_{mf}K_{iR}K_{mQ}[P]}{K_{eq}}+\frac{V_{mf}K_{iQ}K_{mP}[R]}{K_{eq}}+\frac{V_{mf}K_{mR}[P][Q]}{K_{eq}} \\ +\frac{V_{mf}K_{mQ}[P][R]}{K_{eq}}+\frac{V_{mf}K_{mP}[Q][R]}{K_{eq}}+\frac{V_{mf}[P][Q][R]}{K_{eq}}+\frac{V_{mf}K_{mQ}K_{iR}[A][P]}{K_{eq}} \\ +\frac{V_{mr}K_{iQ}K_{mB}[C][R]}{K_{iR}}+\frac{V_{mf}K_{mQ}K_{iR}[A][B][C]}{K_{iA}K_{iB}K_{eq}}+\frac{V_{mr}K_{mA}[B][C][R]}{K_{iR}}+\frac{V_{mf}K_{mR}[A][P][Q]}{K_{iA}K_{eq}} \\ +\frac{V_{mr}K_{iA}K_{mB}[C][Q][R]}{K_{iQ}K_{iR}}+\frac{V_{mf}K_{iR}K_{mQ}[A][B][C][P]}{K_{iA}K_{iB}K_{iC}K_{eq}}+\frac{V_{mf}K_{iP}K_{mR}[A][B][C][Q]}{K_{iA}K_{iB}K_{iC}K_{eq}} \\ +\frac{V_{mf}K_{mR}[A][B][P][Q]}{K_{iA}K_{iB}K_{eq}}+\frac{V_{mf}K_{mA}[B][C][Q][R]}{K_{iQ}K_{iR}}+\frac{V_{mr}K_{mA}K_{iC}[B][P][Q][R]}{K_{iP}K_{iQ}K_{iR}} \\ +\frac{V_{mr}K_{iA}K_{mB}[C][P][Q][R]}{K_{iP}K_{iQ}K_{iR}}+\frac{V_{mf}K_{mA}[A][B][C][P][Q]}{K_{iA}K_{iB}K_{iC}K_{eq}}+\frac{V_{mf}K_{mA}[B][C][P][Q][R]}{K_{iP}K_{iQ}K_{iR}} \end{matrix}\right]}$$

where the reverse rate is defined using the Haldane constraint as

$V_{mr}=\frac{V_{mf}K_{mP}K_{iQ}K_{iR}}{K_{iA}K_{iB}K_{mC}K_{eq}}$.

**Table S3.6.** Succinyl Coenzyme-A Synthetase Parameters

| Parameter | Definition | Value | Reference |
| --- | --- | --- | --- |
| *V_mf_* | Maximum forward reaction rate (T=25°C) | 3.93x10^4^ nmol/min/mg | a |
| $\Delta_{r}G_{SCoAS}^{0}$ | Gibbs free energy of reaction | 47.62 kJ/mol | [1] |
| *K_mA_* | GDP binding constant | 16.0x10^-6^ M | [1] |
| *K_mB_* | SCoA binding constant | 55.0x10^-6^ M | [1] |
| *K_mC_* | Pi binding constant | 660x10^-6^ M | [1] |
| *K_iA_* | GDP inhibition constant | 5.50x10^-6^ M | [1] |
| *K_iB_* | SCoA inhibition constant | 100x10^-6^ M | [1] |
| *K_iC_* | Pi inhibition constant | 2.00x10^-3^ M | [1] |
| *K_mP_* | CoASH binding constant | 20.0x10^-6^ M | [1] |
| *K_mQ_* | SUC binding constant | 880x10^-6^ M | [1] |
| *K_mR_* | GTP binding constant | 11.1x10^-6^ M | [1] |
| *K_iP_* | CoASH inhibition constant | 20.0x10^-6^ M | [1] |
| *K_iQ_* | SUC inhibition constant | 3.00x10^-3^ M | [1] |
| *K_iR_* | GTP inhibition constant | 11.1x10^-6^ M | [1] |

^a^ Fit to simulation using the experimental data sets described in the main paper.

**Succinate dehydrogenase**

Succinate dehydrogenase (SDH) is a membrane bound enzyme responsible for the oxidation of succinate forming fumarate and reducing equivalents (ubiquinol) for the ETS. We used the reaction mechanism, binding constants and free energies from Wu et al. [1]. The parameter values are located in Table S3.7. For completeness, the rate expression from Wu et al. is presented below.

The reference reaction for SDH is

$[SUC^{2-}]_{mtx}+[UQ]_{mtx}=[UQH_{2}]_{mtx}+[FUM^{2-}]_{mtx}$,

the biochemical equation is

$$[SUC]_{mtx}+[UQ]_{mtx}=[UQH_{2}]_{mtx}+[FUM]_{mtx}$$

and to simplify the notation, the reactants are redefined as follows: [A] = [SUC]_mtx_, [B] = [UQ]_mtx_, [P] = [UQH_2_]_mtx_ and [Q] = [FUM]_mtx_.

The apparent equilibrium constant for this reaction is defined as

$K_{eq}=e^{-\Delta_{r}G_{SDH}^{0}/RT}\left(\frac{P_{FUM}P_{UQH_{2}}}{P_{SUC}P_{UQ}}\right)$.

The overall rate expression for SDH used in the model is

$$J_{SDH}=\frac{V_{mf}\left([A][B]-[P][Q]/K_{eq}\right)}{\left[\begin{matrix} V_{mr}K_{iA}K_{mB}\alpha_{i}+V_{mr}K_{mB}[A]+V_{mr}K_{mA}\alpha_{i}[B]+\frac{V_{mf}K_{mQ}\alpha_{i}[P]}{K_{eq}}+\frac{V_{mf}K_{mP}[Q]}{K_{eq}} \\ +V_{mr}[A][B]+\frac{V_{mf}K_{mQ}[A][P]}{K_{eq}K_{iA}}+\frac{V_{mr}K_{mA}[B][Q]}{K_{iQ}}+\frac{V_{mf}[P][Q]}{K_{eq}} \end{matrix}\right]}$$

where the reverse rate is defined using the Haldane constraint as

$$V_{mr}=\frac{V_{mf}K_{iQ}K_{mP}}{K_{eq}K_{iA}K_{mB}}$$

and

$\alpha_{i}=\left(1+\frac{[OAA]_{m}}{K_{iOAA}}+\frac{[SUC]_{m}}{K_{aSUC}}+\frac{[FUM]_{m}}{K_{aFUM}}\right)/\left(1+\frac{[SUC]_{m}}{K_{aSUC}}+\frac{[FUM]_{m}}{K_{aFUM}}\right)$.

**Table S3.7.** Succinate Dehydrogenase Parameters

| Parameter | Definition | Value | Reference |
| --- | --- | --- | --- |
| *V_mf_* | Maximum forward reaction rate (T=25°C) | 7.00x10^3^ nmol/min/mg | a |
| $\Delta_{r}G_{SDH}^{0}$ | Gibbs free energy of reaction | -10.12 kJ/mol | [1] |
| *K_mA_* | SUC binding constant | 467x10^-6^ M | [1] |
| *K_mB_* | UQ binding constant | 480x10^-6^ M | [1] |
| *K_iA_* | SUC inhibition constant | 120x10^-6^ M | [1] |
| *K_mP_* | UQH_2_ binding constant | 2.45x10^-6^ M | [1] |
| *K_mQ_* | FUM binding constant | 1.20x10^-3^ M | [1] |
| *K_iQ_* | FUM inhibition constant | 1.28x10^-3^ M | [1] |
| *K_iOAA_* | OAA inhibition constant | 1.50x10^-6^ M | [1] |
| *K_aSUC_* | SUC activation constant | 450x10^-6^ M | [1] |
| *K_aFUM_* | FUM activation constant | 375x10^-6^ M | [1] |

^a^ Fit to simulation using the experimental data sets described in the main paper.

**Fumarate hydratase**

Fumarate hydratase (FH) is the enzyme responsible for the condensation reaction incorporating a water molecule into fumarate to form malate. We used the reaction mechanism, binding constants and free energies from Wu et al. [1]. The parameter values are located in Table S3.8. For completeness, the rate expression from Wu et al. is presented below.

The reference reaction for FH is defined as

$[FUM^{2-}]_{mtx}+[H_{2}O]=[MAL^{2-}]_{mtx}$,

the biochemical equation is

$$[FUM]_{mtx}+[H_{2}O]=[MAL]_{mtx}$$

and to simplify the notation, the reactants are redefined as follows: [A] = [FUM]_mtx_ and [P] = [MAL]_mtx_.

The apparent equilibrium constant for this reaction is defined as

$K_{eq}=e^{-\Delta_{r}G_{SDH}^{0}/RT}\left(\frac{P_{MAL}}{P_{FUM}}\right)$.

The overall rate expression for FH used in the model is

$$J_{FH}=\frac{V_{mf}V_{mr}\left([A]-[P]/K_{eq}\right)}{\left[V_{mr}K_{mA}\alpha_{i}+V_{mr}[A]+\frac{V_{mf}[P]}{K_{eq}}\right]}$$

where the reverse rate is defined using the Haldane constraint as

$$V_{mr}=\frac{V_{mf}K_{iQ}K_{mP}}{K_{eq}K_{iA}K_{mB}}$$

and

$\alpha_{i}=1+\frac{[CIT]_{mtx}}{K_{iCIT}}+\frac{[fATP]_{mtx}}{K_{iATP}}+\frac{[fADP]_{mtx}}{K_{iADP}}+\frac{[fGTP]_{mtx}}{K_{iGTP}}+\frac{[fGDP]_{mtx}}{K_{iGDP}}$.

**Table S3.8.** Fumarate Hydratase Parameters

| Parameter | Definition | Value | Reference |
| --- | --- | --- | --- |
| *V_mf_* | Maximum forward reaction rate (T=25°C) | 7.67x10^5^ nmol/min/mg | a |
| $\Delta_{r}G_{FH}^{0}$ | Gibbs free energy of reaction | -3.6 kJ/mol | [1] |
| *K_mA_* | FUM binding constant | 44.7x10^-6^ M | [1] |
| *K_mB_* | MAL binding constant | 198x10^-6^ M | [1] |
| *K_iCIT_* | CIT inhibition constant | 3.50x10^-3^ M | [1] |
| *K_iATP_* | ATP inhibition constant | 40.0x10^-6^ M | [1] |
| *K_iADP_* | ADP inhibition constant | 400x10^-6^ M | [1] |
| *K_iGTP_* | GTP inhibition constant | 80.0x10^-6^ M | [1] |
| *K_iGDP_* | GDP inhibition constant | 330x10^-6^ M | [1] |

^a^ Fit to simulation using the experimental data sets described in the main paper.

**Malate dehydrogenase**

Malate dehydrogenase (MDH) is the enzyme responsible for the oxidation of malate to form oxaloacetate and reducing equivalents for the ETS. We used the reaction mechanism and parameters from Wu et al. [1], except for an additional non-essential Pi-activation mechanism. Malate oxidation displays increased activity from divalent anions such as phosphate and sulfate [35]. We limited the divalent anion stimulation to phosphate. The parameter values are located in Table S3.9. The rate expression from Wu et al. as modified to incorporate Pi activaiton is presented below.

The reference reaction for MDH is defined as

$[NAD^{+}]_{mtx}+[MAL^{2-}]_{mtx}=[OAA^{2-}]_{mtx}+[NADH]_{mtx}+[H^{+}]_{mtx}$,

the biochemical equation is

$$[NAD]_{m}+[MAL]_{m}=[OAA]_{m}+[NADH]_{m}$$

and to simplify the notation, the reactants are redefined as follows: [A] = [NAD]_mtx_, [B] = [MAL]_mtx_, [P] = [OAA]_mtx_ and [Q] = [NADH]_mtx_.

Since the reaction catalyzed by the enzyme MDH involves the generation of a proton, the equilibrium constant for this reaction is defined as

$K_{eq}=e^{-\Delta_{r}G_{MDH}^{0}/RT}\frac{1}{[H^{+}]_{mtx}}$.

The overall rate expression for MDH used in the model is

$$J_{MDH}=\frac{V_{mf}V_{mr}\left([A][B]-[P][Q]/K_{eq}\right)\left(\frac{1+\beta_{Pi}[Pi]/K_{Pi}}{1+[Pi]/K_{Pi}}\right)}{\left[\begin{matrix} V_{mr}K_{iA}K_{mB}\alpha_{i}+V_{mr}K_{mB}[A]+V_{mr}K_{mA}\alpha_{i}[B]+\frac{V_{mf}K_{mQ}\alpha_{i}[P]}{K_{eq}}+\frac{V_{mf}K_{mP}[Q]}{K_{eq}}+V_{mr}[A][B] \\ +\frac{V_{mf}K_{mQ}[A][P]}{K_{eq}K_{iA}}+\frac{V_{mf}[P][Q]}{Keq}+\frac{V_{mr}K_{mA}[B][Q]}{K_{iQ}}+\frac{V_{mr}[A][B][P]}{K_{iP}}+\frac{V_{mf}[B][P][Q]}{K_{iB}K_{eq}} \end{matrix}\right]}$$

where the reverse rate is defined using the Haldane constraint as

$$V_{mr}=\frac{V_{mf}K_{mP}K_{iQ}}{K_{eq}K_{iA}K_{mB}}$$

and

$\alpha_{i}=1+\frac{[fATP]_{mtx}}{K_{iATP}}+\frac{[fADP]_{mtx}}{K_{iADP}}+\frac{[fAMP]_{mtx}}{K_{iAMP}}$.

**Table S3.9.** Malate Dehydrogenase Parameters

| Parameter | Definition | Value | Reference |
| --- | --- | --- | --- |
| *V_mf_* | Maximum forward reaction rate (T=25°C) | 965 nmol/min/mg | a |
| $\Delta_{r}G_{MDH}^{0}$ | Gibbs free energy of reaction | 69.13 kJ/mol | [1] |
| *K_mA_* | NAD binding constant | 90.6x10^-6^ M | [1] |
| *K_mB_* | MAL binding constant | 250x10^-6^ M | [1] |
| *K_iA_* | NAD inhibition constant | 279x10^-3^ M | [1] |
| *K_iB_* | MAL inhibition constant | 360x10^-6^ M | [1] |
| *K_mP_* | OAA binding constant | 6.13x10^-6^ M | [1] |
| *K_mQ_* | NADH binding constant | 2.58x10^-6^ M | [1] |
| *K_iP_* | OAA inhibition constant | 5.50x10^-6^ M | [1] |
| *K_iQ_* | NADH inhibition constant | 3.18x10^-6^ M | [1] |
| *K_iATP_* | ATP inhibition constant | 183x10^-6^ M | [1] |
| *K_iADP_* | ADP inhibition constant | 394x10^-6^ M | [1] |
| *K_iAMP_* | AMP inhibition constant | 420.0x10^-6^ M | [1] |
| *K_Pi_* | Pi binding constant | 5.00x10^-3^ M | a |
| *β_Pi_* | Pi activation constant | 57.4 (unitless) | a |

^a^ Fit to simulation using the experimental data sets described in the main paper.

**Nucleoside diphosphokinase**

Nucleoside diphosphokinase (NDK) is the enzyme responsible for the phosphorylation of nucleoside diphosphates from nucleoside triphosphates. We used the reaction mechanism, binding constants and free energies from Wu et al. [1]. The parameter values are located in Table S3.10. For completeness, the rate expression from Wu et al. is presented below.

The reference reaction for NDK is defined as

$[GTP^{4-}]_{mtx}+[ADP^{3-}]_{mtx}=[GDP^{3-}]_{mtx}+[ATP^{4-}]_{mtx}$,

the biochemical equation is

$$[GTP]_{mtx}+[ADP]_{mtx}=[GDP]_{mtx}+[ATP]_{mtx}$$

and to simplify the notation, the reactants are redefined as follows: [A] = [GTP]_mtx_, [B] = [ADP]_mtx_, [P] = [GDP]_mtx_ and [Q] = [ATP]_mtx_.

The equilibrium constant for this reaction is defined as

$K_{eq}=e^{-\Delta_{r}G_{NDK}^{0}/RT}\left(\frac{P_{GDP}P_{ATP}}{P_{GTP}P_{ADP}}\right)=1$.

The overall rate expression for NDK used in the model is

$$J_{NDK}=\frac{V_{mf}V_{mr}\left([A][B]-[P][Q]/K_{eq}\right)/\alpha_{i}}{\left[\begin{matrix} V_{mr}K_{mB}[A]+V_{mr}K_{mA}[B]+\frac{V_{mf}K_{mQ}[P]}{K_{eq}}+\frac{V_{mf}K_{mP}[Q]}{K_{eq}}+V_{mr}[A][B]+\frac{V_{mf}K_{mQ}[A][P]}{K_{eq}K_{iA}} \\ +\frac{V_{mf}[P][Q]}{Keq}+\frac{V_{mr}K_{mA}[B][Q]}{K_{iQ}} \end{matrix}\right]}$$

where the reverse rate is defined using the Haldane constraint as

$$V_{mr}=\frac{V_{mf}K_{mP}K_{iQ}}{K_{eq}K_{iA}K_{mB}}$$

and

$\alpha_{i}=1+\frac{[fAMP]_{mtx}}{K_{iAMP}}$.

**Table S3.10.** Nucleoside Diphosphokinase Parameters

| Parameter | Definition | Value | Reference |
| --- | --- | --- | --- |
| *V_mf_* | Maximum forward reaction rate (T=25°C) | 6.95x10^3^ nmol/min/mg | a |
| $\Delta_{r}G_{NDK}^{0}$ | Gibbs free energy of reaction | 0 kJ/mol | [1] |
| *K_mA_* | GTP binding constant | 111x10^-6^ M | [1] |
| *K_mB_* | ADP binding constant | 100x10^-6^ M | [1] |
| *K_iA_* | GTP inhibition constant | 162x10^-3^ M | [1] |
| *K_iB_* | ADP inhibition constant | 144x10^-6^ M | [1] |
| *K_mP_* | GDP binding constant | 260x10^-6^ M | [1] |
| *K_mQ_* | ATP binding constant | 278x10^-6^ M | [1] |
| *K_iP_* | GDP inhibition constant | 147x10^-6^ M | [1] |
| *K_iQ_* | ATP inhibition constant | 157x10^-6^ M | [1] |
| *K_iAMP_* | AMP inhibition constant | 650x10^-6^ M | [1] |

^a^ Fit to simulation using the experimental data sets described in the main paper.

**Glutamate oxaloacetate transaminase**

Glutamate oxaloacetate transaminase (GOT), or asparate transaminase, is the enzyme responsible for the transfer of an amino group from aspartate to α-ketoglutarate generating glutamate and oxaloacetate. We used the reaction mechanism, binding constants and free energies from Wu et al. [1]. The parameter values are located in Table S3.11. For completeness, the rate expression from Wu et al. is presented below.

The reference reaction for GOT is defined as

$[ASP^{-}]_{mtx}+[\alpha KG^{2-}]_{mtx}=[OAA^{2-}]_{mtx}+[GLU^{-}]_{mtx}$,

the biochemical equation is

$$[ASP]_{mtx}+[\alpha KG]_{mtx}=[OAA]_{mtx}+[GLU]_{mtx}$$

and to simplify the notation, the reactants are redefined as follows: [A] = [ASP]_mtx_, [B] = [αKG]_mtx_, [P] = [OAA]_mtx_ and [Q] = [GLU]_mtx_.

The equilibrium constant for this reaction is defined as

$K_{eq}=e^{-\Delta_{r}G_{GOT}^{0}/RT}\left(\frac{P_{OAA}P_{GLU}}{P_{\alpha KG}P_{ASP}}\right)$.

The overall rate expression for GOT used in the model is

$$J_{GOT}=\frac{V_{mf}V_{mr}\left([A][B]-[P][Q]/K_{eq}\right)}{\left[\begin{matrix} V_{mr}K_{mB}[A]+V_{mr}K_{mA}\alpha_{i}[B]+\frac{V_{mf}K_{mQ}\alpha_{i}[P]}{K_{eq}}+\frac{V_{mf}K_{mP}[Q]}{K_{eq}}+V_{mr}[A][B]+\frac{V_{mf}K_{mQ}[A][P]}{K_{eq}K_{iA}} \\ +\frac{V_{mf}[P][Q]}{Keq}+\frac{V_{mr}K_{mA}[B][Q]}{K_{iQ}} \end{matrix}\right]}$$

where the reverse rate is defined using the Haldane constraint as

$$V_{mr}=\frac{V_{mf}K_{mP}K_{iQ}}{K_{eq}K_{iA}K_{mB}}$$

and

$\alpha_{i}=1+\frac{[\alpha KG]_{mtx}}{K_{i\alpha KG}}$.

**Table S3.11.** Glutamate Oxaloacetate Transaminase Parameters

| Parameter | Definition | Value | Reference |
| --- | --- | --- | --- |
| *V_mf_* | Maximum forward reaction rate (T=25°C) | 1.09x10^6^ nmol/min/mg | a |
| $\Delta_{r}G_{GOT}^{0}$ | Gibbs free energy of reaction | -216.28 kJ/mol | [1] |
| *K_mA_* | ASP binding constant | 3.90x10^-3^ M | [1] |
| *K_mB_* | αKG binding constant | 430x10^-6^ M | [1] |
| *K_iA_* | ASP inhibition constant | 3.48x10^-3^ M | [1] |
| *K_iB_* | αKG inhibition constant | 710x10^-6^ M | [1] |
| *K_mP_* | OAA binding constant | 88.0x10^-6^ M | [1] |
| *K_mQ_* | GLU binding constant | 8.90x10^-3^ M | [1] |
| *K_iP_* | OAA inhibition constant | 50.0x10^-6^ M | [1] |
| *K_iQ_* | GLU inhibition constant | 8.40x10^-3^ M | [1] |
| *K_i_*_αKG_ | αKG inhibition constant | 16.6x10^-6^ M | [1] |

^a^ Fit to simulation using the experimental data sets described in the main paper.

S3B - Mitochondrial Carbon Substrate Transport Reactions

The pyruvate-hydrogen co-transpoter, glutamate-hydrogen co-transporter, tricarboxylate carrier (TCC, citrate-malate exchanger and isocitrate-malate exchanger) and α-ketoglutarate-malate exchanger (OME) fluxes are identical or similar relative to their previously published mechanisms and parameters in [1] except for the activity coefficients and scaling rate units from M/min to nmol/mg/min. The activity coefficients and maximum rates are listed in Table S3.12 Also the passive permeation dynamics of carbon substrates, cations, anions and adenine nucleosides are in their originally published forms [1] except for scaling of the rate units from M/min to nmol/mg/min. Since the OMM is highly permeable to electrolytes, the IMS cation concentrations were set equal to the cytosolic concentrations. For completeness, the above transport rate expressions from Wu et al. are presented below.

Pyruvate-hydrogen co-transporter:

$J_{PYRH}=V_{PYRH}\left([PYR^{-}]_{ims}[H^{+}]_{ims}-[PYR^{-}]_{mtx}[H^{+}]_{mtx}\right)$.

Glutmate-hydrogen co-transporter:

$J_{GLUH}=V_{GLUH}\left([GLU^{-}]_{ims}[H^{+}]_{ims}-[GLU^{-}]_{mtx}[H^{+}]_{mtx}\right)$.

Tricarboxylate carrier (Citrate-malate exchange):

$J_{TCC}^{CITMAL}=V_{TCC}\left([HCIT^{2-}]_{ims}[MAL^{2-}]_{mtx}-[HCIT^{2-}]_{mtx}[MAL^{2-}]_{ims}\right)$.

Tricarboxylate carrier (Isocitrate-malate exchange):

$J_{TCC}^{ISOCMAL}=V_{TCC}\left([HISOC^{2-}]_{ims}[MAL^{2-}]_{mtx}-[HISOC^{2-}]_{mtx}[MAL^{2-}]_{ims}\right)$.

α-Ketoglutarate-malate exchanger (OME):

$$J_{OME}=\frac{V1V2\left([MAL^{2-}]_{mtx}[\alpha KG^{2-}]_{ims}-[MAL^{2-}]_{ims}[\alpha KG^{2-}]_{mtx}\right)}{\left[\begin{matrix} V2K_{MAL,mtx}^{OME}K_{\alpha KG,ims}^{OME}+V2[MAL^{2-}]_{mtx}K_{\alpha KG,ims}^{OME}+V2[\alpha KG^{2-}]_{ims}K_{MAL,mtx}^{OME}+V2[MAL^{2-}]_{mtx}[\alpha KG^{2-}]_{ims} \\ +V1[\alpha KG^{2-}]_{mtx}K_{MAL,ims}^{OME}+V1[MAL^{2-}]_{ims}K_{\alpha KG,mtx}^{OME}+V1[MAL^{2-}]_{ims}[\alpha KG^{2-}]_{mtx} \end{matrix}\right]}$$

where

$V2=\frac{K_{MAL,ims}^{OME}K_{\alpha KG,mtx}^{OME}}{K_{MAL,mtx}^{OME}K_{\alpha KG,ims}^{OME}}$.

Adenine nucleoside permeation rates:

$J_{ATP}=\gamma p_{A}\left([ATP]_{c}-[ATP]_{ims}\right)$.

$J_{ADP}=\gamma p_{A}\left([ADP]_{c}-[ADP]_{ims}\right)$.

$J_{AMP}=\gamma p_{A}\left([AMP]_{c}-[AMP]_{ims}\right)$.

Inorganic phosphate permeation rate:

$J_{Pi}=\gamma p_{Pi}\left([Pi]_{c}-[Pi]_{ims}\right)$.

TCA cycle substrate permeation rates:

$J_{PYR}=\gamma p_{TI}\left([PYR]_{c}-[PYR]_{ims}\right)$,

$J_{CIT}=\gamma p_{TI}\left([CIT]_{c}-[CIT]_{ims}\right)$,

$J_{MAL}=\gamma p_{TI}\left([MAL]_{c}-[MAL]_{ims}\right)$,

$J_{\alpha KG}=\gamma p_{TI}\left([\alpha KG]_{c}-[\alpha KG]_{ims}\right)$,

$J_{SUC}=\gamma p_{TI}\left([SUC]_{c}-[SUC]_{ims}\right)$,

$J_{GLU}=\gamma p_{TI}\left([GLU]_{c}-[GLU]_{ims}\right)$,

$J_{ASP}=\gamma p_{TI}\left([ASP]_{c}-[ASP]_{ims}\right)$.

**Table S3.12.** Inner-Mitochondrial Membrane Transport Activities

| Parameter | Definition | Value | Reference |
| --- | --- | --- | --- |
| *V_PYRH_* | Pyruvate-hydrogen co-transporter activity (T=25°C) | 3.12x10^13^ nmol/M^2^/min/mg | a |
| *V_GLUH_* | Glutamate-hydrogen co-transporter activity (T=25°C) | 9.44x10^10^ nmol/M^2^/min/mg | a |
| *V_TCC_* | Tricarboxylate carrier exchange activity (T=25°C) | 7.13x10^8^ nmol/M^2^/min/mg | a |
| *V_OME_* | α-Ketoglutarate-malate max forward exchange rate (T=25°C) | 8.83x10^3^ nmol/min/mg | a |
| $K_{MAL,ims}^{OME}$ | IMS MAL binding constant | 1.40x10^-3^ M | [1] |
| $K_{MAL,mtx}^{OME}$ | Matrix MAL binding constant | 700x10^-6^ M | [1] |
| $K_{\alpha KG,ims}^{OME}$ | IMS αKG binding constant | 300x10^-6^ M | [1] |
| $K_{\alpha KG,mtx}^{OME}$ | Matrix αKG binding constant | 170x10^-6^ M | [1] |
| *γ* | Outer mitochondrial membrane surface area/mg | 198 cm^2^/mg | [27-28]^b^ |
| *p_A_* | Adenine nucleoside permeability | 5100 µm/min | [1] |
| *P_Pi_* | Pi permeability | 19620 µm/min | [1] |
| *P_TI_* | TCA cycle substrate permeability | 5100 µm/min | [1] |

^a^ Fit to simulation using the experimental data sets described in the main paper.

^b^ Based on isolated, spherical heart mitochondria with a volume of 0.6 μm^3^ [28] and an average mitochondria/mg of 5.75 x10^9^ [27].

**Glutamate-Aspartate Exchanger**

The electrogenic glutamate-aspartate exchanger (GAE) swaps glutamate and a proton with aspartate taking advantage of the mitochondria’s energized state established by the ETS. The enzyme reaction was modeled based on a rapid equilibrium bi-bi mechanism with a third substrate, protons, added to the rate expression. For simplicity, the full random equilibrium ter-ter mechanism was not used to model this reaction. The substrate kinetic parameters were taken from Dierks et al. [36] and the Ca^2+^-activation binding constant was taken from Contreras et al. [37]. The parameter values used for the rate expression are presented in Table S3.13.

Although glutamic acid has been proposed to exchange with aspartate [17], it is highly improbable due to its low pKa (~4.2) [36]. Tischler et al. describe a process where glutamate and a proton are simultaneously exchanged across the inner mitochondrial membrane for aspartate, capitalizing on the energized state of mitochondria generated by the ETS [38]. This is the proposed mechanism we used. The exchanger was previously assumed to be asymmetric in uncoupled mitochondria [17], but found to be completely symmetric in a reconstituted system [36]; therefore, the “irreversible” nature of the exchanger is due to the proton motive force.

The reference reaction for the GAE defined as

$$[ASP^{-}]_{mtx}+[GLU^{-}]_{ims}+[H^{+}]_{ims}=[ASP^{-}]_{ims}+[GLU^{-}]_{mtx}+[H^{+}]_{mtx}$$

and to simplify the notation, the reactants are redefined as follows: [A] = [ASP^-^]_mtx_, [B] = [GLU^-^]_ims_, [Q] = [ASP^-^]_ims_ and [P] = [GLU^-^]_mtx_.

Since the GAE is electrogenic and transports a proton across the inner-mitochondrial membrane, the equilibrium constant for this reaction is defined as

$K_{eq}=e^{-F\Delta\Psi/RT}\left(\frac{[H^{+}]_{ims}}{[H^{+}]_{mtx}}\right)$.

The forward rate is assumed to be proportional to the membrane potential applied across the exchanger as shown experimentally [17] and is

$V_{mf}=V_{o}e^{-F\Delta\Psi/2RT}$.

For the above expression, the 2 in the denominator of the exponent originates from assuming a centered, single, high peak Erying barrier. Thus the energy required to export ASP^-^ is only subjected to one half of the energy barrier similar to the assumption made in the proton and potassium leak rates. The energy to import ASP^-^ (reverse transport) is subjected to the other half of the energy barrier resulting in matching the reported symmetry of substrate transport [36].

The reverse rate is defined by invoking the Haldane constraint so that

.

The rate expression for glutamate-aspartate exchanger used in the model is

$J_{GAE}=\frac{V_{mf}V_{mr}\left([A][B][H^{+}]_{ims}-[P][Q][H^{+}]_{mtx}/K_{eq}\right)\left(\frac{1+\beta_{Ca}[Ca^{2+}]_{ims}/K_{Ca}}{1+[Ca^{2+}]_{ims}/K_{Ca}}\right)}{\left[\begin{matrix} V_{mr}K_{iA}K_{B}K_{H}+V_{r}K_{B}K_{H}[A]+V_{r}K_{A}[B][H^{+}]_{ims}+V_{mr}[A][B][H^{+}]_{ims} \\ +V_{mr}K_{iA}K_{B}[H^{+}]_{ims}+V_{mf}K_{P}[Q][H^{+}]_{mtx}/K_{eq}+V_{mf}K_{Q}[P][H^{+}]_{mtx}/K_{eq} \\ +V_{mf}[P][Q][H^{+}]_{mtx}/K_{eq}+V_{mf}K_{P}K_{iQ}[H^{+}]_{mtx}/Keq \end{matrix}\right]}$.

**Table S3.13.** Glutamate-Aspartate Exchanger Parameters

| Parameter | Definition | Value | Reference |
| --- | --- | --- | --- |
| *V_o_* | Maximum un-stimulated exchange rate  (ΔΨ = 0, [Ca^2+^]_ims_ = 0, T=25°C) | 674 nmol/min/mg | a |
| *K_A_* | Matrix ASP binding constant | 3.00x10^-3^ M | [36] |
| *K_iA_* | Matrix ASP inhibition constant | 49.0 x10^-6^ M | [36] |
| *K_B_* | IMM GLU binding constant | 200 x10^-6^ M | [36] |
| *K_P_* | IMM ASP binding constant | 80.0 x10^-6^ M | [36] |
| *K_iQ_* | Matrix GLU inhibition constant | 1.70 x10^-3^ M | [36] |
| *K_Q_* | Matrix GLU binding constant | 2.80 x10^-3^ M | [36] |
| *K_H_* | Proton binding constant | 10^-6.5^ M | [36] |
| *K_Ca_* | Ca^2+^ binding constant | 345 x10^-9^ M | [37]^b^ |
| *β_Ca_* | Ca^2+^ activation constant | 27.2 (unitless) | a |

^a^ Fit to simulation using the experimental data sets described in the main paper.

^b^ Averaged values for aralar and citrin carrier types in heart tissue.

The GAE rate expression was corroborated using experimental data pertaining to how the apparent K_M_ for GLU increased as pH was decreased and how the maximum exchange rate increased as the membrane potential was increased. Specifically, Figure S3.5A shows that the rate expression is able to reproduce the apparent K_M_ value for external glutamate measured by Dierks et al. using an isolated liposome system and purified enzyme [36], and Figure S3.5B shows the mitochondrial membrane potential’s effect on the apparent maximum exchange rate using isolated mitochondria loaded with aspartate as the experimental system [17]. None of the data presented in Figure S3.5 was used to fit the glutamate-aspartate exchanger rate expression kinetic parameters. The maximum rate in Figure S3.5B was fit by hand.

**Figure S3.5. The derived GAE rate expression is able to reproduce (A) the apparent K_M_ (open circles) for external glutamate, as well as, (B) the mitochondrial membrane potential’s effect on the apparent maximum exchange rate (open circles).** For (A), the rate expression was used to derive a series of curves at different external glutamate and pH levels. From these curves, the concentration of glutamate that yielded half the maximum rate was identified at each pH level to generate the K_M_ versus pH curve. The predicted pH dependence of the external glutamate K_M_ is shown with the data reported by Dierks et al. [36]. For (B), the data from Murphy et al. [17]) shows that the rate expression can reproduce the voltage dependence of the maximum exchange rate. The maximum un-stimulated exchange rate was set to 0.28 nmol/mg/min.

**Dicarboxylate Carrier**

The dicarboxylate carrier (DCC) exchanges TCA cycle intermediates malate, succinate and hydrogen phosphate. The enzyme reaction was modeled based on a rapid equilibrium bi-bi mechanism. The kinetic parameters for exchanger were fit to experimental data from Indiveri et al. [39-41]. The parameter values used for the rate expression are found in Table S3.14.

In the heart, both the MAL/Pi and SUC/Pi exchange occurs [42-43], and the enzyme can exchange either malate or succinate for inorganic phosphate with an approximately equal affinity. While the enzyme’s turnover rates are comparable to reconstituted adenine nucleotide translocase, glutamate-aspartate exchanger and α-ketoglutarate-malate exchanger and lower than reconstituted inorganic phosphate carrier [39], the activity is low in heart tissue, in part, due to the low expression of the transporter [42].

Since the same enzyme catalyzes two different reaction types, the rate expressions for MAL/Pi and SUC/Pi exchange are derived accounting for conservation of the total enzyme activity. This is discussed below.

The reference reaction for the DCC exchanging malate and phosphate is defined as

$$[MAL^{2-}]_{mtx}+[Pi^{2-}]_{ims}=[MAL^{2-}]_{ims}+[Pi^{2-}]_{mtx}$$

and to simplify the notation, the reactants are redefined as follows: [A_1_] = [MAL^2-^]_mtx_, [B] = [Pi^2-^]_ims_, [Q_1_] = [MAL^2-^]_imss_ and [P] = [Pi^2-^]_mtx_.

The reference reaction for the DCC exchanging succinate and phosphate is

$$[SUC^{2-}]_{mtx}+[Pi^{2-}]_{ims}=[SUC^{2-}]_{ims}+[Pi^{2-}]_{mtx}$$

and to simplify the notation, the reactants are redefined as follows: [A_2_] = [SUC^2-^]_mtx_, and [Q_2_] = [SUC^2-^]_ims_.

Since the DCC is non-electrogenic, the equilibrium constant for this reaction is

$K_{eq}=1$.

The rate expression for the DCC used in the model for malate-phosphate is

$$J_{DCC}^{MAL,Pi}=\frac{V_{\max}\left(\frac{[A_{1}][B]}{K_{A1}K_{B}}-\frac{[P][Q_{1}]}{K_{P}K_{Q1}}\right)}{1+\frac{[A_{1}]}{K_{A1}}+\frac{[A_{2}]}{K_{A2}}+\frac{[A_{1}]}{K_{A1}}\frac{[B]}{K_{B}}+\frac{[A_{2}]}{K_{A2}}\frac{[B]}{K_{B}}+\frac{[P]}{K_{P}}+\frac{[Q_{1}]}{K_{Q1}}+\frac{[Q_{2}]}{K_{Q2}}+\frac{[P]}{K_{P}}\frac{[Q_{1}]}{K_{Q1}}+\frac{[P]}{K_{P}}\frac{[Q_{2}]}{K_{Q2}}}$$

and for succinate-phosphate exchange is

$J_{DCC}^{SUC,Pi}=\frac{V_{\max}\left(\frac{[A_{2}][B]}{K_{A2}K_{B}}-\frac{[P][Q_{2}]}{K_{P}K_{Q2}}\right)}{1+\frac{[A_{1}]}{K_{A1}}+\frac{[A_{2}]}{K_{A2}}+\frac{[A_{1}]}{K_{A1}}\frac{[B]}{K_{B}}+\frac{[A_{2}]}{K_{A2}}\frac{[B]}{K_{B}}+\frac{[P]}{K_{P}}+\frac{[Q_{1}]}{K_{Q1}}+\frac{[Q_{2}]}{K_{Q2}}+\frac{[P]}{K_{P}}\frac{[Q_{1}]}{K_{Q1}}+\frac{[P]}{K_{P}}\frac{[Q_{2}]}{K_{Q2}}}$.

**Table S3.14.** Dicarboxylate Carrier Parameters

| Parameter | Definition | Value | Reference |
| --- | --- | --- | --- |
| *V_max_* | Maximum exchange rate (T=25°C) | 8.03x10^3^ nmol/min/mg | a |
| *K_A1_* | Matrix MAL binding constant | 594x10^-6^ M | [39]^b^ |
| *K_Q1_* | IMS MAL binding constant | 594x10^-6^ M | [39]^b^ |
| *K_A2_* | Matrix SUC binding constant | 844 x10^-6^ M | [41]^c^ |
| *K_Q2_* | IMS SUC binding constant | 844 x10^-6^ M | [41]^c^ |
| *K_B_* | IMS Pi binding constant | 1.70x10^-3^ M | [39]^b^ |
| *K_P_* | Matrix Pi binding constant | 1.70x10^-3^ M | [39]^b^ |

^a^ Fit to simulation using the experimental data sets described in the main paper.

^b^ Fit to data presented in the reference as briefly described below.

^c^ Adjusted from reference by considering the biochemical state at which the data was measured.

Figure S3.6 shows the rate expression evaluated with the experimental conditions for MAL/Pi exchange measured by Indiveri et al. using an isolated liposome system and purified enzyme [39-40]. Although the enzyme was purified from rat liver, the difference between the heart and liver isoform is assumed to be negligible [39]. All the data shown in Figure S3.6A was used to find 2 kinetic constants and one maximum exchange rate. The data point (indicated by *) in Figures S3.6B and C was used to find two other maximum exchange rates for the DCC rate expression.

**Figure S3.6. The experimental data from Indiveri et al. was used to fit the dicarboxylate carrier (DCC) parameters** [39]**.** Reconstituted purified rat liver dicarboxylate carrier was used with either MAL or Pi loaded proteoliposomes with exchange being initiated by the addition of the respective counter anion. (A) The exchange rate with either MAL (open circle) or Pi (open square) loaded proteoliposomes is plotted versus the respective counter anion. (B) The steady state exchange of Pi loaded proteoliposomes at different pHs is plotted versus MAL additions. (C) The exchange rate of Pi loaded proteoliposomes is plotted versus MAL additions at pH 7.2 (open circles) and 6.5 (open squares). The asterisks in Figures B and C indicate the point used to determine the maximum exchange rate for each data set.

S3C - Inner-Mitochondrial Membrane Space and Extra-Mitochondrial Reactions

**Adenylate kinase**

The IMS adenylate kinase reaction is responsible for the inter-conversion of two adenine nucleotide diphosphates into one adenine nucleotide triphosphate and one adenine nucleotide monophosphate. This reaction requires the presence of a divalent cation and is turned off when one is absent. We used the reaction mechanism, binding constants and free energies from Wu et al. [1]. For completeness, the rate expression from Wu et al. is presented below.

The reference reaction for adenylate kinase defined as

$$2[ADP^{3-}]_{ims}=[AMP^{2-}]_{ims}+[ATP^{4-}]_{ims}$$

and the apparent equilibrium constant for this reaction is defined as

$$K_{eq}^{AK}=e^{-\Delta_{r}G_{AK}^{0}/RT}$$

where$\Delta_{r}G_{AK}^{0}=2.26$ kJ/mol.

The overall rate expression for the IMS adenylate kinase reaction used in the model is

$J_{AK}=V_{AK}\left(1-[AMP^{2-}]_{ims}[ATP^{4-}]_{ims}/[ADP^{3-}]_{ims}^{2}/K_{eq}^{AK}\right)$.

The activity coefficient (*V_AK_*) is set arbitrarily large to maintain this reaction close to equilibrium without sacrificing numerical stability.

**Hexokinase**

The hexokinase (HK) reaction is the lead-in enzyme for glycolysis. It phosphorylates the six carbon sugar, glucose, to form glucose-6-phosphate. It is commonly used as an “ATP trap” to maintain high state-3 oxygen consumption rates. This is its purpose in the studies performed by LaNoue et al. [44] that were used in parameter fitting. We used the reaction mechanism and free energies from Wu et al. [1]; however, some of the binding constants were adjusted. The enzyme that was used in the experiments was the yeast HK isoenzyme, but some of the kinetic constants used in Wu et al. were those for the human erythrocyte isoenzyme [45]. To maintain experimental consistency, the kinetic constants for the yeast HK isoenzyme were used and approximated from [45] and the online enzyme Brenda database [46]. The parameter values are located in Table S3.15. For completeness, the rate expression from Wu et al. is presented below.

The reference reaction for HK is defined as

$[GLC]_{cyt}+[ATP^{4-}]_{cyt}\rightleftharpoons[G6P^{2-}]_{cyt}+[ADP^{3-}]_{cyt}+[H^{+}]_{cyt}$,

the biochemical equation is

$$[GLC]_{cyt}+[ATP]_{cyt}=[G6P]_{cyt}+[ADP]_{cyt}$$

and to simplify the notation, the reactants are redefined as follows: [A] = [MgATP^2-^]_cyt_, [B] = [GLC]_cyt_, [P] = [G6P]_cyt_ and [Q] = [MgADP^-^]_cyt_.

Since the reaction catalyzed by HK involves the generation of a proton, the equilibrium constant for this reaction is defined as

$K_{eq}=e^{-\Delta_{r}G_{HK}^{0}/RT}\left(\frac{P_{G6P}K_{D}^{MgATP}}{P_{GLC}K_{D}^{MgADP}}\right)\frac{1}{[H^{+}]_{cyt}}$.

The overall rate expression for HK used in the model is

$J_{HK}=\frac{V_{\max}/\left(K_{iB}K_{mA}\right)\left([A][B]-[P][Q]/K_{eq}\right)}{1+\frac{[A]}{K_{iA}}+\frac{[B]}{K_{iB}}+\frac{[A][B]}{K_{iB}K_{mB}}+\frac{[P]}{K_{iP}}+\frac{[Q]}{K_{iQ}}+\frac{[P][Q]}{K_{iQ}K_{mP}}+\frac{[B][P]}{K_{iG6P}K_{iB}}}$.

**Table S3.15** Hexokinase Parameters

| Parameter | Definition | Value | Reference |
| --- | --- | --- | --- |
| *V_max_* | Maximum reaction rate (T=28°C) | 1.20x10^5^ nmol/min/mg | a,b |
| $\Delta_{r}G_{HK}^{0}$ | Gibbs free energy of reaction | 15.38 kJ/mol | [1] |
| *K_mA_* | MgATP binding constant | 0.50x10^-3^ M | [45-46] |
| *K_mB_* | GLC binding constant | 47.0x10^-6^ M | [45-46] |
| *K_iA_* | MgATP inhibition constant | 0.50x10^-3^ M | [45-46] |
| *K_iB_* | GLC inhibition constant | 49.0x10^-6^ M | [45-46] |
| *K_mP_* | G6P binding constant | 49.0x10^-6^ M | [45-46] |
| *K_mQ_* | MgADP binding constant | 0.50x10^-3^ M | [45-46] |
| *K_iP_* | G6P inhibition constant | 49.0x10^-6^ M | [45-46] |
| *K_iQ_* | MgADP inhibition constant | 0.50x10^-3^ M | [45-46] |
| *K_iG6P_* | Allosteric G6P inhibition constant | 85.0x10^-6^ M | [45-46] |

^a^ Fit to simulation using the experimental data sets described in the main paper.

^b^ Since this reaction was only present in the LaNoue data set, a temperature correction was not made.

S3D - ATP, ADP and Pi Reactions

**Adenine nucleotide translocase**

The adenine nucleotide translocase (ANT) is the enzyme responsible for exchanging unchelated ATP and ADP across the mitochondrial inner membrane. Previous models used a ping-pong mechanism that employed a single adenine nucleotide binding site whereby ΔΨ affected only ATP binding [47]. This type of mechanism is inadequate to describe the observed enzyme kinetics [48-49]. Specifically, ANT inhibition studies have identified at least two distinct adenine nucleotide binding sites; therefore, a newer model presented by Metelkin et al. [50] was employed herein. Additional evidence supporting the two binding site hypothesis stems from the fact that the ANT belongs to the same family of anion transporters as the GAE, DCC and PIC, all of which have been previously shown to possess two binding sites. The unique aspect of the Metelkin et al. model include accounting for ΔΨ’s affect on formation of the ternary complex and the individual rate constants of the antiport process. In order to simulate the experimental conditions, the rate equation was slightly modified and the parameters were refit to the original data [47]. All related parameter values are indicated in Table S3.16. The modified rate expression from Metelkin et al. is presented below.

The reference reaction for the ANT defined as

$$[ATP^{4-}]_{mtx}+[ADP^{3-}]_{ims}=[ATP^{4-}]_{ims}+[ADP^{3-}]_{mtx}$$

and to simplify the notation, the reactants are redefined as follows: [A] = [ATP^4-^]_mtx_, [B] = [ADP^3-^]_ims_, [Q] = [ATP^4-^]_ims_ and [P] = [ADP^3-^]_mtx_.

Since ANT is electrogenic, the equilibrium constant for this reaction is defined as

$K_{eq}=e^{-F\Delta\Psi/RT}$.

The rate expression for ANT used in the model is

$$J_{ANT}=V_{mf}\frac{qk_{2}\frac{[A][B]}{K_{ims}^{ADP}}-k_{3}\frac{[P][Q]}{K_{ims}^{ATP}}}{\left(1+\frac{[Q]}{K_{ims}^{ATP}}+\frac{[B]}{K_{ims}^{ADP}}\right)\left(q[P]+[A]\right)}$$

where

$q=\frac{K_{mtx}^{D}}{K_{mtx}^{T}}=\frac{k_{3}K_{ims}^{D}}{k_{2}K_{ims}^{T}}K_{eq}$,

,

,

$$K_{ims}^{ATP}=K_{A}^{0}e^{\left(z_{T}\delta_{T}\frac{F\Delta\Psi}{RT}\right)}$$

and

$K_{ims}^{ADP}=K_{A}^{0}e^{\left(z_{D}\delta_{D}\frac{F\Delta\Psi}{RT}\right)}$.

.

**Table S3.16.** Adenine Nucleotide Translocase Updated Parameters

| Parameter | Definition | Value | Reference |
| --- | --- | --- | --- |
| *V_mf_* | Maximum reaction rate (T=25°C) | 365 nmol/min/mg | a |
| $k_{2}^{0}$ | Normalized forward turnover rate | 1 unitless | [47]^b^ |
| $k_{3}^{0}$ | Normalized reverse turnover rate | 2.27 unitless | [47]^b^ |
| $K_{A}^{0}$ | External ATP or ADP binding constant | 14.9x10^-6^ M | [47]^b^ |
| *a_1_* | Relative displacement of external adenylate | 0.270 | [47]^b^ |
| *a_2_* | Relative displacement of internal adenylate | 0.192 | [47]^b^ |
| *a_3_* | Relative displacement of all enzyme charges | -0.226 | [47]^b^ |
| *δ_T_* | Relative displacement of external ATP binding | 0.0473 | [47]^b^ |
| *δ_D_* | Relative displacement of external ADP binding | -0.0117 | [47]^b^ |

^a^ Fit to simulation using the experimental data sets described in the main paper.

^b^ Refit to data presented in the reference as briefly described below.

The experimental setup by Kramer and Klingenberg was purified ANT reconsitituted in liposomes under various substrate and energetic conditions [47]. To control the electrophoretic driving force, they used K^+^-diffusion potentials and valinomycin to set the ΔΨ to approximately -180 mV (inside negative) or a Na^+^ based buffer when no ΔΨ was desired. In order to include the binding effects of K^+^ and Na^+^ on free ATP and ADP concentrations, the rate equation was slightly modified and reparameterized using the same data set from Kramer and Klingenberg. Figure S3.7 shows that the rate equation presented above was able to reproduce the experimental data better, or as well as, the original rate equation. The parameters in Table S3.16 revealed a few interesting implications regarding the mechanism used to fit the data. First, the external ADP and ATP binding affinity in the absence of an electrophoretic driving force were found to be equal. In order to determine the internal binding site affinities, additional experiments with lower ATP and ADP concentrations need to be carried out. With the current data set, these binding affinities are unidentifiable. Second, the parameters support the experimental conclusion that changes in ΔΨ have a small effect on the binding affinity but a large effect on the transport rates (small *δ_T_* and *δ_D_* relative to *a_1_*, *a_2_* and *a_3_*). Together, these results help give credence to the dimeric hypothesis of ANT function.

**Figure S3.7. The experimental data from Kramer and Klingenberg [47] was used to fit the adenine nucleotide translocase parameters using the mechanism outlined in Metelkin et al. [50].** Kramer and Klingenberg used purified ANT reconstituted in liposomes to study ANT kinetics under different substrate concentrations and electrophoretic driving forces using labeled adenylates and the inhibitor-stop method. ADP uptake was measured as external ADP was varied (A) with ΔΨ = 0 mV and 0 μM ATP (solid, circle), 20 μM ATP (dash, square) or 100 μM ATP (dot, diamond) or (B) with ΔΨ = -180 mV and 0 μM ATP (solid, circle), 100 μM ATP (dash, square) or 400 μM ATP (dot, diamond). Internal ATP and ADP were 5 mM each. The uptake of either ATP or ADP was measured (C) as the external adenylate was varied with the internal adenylate (10 mM) being identical to the external adenylate. Specifically, ATP uptake with ΔΨ = -180 mV (solid, circle), ADP uptake with ΔΨ = 0 mV (dash, square), ADP uptake with ΔΨ = -180 mV (dot, diamond) or ATP uptake with ΔΨ = 0 mV (dash-dot, triangle) was measured. The uptake of either ATP or ADP was measured (D) as the external adenylates (both equal in concentration) were varied with the internal composition of the liposome consisting of 5 mM ATP and 5 mM ADP. Specifically, ATP uptake with ΔΨ = -180 mV (solid, circle), ADP uptake with ΔΨ = 0 mV (dash, square), ATP uptake with ΔΨ = 0 mV (dot, diamond) or ADP uptake with ΔΨ = -180 mV (dash-dot, triangle) was measured. The molecular weight of dimeric ANT (~60 kDa) and a scaling factor (2.99 min^-1^) were used to convert the normalized turnover rates (unitless) to the measured uptake rates (μmol/mg/min).

**F_1_F_O_ ATP synthase**

F_1_F_O_ ATP synthase is the enzyme responsible for the majority of ATP production in mitochondria. The reaction mechanism F_1_F_O_ ATP synthase during either ATP synthesis or ATP hydrolysis mode is still up to debate [51-52], so we have decided to use the phenomenological thermodynamic expression used in the Wu et al. model [1]. This rate expression was necessary in order to adequately reproduce the state 3 Pi-titration membrane potentials observed in the Bose experiments when using the Wu et al. ETS rate expressions. The parameter values used for the rate expression are presented in Table S3.17. For completeness, the rate expression from Wu et al. is presented below.

The exact number of protons that are shuttled through the c subunit ring structure per reaction turnover is debatable [53]. Certainly biodiversity plays an important role in the variations in the observed stoichiometry of protons per ATP synthesized. Assuming the average number of F_O_ c subunits in the enzyme is 12, it would seem an H^+^:ATP stoichiometry of 4:1 is necessary to maintain balance (three ATP per complete revolution of the γ subunit) [54]. Indeed, the exact proton stoichiometry, whether variable or fixed, requires more attention; however, assuming 3, not 4, protons per ATP, the P:O ratios of NADH (10/4) and SUC (6/4) oxidation are more inline with experiment, 2.5 and 1.5, respectively [53]. If 4 protons per ATP were indeed the actual ratios, then the proton pumping capacity of the ETS must be underestimated. This is possible, considering that enough energy exists for Complex IV to pump up to 6 protons versus its assigned 4 [55]. In order to keep the P:O and ETS pumping ratios at the consensus levels, a ratio of 3 protons per ATP transported through the F_O_ portion of the enzyme was employed.

The reference reaction for F_1_F_O_ ATP synthase is

$[ADP^{3-}]_{mtx}+[Pi^{2-}]_{mtx}+[H^{+}]_{mtx}=[ATP^{4-}]_{mtx}+[H_{2}O]$,

the biochemical reaction is

$$[ADP]_{mtx}+[Pi]_{mtx}=[ATP]_{mtx}$$

and to simplify the notation, the reactants are redefined as follows: [A] = [ADP]_mtx_, [B] = [Pi]_mtx_ and [P] = [ATP]_mtx_.

ATP synthesis requires an energy source. This comes from the membrane potential and proton gradient across the IMM established by the ETS. Thus the equilibrium constant is defined as

$K_{eq}=e^{-\left(\Delta_{r}G_{F_{1}F_{O}}^{0}+3F\Delta\Psi/RT\right)}\left(\frac{[H^{+}]_{ims}^{nH}}{[H^{+}]_{mtx}^{nH-1}}\right)\left(\frac{P_{ATP}}{P_{ADP}P_{Pi}}\right)$.

The overall rate expression for the F_1_F_O_ ATP synthase can then be defined as

$J_{F_{1}F_{O}}=V_{F_{1}F_{O}}\left([A][B]K_{eq}-[P]\right)$.

**Table S3.17.** F_1_F_O_ ATP synthase Parameters

| Parameter | Definition | Value | Reference |
| --- | --- | --- | --- |
| *V_F1FO_* | Maximum activity (T=25°C) | 1.91x10^7^ nmol/M/min/mg | a |
| $\Delta_{r}G_{F_{1}F_{O}}^{0}$ | Gibb’s free energy of reaction | -4.51 kJ/mol | [1] |
| *nH* | H^+^:ATP ratio | 3 | [53] |

^a^ Fit to simulation using the experimental data sets described in the main paper.

**Inorganic phosphate carrier**

The inorganic phosphate carrier (PIC) is the primary means Pi enters the matrix. Due to its relatively high activity, the dihydrogen phosphate:hydroxide equilibrium across the IMM is almost always maintained. In the context of proton concentrations the equilibrium becomes [H_2_PO_4_^-^]_mtx_ / [H_2_PO_4_^-^]_cyt_ = [H^+^]_cyt_ / [H^+^]_mtx_. The exchanger is based on a rapid-equilibrium random bi-bi mechanism resulting in ternary complex formation. The reaction mechanism and binding parameters are as in Nguyen et al. [2]. The parameter values are located in Table S3.18. For completeness, the rate expression from Nguyen et al. is presented below.

The reference reaction for the PIC is

$$[Pi^{-}]_{ims}+[OH^{-}]_{mtx}=[Pi^{-}]_{mtx}+[OH^{-}]_{ims}$$

and to simplify the notation, the reactants are redefined as follows: [A] = [Pi^-^]_ims_, [B] = [OH^-^]_mtx_, [P] = [Pi^-^]_ims_ and [Q] = [OH^-^]_mtx_. The hydroxide concentration is approximated using a water dissociation constant, *K_w_,* of 1.8x10^-14^ M^2^ and the proton/hydroxide relationship, [OH^-^] = *K_w_* /[H^+^].

Since the PIC is non-electrogenic, the equilibrium constant for this reaction is

$K_{eq}=1$.

The full rate expression for the inorganic phosphate carrier used in the model is

$J_{PIC}=\frac{V_{\max}\left(\frac{[A][B]}{K_{A}K_{B}}-\frac{[P][Q]}{K_{P}K_{Q}}\right)}{\left[1+\frac{[A]}{K_{A}}+\frac{[B]}{K_{B}}+\frac{[P]}{K_{P}}+\frac{[Q]}{K_{Q}}+\frac{[A][B]}{K_{A}K_{B}}+\frac{[P][Q]}{K_{P}K_{Q}}\right]}$.

**Table S3.18** Inorganic Phosphate Parameters

| Parameter | Definition | Value | Reference |
| --- | --- | --- | --- |
| *V_max_* | Maximum exchange rate (T=25°C) | 7.59x10^7^ nmol/min/mg | a |
| *K_A_* | IMS Pi binding constant | 1.76x10^-3^ M | [2] |
| *K_B_* | Matrix OH^-^ binding constant | 206x10^-9^ M | [2] |
| *K_P_* | Matrix Pi binding constant | 11.2x10^-3^ M | [2] |
| *K_Q_* | IMS OH^-^ binding constant | 32.6x10^-9^ M | [2] |

^a^ Fit to simulation using the experimental data sets described in the main paper.

S3E - Electron Transport System and Proton Leak

The electron transport system was modeled by including each complex involved with mitochondrial respiration; however, G3P-dehydrogenase and β-oxidation are not included in the model formulation. The ETS model equations are based on the thermodynamically balanced rate equations used in Wu et al. [1]. An effective proton translocation stoichiometry of 4, 2, 4 H^+^/2e^-^ for CI, CIII, and CIV, respectively, was used as proposed by Guerra et al. [55].

**NADH-ubiquinone reductase: Complex I**

NADH-ubiquinone reductase transfers a pair of electrons from NADH to ubiquinone [5]. Once ubiquinol is formed, it is free to diffuse through the lipid bilayer landscape where it can pass the electrons onto cytochrome c via ubiquinol-cytochrome-c reductase. Ubiquinone is a lipid soluble vitamin-like compound, and it resides inside the membrane lipid bilayer amongst the phospholipid hydrophobic tails within a volume approximately equal to 0.250 µl/mg [56]. It is assumed that this space is constant and independent from matrix volume. The enzyme pumps four protons into the IMS per two transported electrons. In total, the enzyme ‘consumes’ five protons from the matrix per turnover, one with NADH and an additional four from the matrix space, with the production of one ubiquinol molecule. This results in a net charge movement of four positive charges per reaction turnover. The parameter values used for the rate expression are found in Table S3.19.

The reference reaction for NADH-ubiquinone reductase is

$[UQ]_{mtx}+[NADH]_{mtx}+[H^{+}]_{mtx}=[NAD^{+}]_{mtx}+[UQH_{2}]_{mtx}$.

The reaction equation used to model the reaction is

$$[UQ]_{mtx}+[NADH]_{mtx}+5[H^{+}]_{mtx}=[NAD^{+}]_{mtx}+[UQH_{2}]_{mtx}+4[H^{+}]_{ims}$$

and to simplify the notation, the reactants are redefined as follows: [A] =[UQ]_mtx_, [B] = [NADH]_mtx_, [P] = [NAD]_mtx_ and [Q] = [UQH_2_]_mtx_.

The equilibrium constant is defined as

$K_{eq}=e^{-\left(\Delta_{r}G_{CI}^{0}-4\Delta\Psi\right)/RT}\left(\left[H^{+}\right]_{mtx}^{5}/\left[H^{+}\right]_{ims}^{4}\right)$.

and the overall reaction rate is defined by

$J_{CI}=V_{C1}\left(K_{eq}AB-PQ\right)$.

**Table S3.19.** NADH-Ubiquinone Reductase Parameters

| Parameter | Definition | Value | Reference |
| --- | --- | --- | --- |
| *V_CI_* | Complex I activity coefficient (T=25°C) | 5.63x10^6^ nmol/M^2^/min/mg | a |
| $\Delta_{r}G_{CI}^{0}$ | Gibb’s free energy of reaction | -118.45 kJ/mol | [1]^b^ |

^a^ Fit to simulation using the experimental data sets described in the main paper.

^b^ The formation energy at T = 298.15 and I = 0.17 M for UQH_2_ was adjusted to -32.07 kJ/mol so that the midpoint potential equals 90 mV [5-6].

**Ubiquinol-cytochrome-c reductase: Complex III**

Ubiquinol-cytochrome-c reductase transfers the pair of electrons from ubiquinol to cytochrome c one at a time requiring two ubiquinol molecules per complete turnover in a process that is known as the Q-cycle. In order to complete one turnover of the Q-cycle, both ubiquinol and ubiquinone must bind to the enzyme at different locations [6]. This results in two electron transport pathways through the enzyme forming a high-potential chain (cytochrome c reduction) and a low-potential chain (ubiquinol regeneration). Although the enzyme has been reported to operate at maximal rates with the Q-pool half reduced, it has been observed that the ubiquinone binding site is always effectively saturated with ubiquinone [57].

Although two ubiquinol molecules are consumed per two electrons passed through the complex, one is regenerated from the low-potential chain, thus only one net ubiquinol is consumed per two cytochrome c molecules reduced. A total of four protons appear in the IMS per two electrons, but only an equivalent of two protons is vectorally transferred. The other two protons are scalar IMS protons that are released by ubiquinol while two matrix protons are picked up by ubiquinone. The two electrons transferred from the positive side to the negative side of the membrane as the two matrix protons are picked up results in only two net charges being displaced from the matrix. The parameter values used for the rate expression are found in Table S3.20.

The reference reaction for ubiquinol-cytochrome-c reductase is

$2[UQH_{2}]_{mtx}+2[c^{3+}]_{ims}+[UQ]_{mtx}=2[UQ]_{mtx}+2[c^{2+}]_{ims}+[UQH_{2}]_{mtx}+2[H^{+}]_{ims}$,

but it is most often presented in reduced form as

$[UQH_{2}]_{mtx}+2[c^{3+}]_{ims}=[UQ]_{mtx}+2[c^{2+}]_{ims}+2[H^{+}]_{ims}$.

The reaction equation used to model the reaction is

$$[UQH_{2}]_{mtx}+2[c^{3+}]_{ims}+2[H^{+}]_{mtx}=[UQ]_{mtx}+2[c^{2+}]_{ims}+4[H^{+}]_{ims}$$

and to simplify the notation, the reactants are redefined as follows: [A] = [UQH_2_]_mtx_, [B] = [c^3+^]_ims_, [P] = [UQ]_mtx_ and [Q] = [c^2+^]_ims_.

The equilibrium constant is defined as

$K_{eq}=e^{-\left(\Delta_{r}G_{C3}^{0}-2\Delta\Psi\right)/RT}\left(\left[H^{+}\right]_{mtx}^{2}/\left[H^{+}\right]_{ims}^{4}\right)$.

Ubiquinol-cytochrome-c reductase is assumed to be modulated by Pi [58]; this Pi stimulation was incorporated by assuming rapid equilibrium between Pi and the enzyme defined as

$a_{Pi}=\frac{\left(1+\beta_{Pi}[Pi^{2-}]_{mtx}/K_{Pi}\right)}{\left(1+[Pi^{2-}]_{mtx}/K_{Pi}\right)}$.

The overall reaction rate is defined by

$J_{C3}=V_{C3}a_{Pi}\left(K_{eq}^{1/2}A^{1/2}B-P^{1/2}Q\right)$.

**Table S3.20.** Ubiquinol-Cytochrome-c Reductase Parameters

| Parameter | Definition | Value | Reference |
| --- | --- | --- | --- |
| *V_C3_* | Complex III activity coefficient (T=25°C) | 5.84x10^5^ nmol/M^3/2^/min/mg | a |
| $\Delta_{r}G_{C3}^{0}$ | Gibb’s free energy of reaction | 55.46 kJ/mol | [1]^b^ |
| *K_Pi_* | Pi binding constant | 4.40x10^-3^ M | a |
| *β_Pi_* | Pi activation constant | 148 unitless | a |

^a^ Fit to simulation using the experimental data sets described in the main paper.

^b^ Note that $\Delta_{f}G_{UQH_{2}}^{0}$ was adjusted to -32.07 kJ/mol.

**Cytochrome-c oxidase: Complex IV**

Cytochrome-c oxidase involves electron transfer from cytochrome c to oxygen via cytochrome a and cytochrome a_3_ in which proton pumping is accompanied by O_2_ reduction [59]. Four cytochrome c molecules pass one electron each to molecular oxygen to produce two water molecules releasing energy. The dependence on ΔΨ and [c^2+^]/([c^2+^]+[c^3+^]) for the rate equation were found to help fit the Bose experimental data set [60]. The parameter values used for the rate expression are found in Table S3.21.

The reference reaction for cytochrome-c oxidase is

$2[c^{2+}]_{ims}+\frac{1}{2}[O_{2}]+2[H^{+}]_{mtx}=2[c^{3+}]_{ims}+[H_{2}O]$.

The reaction equation used to model the reaction is

$$2[c^{2+}]_{ims}+\frac{1}{2}[O_{2}]+4[H^{+}]_{mtx}=2[c^{3+}]_{ims}+[H_{2}O]+2[H^{+}]_{ims}$$

and to simplify the notation, the reactants are redefined as follows: [A] = [c^2+^]_ims_, [B] = [O_2_] and [P] = [c^3+^]_ims_.

The equilibrium constant is defined as

$K_{eq}=e^{-\left(\Delta_{r}G_{C4}^{0}-4\Delta\Psi\right)/RT}\left(\left[H^{+}\right]_{mtx}^{4}/\left[H^{+}\right]_{ims}^{2}\right)$.

The overall reaction rate is defined by

$J_{C4}=V_{C4}\left(\frac{e^{\frac{F\Delta\Psi}{RT}}}{1+K_{B}/B}\right)\left(\frac{A}{A+P}\right)\left(K_{eq}^{1/2}AB^{1/4}-P\right)$.

**Table S3.21.** Cytochrome-c Oxidase Parameters

| Parameter | Definition | Value | Reference |
| --- | --- | --- | --- |
| *V_C4_* | Complex IV activity coefficient (T=25°C) | 44.0 nmol/M/min/mg | a |
| $\Delta_{r}G_{C4}^{0}$ | Gibb’s free energy of reaction | -202.16 kJ/mol | [1] |
| *K_B_* | O_2_ binding constant | 120x10^-6^ M | [1] |

^a^ Fit to simulation using the experimental data sets described in the main paper.

**Proton leak**

The electorphoretically driven proton uptake via leak pathways is a highly non-linear function of the electrical driving force [61]. This was modeled by applying the Goldman constant field assumption, ignoring surface potentials and invoking the Boltzmann function to describe how the energy barriers associated with ion permeation across insulating membranes. We used a single, centered Erying barrier with a high peak to represent the energy barrier for ion translocation. The proton leak permeability, P_mHleak_, was set to 8.06x10^7^ nmol/M/min/mg (T=25°C) in the model.

$$J_{mHleak}=P_{H}^{mHleak}\left([H^{+}]_{ims}e^{-\left(\frac{F\Delta\Psi}{2RT}\right)}-[H^{+}]_{mtx}e^{\left(\frac{F\Delta\Psi}{2RT}\right)}\right)$$

S3F - Potassium Cycle Reactions

The ‘futile’ K cycle plays a major role in mitochondrial volume homeostasis [62-65]. The balance of K^+^ influx via the ATP-dependent potassium channel and electrophoretically driven K^+^ uptake via leak pathways must be balanced with K^+^ efflux from the potassium-hydrogen exchanger.

**Potassium-hydrogen exchanger**

The potassium-hydrogen exchanger (KHE) was modeled based on a rapid equilibrium ordered bi-bi mechanism with proton-binding being the first and last binding event for forward and reverse exchange, respectively. Proton mixed-type inhibition was included in the rate expression using the rapid equilibrium method. The kinetic parameters for the exchanger were fit to experimental data from Brieley and Jung [66]. The max flux of the K^+^/H^+^ exchanger does not exceed 10% of the maximum proton efflux during respiration [65] and under ADP- and Pi-free conditions is strongly inhibited when Mg^2+^ is present.

The KHE was hypothesized to be regulated via the carrier brake hypothesis [67], but Brierley and Jung call into question the relevance of this exchanger under physiological conditions due to the strong inhibitory environment present [66]. Garlid then proposed that the K^+^/H^+^ exchanger is regulated by matrix volume by membrane stretching activating the exchanger [68]. Garlid’s regulation mechanism is interpreted as a volume sensing portion of the exchanger that serves as an activating mechanism as the volume increases. This has an effect of activating the exchanger even in the presence of a considerable amount of endogenous inhibitory agents. A linear relationship between the exchanger rate and matrix volume was found to best reproduce the experimental data. See the main paper for details. The parameter values used for the rate expression are found in Table S3.22.

The reference reaction for the KHE is

$$[H^{+}]_{ims}+[K^{+}]_{mtx}=[K^{+}]_{ims}+[H^{+}]_{mtx}$$

and to simplify the notation, the reactants are redefined as follows: [A] = [H^+^]_ims_, [B] = [K^+^]_mtx_, [P] = [K^+^]_ims_ and [Q] = [H^+^]_mtx_.

Since the KHE is non-electrogenic, the equilibrium constant for this reaction is

$K_{eq}=1$.

Before the rate expression for the KHE is introduced, inhibition relationships must first be defined. The exchanger exhibits mixed type inhibition with respect IMS protons, and the apparent K_M_ for both matrix and IMS K^+^ is controlled by matrix and IMS protons, respectively. Using the rapid equilibrium assumption, the apparent K_M_ inhibitory factors are defined below as

for the matrix side and

$$a_{iP}=\left(1+K_{H}/[A]\right)/\left(1+K_{H}/[A]/a_{H}\right)$$

for the inner-mitochondrial membrane side (*K_H_* is identical to *K_iA_* defined in Table S3.22). The exchange rate is inhibited by matrix Mg^2+^ and Ca^2+^ as well as H^+^ in the IMS and activated by an increase in matrix volume. These regulatory actions are defined as

$a_{i}=\left(\frac{1+\beta_{H}K_{H}/[A]/\alpha_{H}}{1+K_{H}/[A]/\alpha_{H}}\right)\left(\frac{V_{mtx}}{[Mg^{2+}]_{mtx}/K_{iMg}+[Ca^{2+}]_{mtx}/K_{iCa}}\right)$.

The full rate expression for the KHE used in the model is

$$J_{KHE}=\left(\frac{V_{\max}\left(\left[A\right]\left[B\right]-\left[P\right]\left[Q\right]/Keq\right)a_{i}}{\left[K_{iA}K_{B}a_{iB}+_{r}\left[A\right]K_{B}a_{iB}+\left[A\right]\left[B\right]+\left[Q\right]K_{P}a_{iP}/K_{eq}+\left[P\right]\left[Q\right]/K_{eq}\right]}\right)$$

**Table S3.22.** Potassium-Hydrogen Exchanger Parameters

| Parameter | Definition | Value | Reference |
| --- | --- | --- | --- |
| *V_max_* | Maximum exchange rate (T=25°C) | 6.25 nmol/nl/min/mg | a |
| *K_iA_* | H^+^ binding constant | 1.63x10^-10^ M | [66]^b^ |
| *K_B_* | Matrix K^+^ binding constant | 1.80x10^-3^ M | [66]^b^ |
| *K_P_* | IMS K^+^ binding constant | 1.80x10^-3^ M | [66]^b^ |
| *α_H_* | Proton modulation constant | 28.3 unitless | [66]^b^ |
| *β_H_* | Proton inhibition constant | 1.40 x10^-3^ unitless | [66]^b^ |
| *K_iMg_* | Matrix Mg^2+^ inhibition constant | 92.0x10^-6^ M | [69] |
| *K_iCa_* | Matrix Ca^2+^ inhibition constant | 2.20x10^-6^ M | [69] |

^a^ Fit to simulation using the experimental data sets described in the main paper.

^b^ Fit to data presented in the reference as briefly described below.

Figure S3.8 shows the KHE parameter fits for the data presented by Brieley and Jung [66]. In their work, they depleted isolated mitochondria of divalent cations using A23187 to activate the KHE and measure K^+^ dependent H^+^ ejection at 25°C. We used 32 data points (5 data curves) to find 4 parameters (4 kinetic constants and 3 maximum exchange rates) for the KHE rate expression. Although the maximum exchange rate was allowed to change for each figure, they were all very similar, 111.5 +/- 15.7 nmol/mg/min, and within the reported experimental maximum rate, 113 nmol/mg/min. The matrix volume was not given for each experimental condition, but it was reported to average 1 μl/mg.

**Figure S3.8. The experimental data from Brieley and Jung** [66] **was used to fit the potassium-hydrogen exchanger parameters.** The experimental system used to gather the data was divalent cation depleted isolated mitochondria. The matrix volume that was used for the fits was set to the reported value of 1 ul/mg. The H^+^ efflux was measured for 15 mM (open circles) and 30 mM (open squares) external K^+^ versus matrix H^+^ concentration (A). The H^+^ efflux was also measured at different matrix and external H^+^ concentrations while the external K^+^ concentration was held fixed at 20 mM (B). The pH values next to the data points indicate the external pH while the internal H^+^ concentration is indicated by the abscissa.

**ATP-dependent potassium channel**

The mechanism for K^+^ entry into the matrix is through the ATP-dependent potassium channel (mKATP) and electrophoretically driven K^+^ leak pathways. The ATP-dependent K^+^ channel was characterized using data from Mironova et al. and Garlid et al. [65,70]. The channel was modeled using a thermodynamically balanced expression to relate K^+^ driving forces hybridized with channel kinetics similar to the method used by Dash and Beard [71]. The channel is an inward rectifier and is strongly modulated by nucleotides in the presence of Mg^2+^. Although a model with a single binding site best fit the adenine nucleotide data [72], the rate expression below is slightly modified to represent the complex regulatory processes (adenine nucleotide inhibition and guanidine nucleotide activation) occurring in the mitochondrial matrix. Specifically, for the sole purpose of modeling the volume dynamics, the channel is assumed to be inhibited by MgATP with MgADP acting as an activator by increasing the K_i_ for MgATP. Although the calcium chelated forms of these adenine nucleosides also inhibit the channel, the overall contribution to the channels inhibition is primarily due to the Mg^2+^-chelated form and therefore is not used in the model. The inward rectification requires either a Markov-type state model of the channel or a phenomenological model to reproduce the currents at positive potential. Since the mitochondrial membrane potential is rarely positive during physiological conditions, we did not include this current-voltage relationship in the model expression. The hybrid Goldman-Hodgkin-Katz flux formulism is adequately able to reproduce the current-voltage relationship at negative potentials seen in Figure 3.9A. The parameter values used for the rate expression are found in Table S3.23.

The rate expression for the ATP-dependent potassium channel is

.

**Table S3.23.** ATP-dependent Potassium Channel Parameters

| Parameter | Definition | Value | Reference |
| --- | --- | --- | --- |
| $G_{K}^{mKATP}$ | Potassium conductance (T=25°C) | 6.75 nmol/min/mg | a |
| *β* | Erying energy barrier constant | 0.25 unitless | [70,72] |
| *K_K_* | K^+^ binding constant | 0.032 M | [72] |
| $K_{iMgATP}$ | MgATP inhibition constant | 4.10x10^-9^ M | [72] ^a^ |
| $K_{iMgADP}$ | MgADP inhibition constant | 10.0x10^-6^ M | [72] ^a^ |

^a^ Fit to simulation using the experimental data sets described in the main paper.

Figure S3.9 shows that the simplified expression for ATP-dependent potassium flux can acceptably describe the experimental data. The channel conductance was calibrated to fit the trend in each experimental data set. The 55-kDa inward rectifying mK_ATP_ from rat hepatocytes was characterized using the data from Mironova et al. [70] as shown in Figure S3.9A. They studied the channel using the patch-clamp technique with the isolated channel resuspended in liposomes in symmetric 100 mM KCl. Paucek et al. studied the channel in proteoliposomes using rat hepatocytes and bovine heart sources shown in Figure S3.9B [72]. They used the permeant anion ClO_4_^-^ to establish a membrane potential of approximately 180 mV across the proteoliposome membrane. The qualitative and quantitative difference between liver and heart ATP-dependent potassium channels were assumed to be negligible [73].

**Figure S3.9. The derived ATP-dependent potassium channel rate expression is able to reproduce (A) the current- voltage relationship (open circles), and (B) the K^+^-dependent transport kinetics (open circles).** The ATP-dependent potassium channel current-voltage data was from Mironova et al. [70], and the K^+^-dependent transport kinetic data was from Paucek et al. [72]. None of the data presented in the above figures was used for parameter estimation; the channel conductance for each experimental data set was calibrated by hand.

**Potassium leak**

The electorphoretically driven K^+^ uptake via leak pathways is a highly non-linear function of the electrical driving force [61]. We modeled the K^+^ leak kinetics similarly to the H^+^ leak kinetics. For details, please see the end of section S3E. The K^+^ leak permeability, P_mKleak_, was set to 13.7 nmol/M/min/mg (T=25°C) in the model. The mitochondrial potassium leak rate expression used is

$J_{mKleak}=P_{K}^{mKleak}\left([K^{+}]_{cyt}e^{-\left(\frac{F\Delta\Psi}{2RT}\right)}-[K^{+}]_{mtx}e^{\left(\frac{F\Delta\Psi}{2RT}\right)}\right)$.

S3G - Sodium-Calcium Cycle Reactions

The sodium and calcium dynamics present in the model are based on that presented by Dash and Beard and Nguyen et al. [2,71]. The sigmoid calcium-dependence and non-linear transmembrane voltage-dependence for the calcium uniporter is similar to the expression published by Dash and Beard. The sodium-calcium exchanger is similar to both published expressions. The sodium-hydrogen exchanger is identical to Nguyen et al. with the matrix proton regulation refit to the experimental data.

**Calcium uniporter**

Mitochondrial Ca^2+^ influx primarily occurs via the Ca^2+^ selective ion channel known as the calcium uniporter. The rate expression is based on Dash and Beard [71] and was extended to include a Mg^2+^ non-competitive inhibition mechanism. It was assumed that Mg^2+^ could bind to the 2^nd^ Ca^2+^ binding site and prevent Ca^2+^ transport and modeled as a rapid equilibrium mechanism. The parameter values used for the rate expression are found in Table S3.24.

The rate expression for mitochondrial Ca^2+^ uptake is,

$J_{UNI}=P_{Ca}^{CaUNI}\left(\frac{\frac{-z_{Ca}F\Delta\Psi}{n_{Ca}RT}}{\sinh\left(\frac{-z_{Ca}F\Delta\Psi}{n_{Ca}RT}\right)}\right)^{n_{Ca}}\frac{[Ca^{2+}]_{ims}^{2}e^{-\left(\frac{z_{Ca}\Delta\Psi F}{RT}\right)}-[Ca^{2+}]_{mtx}^{2}e^{\left(\frac{z_{Ca}\Delta\Psi F}{RT}\right)}}{\left[\begin{matrix} K_{Ca}^{2}+K_{Ca}^{2}\frac{[Mg^{2+}]_{ims}}{K_{Mg}}+K_{Ca}^{2}\frac{[Mg^{2+}]_{mtx}}{K_{Mg}}+K_{Ca}[Ca^{2+}]_{ims}\frac{[Mg^{2+}]_{ims}}{K_{Mg}} \\ +K_{Ca}[Ca^{2+}]_{mtx}\frac{[Mg^{2+}]_{mtx}}{K_{Mg}}+[Ca^{2+}]_{ims}^{2}+[Ca^{2+}]_{mtx}^{2} \end{matrix}\right]}$.

**Table S3.24.** Calcium Uniporter Parameters

| Parameter | Definition | Value | Reference |
| --- | --- | --- | --- |
| $P_{Ca}^{CaUNI}$ | Calcium Permeability (T=25°C) | 157 nmol/min/mg | a |
| *K_Ca_* | Calcium binding constant | 4.35x10^-6^ M | [74-77]^b^ |
| *n_Ca_* | Membrane potential constant | 2.40 unitless | [74-77]^b^ |
| *K_Mg_* | Magnesium binding constant | 17.31x10^-6^ M | [74-77]^b^ |
| *a_Mg_* | Magnesium inhibition constant | 10.7 | [74-77]^b^ |

^a^ Fit to simulation using the experimental data sets described in the main paper.

^b^ Fit to data presented in the reference as briefly described below.

Figure S3.10 shows the calcium uniporter rate expression fits the experimental data from four independent data sets [74-77]. It is interesting to note that a single Ca^2+^ binding constant can reproduce the data from different tissues in rat with the Mg^2+^-dependent non-competitive inhibition mechanism. It is entirely plausible to consider the major difference between liver and heart calcium uniporter dynamics be attributed to different expression levels (see Figure S3.10 for details) versus different calcium binding affinities as would be expected in different species; however, more research concerning this matter needs to be done. For each data set the free cation concentration was calculated using the K_D_ values defined in Part S1C. The nonlinear ΔΨ-dependence is conserved from Dash and Beard [71] with the cooperativity parameter, *n_Ca_*, refit to the data. For succinate energized mitochondrial in the presence of rotenone, the ΔΨ was fixed at -160 mV in contrast to the -190 mV employed in Dash and Beard [71]. This change is based on considering the P/O ratio for succinate that is approximately 1.5 while it is approximately 2.5 for NADH-linked substrates [78].

**Figure S3.10. The fit calcium uniporter rate expression (as indicated by Ca^2+^ uptake) is compared to the four independent experimental data sets.** A) Vinogradov and Scarpa spectrophotometrically measured Ca^2+^ uptake using succinate-energized rat liver mitochondria incubated at various extra-mitochondrial [Ca^2+^] in the presence 2 mM MgCl_2_ at 24 ºC [76]. B) Scarpa and Grazzioti also spectrophotometrically measured Ca^2+^ uptake at various extra-mitochondrial [Ca^2+^]; however, they used succinate-energized heart mitochondria in the presence of 5 mM MgCl_2_ at 26 ºC [75]. C) Wingrove et al. used a ruthenium red quench technique with a TPP electrode to measure Ca^2+^ uptake of succinate-energized liver mitochondria versus ΔΨ at 1.5 μM (circles), 1.0 μM (squares) and 0.5 μM (diamonds) extra-mitochondrial [Ca^2+^] at 25 ºC [77]. The ΔΨ was titrated using malonate. D) Bragadin et al. measured Ca^2+^ uptake using K^+^-diffusion potentials (estimated to be -185 mV based on 125 mM K^+^ in the matrix and 100 μM K^+^ in the buffer) and a K^+^-selective electrode correlated with Ca^2+^ uniporter rates at 0 mM (circles), 2 mM (squares) and 5 mM (diamonds) extra-mitochondrial [Mg^2+^] at 20 ºC [74]. The max uptake rates used were 2.08, 2.64, 2.20 +/- 0.18 (n=3) and 1.76 +/- 0.15 (n=3) nmol/mg/min for A, B, C and D, respectively. The matrix Mg^2+^ was estimated for each data set and bounded in the acceptable experimental range of 10 μM to 1 mM and averaged 0.54 mM.

**Sodium-calcium exchanger**

In order to balance Ca^2+^ influx from the calcium uniporter, mitochondria utilize what is known as the sodium-calcium exchanger. The exchanger stoichiometry is assumed to be 3:1 resulting in an electrogenic exchange of three Na^+^ for one Ca^2+^. The expression is based on a rapid equilibrium random bi-bi mechanism and is similar to the Dash and Beard’s expression [71] with additional and Ca^2+^-activation. The Ca^2+^-activation mechanism is present in the sarcolemmal isozyme [79], and we hypothesize that it is present in the mitochondrial isoform as well. The parameter values used for the rate expression are found in Table S3.25.

The rate expression for mitochondrial Na^+^/Ca^2+^ exchange is,

.

**Table S3.25.** Sodium-Calcium Exchanger Parameters

| Parameter | Definition | Value | Reference |
| --- | --- | --- | --- |
| *V_max_* | Maximum exchange rate (T=25°C) | 0.731 nmol/min/mg | a |
| *K_Na_* | Sodium binding constant | 1.70x10^-3^ M | a |
| *K_Ca_* | Calcium binding constant | 4.17x10^-6^ M | a |
| *K_aCa_* | Calcium activation binding constant | 1.66x10^-6^ M | a |
| *β_Ca_* | Calcium activation constant | 67.6 | a |

^a^ Fit to simulation using the experimental data sets described in the main paper.

**Sodium-hydrogen exchanger**

The balance of sodium is achieved via the sodium-hydrogen exchanger. This exchanger is modeled similarly to sodium-calcium exchanger in that it assumes a rapid equilibrium random bi-bi mechanism except for an additional dependence on matrix pH as in Nguyen et al. [2]. The proton regulatory binding constant was refit to the data presented in Kapus et al. [80]. This rate was modified slightly since a better fit to the data was achieved by including a Hill coefficient of 2 in the regulatory binding term. The parameter values used for the rate expression are found in Table S3.26.

The rate expression for mitochondrial Na^+^/H^+^ exchange is,

$J_{NHE}=V_{\max}\frac{[H^{+}]_{mtx}^{2}}{[H^{+}]_{mtx}^{2}+K_{H,reg}^{2}}\frac{\frac{[H^{+}]_{ims}}{K_{H}}\frac{[Na^{+}]_{mtx}}{K_{Na}}-\frac{[H^{+}]_{mtx}}{K_{H}}\frac{[Na^{+}]_{ims}}{K_{Na}}}{\left[\begin{matrix} 1+\frac{[H^{+}]_{ims}}{K_{H}}+\frac{[Na^{+}]_{mtx}}{K_{Na}}+\frac{[H^{+}]_{ims}}{K_{H}}\frac{[Na^{+}]_{mtx}}{K_{Na}}+ \\ \frac{[H^{+}]_{mtx}}{K_{H}}+\frac{[Na^{+}]_{ims}}{K_{Na}}+\frac{[H^{+}]_{mtx}}{K_{H}}\frac{[Na^{+}]_{ims}}{K_{Na}} \end{matrix}\right]}$.

**Table S3.26.** Sodium-Hydrogen Exchanger Parameters

| Parameter | Definition | Value | Reference |
| --- | --- | --- | --- |
| *V_max_* | Maximum exchange rate (T=25°C) | 9.45x10^5^ nmol/min/mg | a |
| *K_Na_* | Sodium binding constant | 24.25x10^-3^ M | [2] |
| *K_H_* | Proton binding constant | 10^-8.5^ M | [2] |
| *K_H,reg_* | Proton regulatory binding constant | 10^-7.2^ M | [80]^b^ |

^a^ Fit to simulation using the experimental data sets described in the main paper.

^b^ Fit to data presented in the reference.

References

1. Wu F, Yang F, Vinnakota KC, Beard DA (2007) Computer modeling of mitochondrial tricarboxylic acid cycle, oxidative phosphorylation, metabolite transport, and electrophysiology. J Biol Chem 282: 24525-24537.

2. Nguyen MH, Dudycha SJ, Jafri MS (2007) Effect of Ca2+ on cardiac mitochondrial energy production is modulated by Na+ and H+ dynamics. Am J Physiol Cell Physiol 292: C2004-2020.

3. Cortassa S, Aon MA, O'Rourke B, Jacques R, Tseng HJ, et al. (2006) A computational model integrating electrophysiology, contraction, and mitochondrial bioenergetics in the ventricular myocyte. Biophys J 91: 1564-1589.

4. Korzeniewski B, Zoladz JA (2001) A model of oxidative phosphorylation in mammalian skeletal muscle. Biophys Chem 92: 17-34.

5. Brandt U (2006) Energy Converting NADH:Quinone Oxidoreductase (Complex I). Annu Rev Biochem.

6. Crofts AR (2004) The cytochrome bc1 complex: function in the context of structure. Annu Rev Physiol 66: 689-733.

7. Smith RM, Martell AE, Chen Y (1991) Critical Evaluation of Stability Constants for Nucleotide Compelxes with Protons and Metal Ions and the Accompanying Enthalpy Changes. Pure and Applied Chemistry 63: 1015-1080.

8. Smith RM, Alberty RA (1956) The Apparent Stability Constants of Ionic Complexes of Various Adenosine Phosphates with Monovalent Cations. Journal of Physical Chemistry 60: 180-184.

9. O'Sullivan WJ, Smithers GW (1979) Stability constants for biologically important metal-ligand complexes. Methods Enzymol 63: 294-336.

10. Beard DA, Vinnakota KC, Wu F (2008) Detailed enzyme kinetics in terms of biochemical species: study of citrate synthase. PLoS ONE 3: e1825.

11. Qi F, Chen XW, Beard DA (2008) Detailed kinetics and regulation of mammalian NAD-linked isocitrate dehydrogenase. Biochimica Et Biophysica Acta-Proteins and Proteomics 1784: 1641-1651.

12. Vinnakota KC, Wu F, Kushmerick MJ, Beard DA (2009) Multiple ion binding equilibria, reaction kinetics, and thermodynamics in dynamic models of biochemical pathways. Methods Enzymol 454: 29-68.

13. Bittar EE, Bittar N (1995) Cell chemistry and physiology. Greenwich, Conn.: JAI Press. v <1-4 > p.

14. Yang W, Drueckhammer DG (2003) Computational study of the citrate synthase catalyzed deprotonation of acetyl-coenzyme a and fluoroacetyl-coenzyme A: Demonstration of a layered quantum mechanical approach. Journal of Physical Chemistry B 107: 5986-5994.

15. Kearney EB, Ackrell BAC, Mayr M, Singer TP (1974) Studies on Succinate-Dehydrogenase .23. Activation of Succinate-Dehydrogenase by Anions and Ph. Journal of Biological Chemistry 249: 2016-2020.

16. Ragan CI, Heron C (1978) Interaction between Mitochondrial Nadh-Ubiquinone Oxidoreductase and Ubiquinol-Cytochrome-C Oxidoreductase - Evidence for Stoiciometric Association. Biochemical Journal 174: 783-790.

17. Murphy E, Coll KE, Viale RO, Tischler ME, Williamson JR (1979) Kinetics and regulation of the glutamate-aspartate translocator in rat liver mitochondria. J Biol Chem 254: 8369-8376.

18. Shertzer HG, Racker E (1976) Reconstitution and Characterization of Adenine-Nucleotide Transporter Derived from Bovine Heart-Mitochondria. Journal of Biological Chemistry 251: 2446-2452.

19. Ligeti E, Brandolin G, Dupont Y, Vignais PV (1985) Kinetics of Pi-Pi Exchange in Rat-Liver Mitochondria - Rapid Filtration Experiments in the Millisecond Time Range. Biochemistry 24: 4423-4428.

20. Magnus G, Keizer J (1997) Minimal model of beta-cell mitochondrial Ca2+ handling. Am J Physiol 273: C717-733.

21. Lass A, Agarwal S, Sohal RS (1997) Mitochondrial ubiquinone homologues, superoxide radical generation, and longevity in different mammalian species. J Biol Chem 272: 19199-19204.

22. Morkuniene R, Arandarcikaite O, Borutaite V (2006) Estradiol prevents release of cytochrome c from mitochondria and inhibits ischemia-induced apoptosis in perfused heart. Exp Gerontol 41: 704-708.

23. Asimakis GK, Sordahl LA (1981) Intramitochondrial adenine nucleotides and energy-linked functions of heart mitochondria. Am J Physiol 241: H672-678.

24. Jung DW, Panzeter E, Baysal K, Brierley GP (1997) On the relationship between matrix free Mg2+ concentration and total Mg2+ in heart mitochondria. Biochim Biophys Acta 1320: 310-320.

25. Corkey BE, Duszynski J, Rich TL, Matschinsky B, Williamson JR (1986) Regulation of free and bound magnesium in rat hepatocytes and isolated mitochondria. J Biol Chem 261: 2567-2574.

26. Alberty RA (2003) Thermodynamics of biochemical reactions. Hoboken, N.J.: Wiley-Interscience. ix, 397 p. p.

27. Beavis AD, Brannan RD, Garlid KD (1985) Swelling and contraction of the mitochondrial matrix. I. A structural interpretation of the relationship between light scattering and matrix volume. J Biol Chem 260: 13424-13433.

28. Gear AR, Bednarek JM (1972) Direct counting and sizing of mitochondria in solution. J Cell Biol 54: 325-345.

29. Kowaltowski AJ, Seetharaman S, Paucek P, Garlid KD (2001) Bioenergetic consequences of opening the ATP-sensitive K(+) channel of heart mitochondria. Am J Physiol Heart Circ Physiol 280: H649-657.

30. Hucho F, Randall DD, Roche TE, Burgett MW, Pelley JW, et al. (1972) -Keto acid dehydrogenase complexes. XVII. Kinetic and regulatory properties of pyruvate dehydrogenase kinase and pyruvate dehydrogenase phosphatase from bovine kidney and heart. Arch Biochem Biophys 151: 328-340.

31. Mcminn CL, Ottaway JH (1977) Studies on Mechanism and Kinetics of 2-Oxoglutarate Dehydrogenase System from Pig Heart. Biochemical Journal 161: 569-581.

32. Panov A, Scarpa A (1996) Independent modulation of the activity of alpha-ketoglutarate dehydrogenase complex by Ca2+ and Mg2+. Biochemistry 35: 427-432.

33. Mccormack JG, Denton RM (1979) Effects of Calcium-Ions and Adenine-Nucleotides on the Activity of Pig-Heart 2-Oxoglutarate Dehydrogenase Complex. Biochemical Journal 180: 533-544.

34. Rodriguez-Zavala JS, Pardo JP, Moreno-Sanchez R (2000) Modulation of 2-oxoglutarate dehydrogenase complex by inorganic phosphate, Mg(2+), and other effectors. Arch Biochem Biophys 379: 78-84.

35. Telegdi M, Wolfe DV, Wolfe RG (1973) Malate dehydrogenase. XII. Initial rate kinetic studies of substrate activation of porcine mitochondrial enzyme by malate. J Biol Chem 248: 6484-6489.

36. Dierks T, Riemer E, Kramer R (1988) Reaction mechanism of the reconstituted aspartate/glutamate carrier from bovine heart mitochondria. Biochim Biophys Acta 943: 231-244.

37. Contreras L, Gomez-Puertas P, Iijima M, Kobayashi K, Saheki T, et al. (2007) Ca2+ Activation kinetics of the two aspartate-glutamate mitochondrial carriers, aralar and citrin: role in the heart malate-aspartate NADH shuttle. J Biol Chem 282: 7098-7106.

38. Tischler ME, Pachence J, Williamson JR, La Noue KF (1976) Mechanism of glutamate-aspartate translocation across the mitochondrial inner membrane. Arch Biochem Biophys 173: 448-461.

39. Indiveri C, Capobianco L, Kramer R, Palmieri F (1989) Kinetics of the reconstituted dicarboxylate carrier from rat liver mitochondria. Biochim Biophys Acta 977: 187-193.

40. Indiveri C, Dierks T, Kramer R, Palmieri F (1989) Kinetic discrimination of two substrate binding sites of the reconstituted dicarboxylate carrier from rat liver mitochondria. Biochim Biophys Acta 977: 194-199.

41. Palmieri F, Prezioso G, Quagliariello E, Klingenberg M (1971) Kinetic study of the dicarboxylate carrier in rat liver mitochondria. Eur J Biochem 22: 66-74.

42. Saint-Macary M, Foucher B (1985) Comparative partial purification of the active dicarboxylate transport system of rat liver, kidney and heart mitochondria. Biochem Biophys Res Commun 133: 498-504.

43. Szewczyk A, Nalecz MJ, Broger C, Wojtczak L, Azzi A (1987) Purification by affinity chromatography of the dicarboxylate carrier from bovine heart mitochondria. Biochim Biophys Acta 894: 252-260.

44. LaNoue KF, Bryla J, Williamson JR (1972) Feedback interactions in the control of citric acid cycle activity in rat heart mitochondria. J Biol Chem 247: 667-679.

45. Mulquiney PJ, Kuchel PW (1999) Model of 2,3-bisphosphoglycerate metabolism in the human erythrocyte based on detailed enzyme kinetic equations: equations and parameter refinement. Biochem J 342 Pt 3: 581-596.

46. Chang A, Scheer M, Grote A, Schomburg I, Schomburg D (2009) BRENDA, AMENDA and FRENDA the enzyme information system: new content and tools in 2009. Nucleic Acids Res 37: D588-592.

47. Kramer R, Klingenberg M (1982) Electrophoretic control of reconstituted adenine nucleotide translocation. Biochemistry 21: 1082-1089.

48. Duyckaerts C, Sluse-Goffart CM, Fux JP, Sluse FE, Liebecq C (1980) Kinetic mechanism of the exchanges catalysed by the adenine-nucleotide carrier. Eur J Biochem 106: 1-6.

49. Barbour RL, Chan SH (1981) Characterization of the kinetics and mechanism of the mitochondrial ADP-atp carrier. J Biol Chem 256: 1940-1948.

50. Metelkin E, Goryanin I, Demin O (2006) Mathematical modeling of mitochondrial adenine nucleotide translocase. Biophys J 90: 423-432.

51. Al-Shawi MK, Ketchum CJ, Nakamoto RK (1997) The Escherichia coli FOF1 gammaM23K uncoupling mutant has a higher K0.5 for Pi. Transition state analysis of this mutant and others reveals that synthesis and hydrolysis utilize the same kinetic pathway. Biochemistry 36: 12961-12969.

52. Weber J, Senior AE (2000) ATP synthase: what we know about ATP hydrolysis and what we do not know about ATP synthesis. Biochim Biophys Acta 1458: 300-309.

53. Ferguson SJ (2000) ATP synthase: what dictates the size of a ring? Curr Biol 10: R804-808.

54. Tomashek JJ, Brusilow WS (2000) Stoichiometry of Energy Coupling by Proton-Translocating ATPases: A History of Variability. J Bioenerg Biomembr 32: 493-500.

55. Guerra G, Martinez F, Pardo JP (2002) On the H^+^/2e^-^ Stoichiometry of the Respiratory Chain. Biochemistry and Molecular Biology Education 30: 363-367.

56. Schwerzmann K, Hoppeler H, Kayar SR, Weibel ER (1989) Oxidative capacity of muscle and mitochondria: correlation of physiological, biochemical, and morphometric characteristics. Proc Natl Acad Sci U S A 86: 1583-1587.

57. Kroger A, Klingenberg M (1973) The kinetics of the redox reactions of ubiquinone related to the electron-transport activity in the respiratory chain. Eur J Biochem 34: 358-368.

58. Bose S, French S, Evans FJ, Joubert F, Balaban RS (2003) Metabolic network control of oxidative phosphorylation: multiple roles of inorganic phosphate. J Biol Chem 278: 39155-39165.

59. Belevich I, Verkhovsky MI, Wikstrom M (2006) Proton-coupled electron transfer drives the proton pump of cytochrome c oxidase. Nature 440: 829-832.

60. Beard DA (2005) A biophysical model of the mitochondrial respiratory system and oxidative phosphorylation. PLoS Comput Biol 1: e36.

61. Garlid KD, Beavis AD, Ratkje SK (1989) On the nature of ion leaks in energy-transducing membranes. Biochim Biophys Acta 976: 109-120.

62. Garlid KD (1990) New insights into mechanisms of anion uniport through the uncoupling protein of brown adipose tissue mitochondria. Biochim Biophys Acta 1018: 151-154.

63. Jezek P, Mahdi F, Garlid KD (1990) Reconstitution of the beef heart and rat liver mitochondrial K+/H+ (Na+/H+) antiporter. Quantitation of K+ transport with the novel fluorescent probe, PBFI. J Biol Chem 265: 10522-10526.

64. Li XQ, Hegazy MG, Mahdi F, Jezek P, Lane RD, et al. (1990) Purification of a reconstitutively active K+/H+ antiporter from rat liver mitochondria. J Biol Chem 265: 15316-15322.

65. Garlid KD (2000) Opening mitochondrial K(ATP) in the heart--what happens, and what does not happen. Basic Res Cardiol 95: 275-279.

66. Brierley GP, Jung DW (1990) Kinetic properties of the K+/H+ antiport of heart mitochondria. Biochemistry 29: 408-415.

67. Garlid KD (1980) On the mechanism of regulation of the mitochondrial K+/H+ exchanger. J Biol Chem 255: 11273-11279.

68. Garlid KD, Paucek P (2003) Mitochondrial potassium transport: the K(+) cycle. Biochim Biophys Acta 1606: 23-41.

69. Jung DW, Brierley GP (1999) Matrix free Mg(2+) and the regulation of mitochondrial volume. Am J Physiol 277: C1194-1201.

70. Mironova GD, Negoda AE, Marinov BS, Paucek P, Costa AD, et al. (2004) Functional distinctions between the mitochondrial ATP-dependent K+ channel (mitoKATP) and its inward rectifier subunit (mitoKIR). J Biol Chem 279: 32562-32568.

71. Dash RK, Beard DA (2008) Analysis of cardiac mitochondrial Na+-Ca2+ exchanger kinetics with a biophysical model of mitochondrial Ca2+ handling suggests a 3:1 stoichiometry. J Physiol 586: 3267-3285.

72. Paucek P, Mironova G, Mahdi F, Beavis AD, Woldegiorgis G, et al. (1992) Reconstitution and partial purification of the glibenclamide-sensitive, ATP-dependent K+ channel from rat liver and beef heart mitochondria. J Biol Chem 267: 26062-26069.

73. Bajgar R, Seetharaman S, Kowaltowski AJ, Garlid KD, Paucek P (2001) Identification and properties of a novel intracellular (mitochondrial) ATP-sensitive potassium channel in brain. J Biol Chem 276: 33369-33374.

74. Bragadin M, Pozzan T, Azzone GF (1979) Kinetics of Ca2+ carrier in rat liver mitochondria. Biochemistry 18: 5972-5978.

75. Scarpa A, Graziotti P (1973) Mechanisms for intracellular calcium regulation in heart. I. Stopped-flow measurements of Ca++ uptake by cardiac mitochondria. J Gen Physiol 62: 756-772.

76. Vinogradov A, Scarpa A (1973) The initial velocities of calcium uptake by rat liver mitochondria. J Biol Chem 248: 5527-5531.

77. Wingrove DE, Amatruda JM, Gunter TE (1984) Glucagon effects on the membrane potential and calcium uptake rate of rat liver mitochondria. J Biol Chem 259: 9390-9394.

78. Hinkle PC (2005) P/O ratios of mitochondrial oxidative phosphorylation. Biochim Biophys Acta 1706: 1-11.

79. Reeves JP, Condrescu M (2003) Allosteric activation of sodium-calcium exchange activity by calcium: persistence at low calcium concentrations. J Gen Physiol 122: 621-639.

80. Kapus A, Ligeti E, Fonyo A (1989) Na+/H+ exchange in mitochondria as monitored by BCECF fluorescence. FEBS Lett 251: 49-52.
